# Pharmacovigilance of Biopharmaceuticals in Rheumatic Diseases, Adverse Events, Evolution, and Perspective: An Overview

**DOI:** 10.3390/biomedicines8090303

**Published:** 2020-08-23

**Authors:** Sandra Rodríguez, Andrés Muñoz, Rosa-Helena Bustos, Diego Jaimes

**Affiliations:** Evidence-Based Therapeutics Group, Clinical Pharmacology, Universidad de La Sabana, Chía 140013, Colombia; sandrarope@unisabana.edu.co (S.R.); andresmuce@unisabana.edu.co (A.M.); diegojf@unisabana.edu.co (D.J.)

**Keywords:** biopharmaceuticals, biologics, monoclonal antibodies, pharmacovigilance, rheumatologic diseases, biosimilars

## Abstract

Since we have gained an understanding of the immunological pathophysiology of rheumatic diseases such as rheumatoid arthritis and systemic lupus erythematosus, treatment based on biological drugs has become a fundamental axis. These therapies are oriented towards the regulation of cytokines such as tumour necrosis factor-alpha (TNF-α), interleukin (IL)-6, IL-1, and the modulation of cell-mediated immunity (B cells and T cells) by anti CD20 or anti CTAL-4 agents, and can increase the risk of associated infections or adverse events (AE). In this context, the entry of biotherapeutics represented a challenge for pharmacovigilance, risk management and approval by the main global regulatory agencies regarding biosimilars, where efficacy and safety are based on comparability exercises without being an exact copy in terms of molecular structure. The objective of this review is divided into three fundamental aspects: (i) to illustrate the evolution and focus of pharmacovigilance at the biopharmaceutical level, (ii) to describe the different approved recommendations of biopharmaceuticals (biological and biosimilars) and their use in rheumatic diseases (RDs) such as rheumatoid arthritis (RA), juvenile idiopathic arthritis (JIA), psoriatic arthritis (PsA), ankylosing spondylitis (AS), systemic lupus erythematosus (SLE) and other less frequent RD like cryopyrin-associated autoinflammatory syndromes (CAPS), and (iii) to identify the main AE reported in the post-marketing phase of RD biopharmaceuticals.

## 1. Introduction

Biological drugs, biopharmaceuticals (BP), or macromolecules, as different authors have named them, refer to therapeutic schemes based on proteins. The World Health Organisation (WHO) has called them biotherapeutic products, obtained by applying biotechnology derived from various biological sources, initially non-human sources (animals, fungi, bacteria, and yeasts). However, after the complete sequencing of the human genome in 2004, it was possible to obtain 100% human therapeutic proteins [1,2]. According to the WHO definition, biotherapeutics are complex structures, ‘composed of sugars, proteins, or nucleic acids or complex combinations of these substances, or they can be living entities such as cells and tissues’, which include vaccines, blood components, tissues, and therapeutic recombinant proteins among others [3].

There are great differences between chemically synthesised drugs and BPs such as the origin, molecular size, complexity of the molecular structure, stability, the possibility of making exact copies, differences in the route of administration (usually the parenteral route), lability, and in production, handling and storage processes [4,5]. In addition, ‘biosimilar’ drugs have created a new challenge since, if they demonstrate high similarity, phase III clinical studies can be omitted, raising concerns about their efficacy, long-term safety, and increased risk of immunogenicity [2]. The latter is one of the adverse drug reactions (ADR) induced by biologics where agencies such as the WHO, the European Medicines Agency (EMA), and others that regulate biosimilars in Canada, South Korea and Australia have established that the pharmaceutical industry must be prepared to manage these ADRs through an established risk management plan [2].

Within biological risk management, pharmacovigilance (PV) plays a fundamental role in minimising these adverse events (AE). An example of the importance of PV in biotherapeutics was seen in the actions taken to prevent pure red cell dysplasia induced by the biological erythropoietin (EPO)-α (Eprex). The patients who received this biopharmaceutical created antibodies (Abs) that neutralised both exogenous and endogenous EPO, generating a cross-reaction. The reason was that the syringes, loaded with polysorbate and glycine instead of albumin (initially used as the adjuvant), reacted with the caps of the medicine package, generating these secondary Abs [6]. From the actions implemented through PV, preventive actions were taken and the control and regulatory agencies contraindicated the administration of EPO-α at the subcutaneous level, since this increased immunogenicity, thus reducing the number of cases that presented [4,7]. In this way, expanding the knowledge and understanding of the activities established by the relevant groups of PV in biologicals at the level of rheumatic diseases (RDs) could provide an overview of the real situation regarding AE associated with these biologicals and implement prevention tools to obtain a favourable clinical outcome for patients.

The objective of this review is to show the changes in the approach and performance of PV in biological therapy, the current situation of the PV system in BP in RD and the main AE reported by biologics for this type of disease.

## 2. Methodology

### 2.1. Search Eligibility Criteria

We included post-marketing studies on biotherapeutic drugs used for the treatment of RD: rheumatic disease (RA), juvenile idiopathic arthritis, (JIA), psoriatic arthritis (PsA), ankylosing spondylitis (AS), ankylosing spondylitis (SLE), and cryopyrin-associated autoinflammatory syndromes (CAPS). Only articles in Spanish, English, German, and French were included. News articles, phase I-III studies, and experimental studies were excluded from the review.

### 2.2. Search Strategy, Study Selection, and Data Extraction

The search was carried out in four stages. (i) We identified studies from SCOPUS, OVID, PubMed, LILACS, *Generics and Biosimilar Initiative Journal* (GaBi), Google Scholar, and OpenGrey, EMBASE. (ii) We reviewed the published evidence related to recommendations by regulatory agencies responsible for PV in Australia, Brazil, Canada, China, Europe, France, Germany, Ireland, Italy, Japan, Korea, Mexico, The Netherlands, New Zealand, Nigeria, Singapore, Sweden, the United States (US), and Colombia. (iii) The rheumatology societies of Brazil, Mexico, Portugal, Germany, Spain, America, the United Kingdom, Italy, and the Middle East were consulted. Finally, (iv) the main symposia and the abstracts of biotherapeutic congresses were searched.

**Figure 1:**
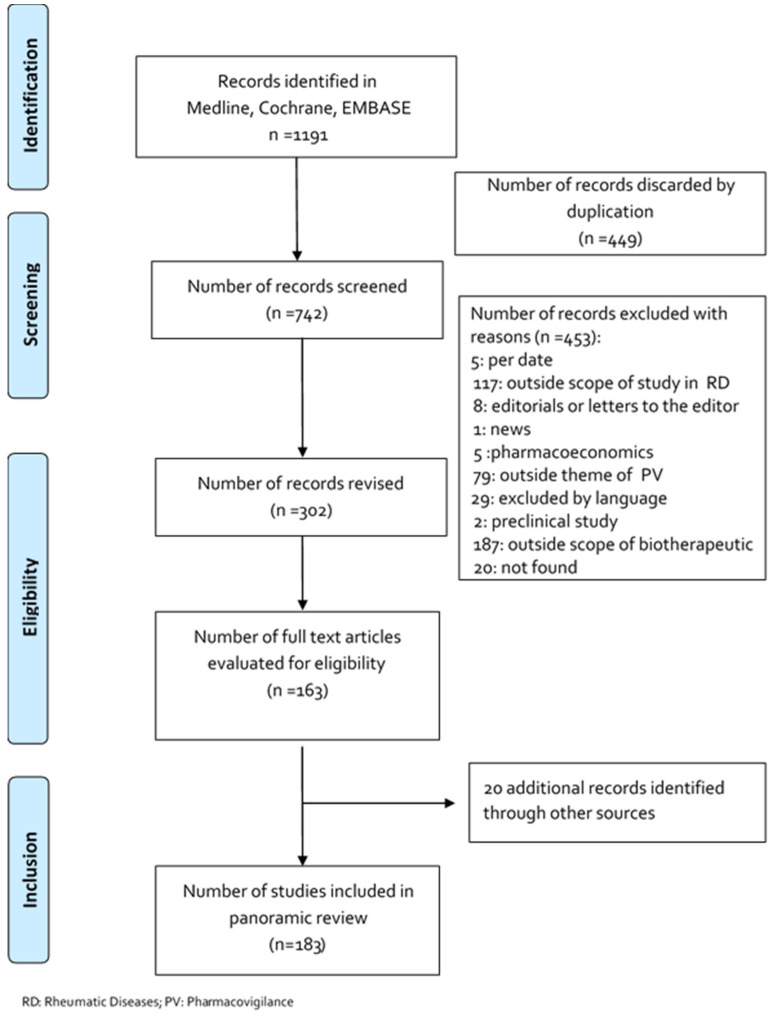
Flowchart depicting the selection process for the studies included in the paper.

The electronic search strategy is presented Table 1. Articles published from January 1990 to January 2020 were included. Two independent researchers performed the relevance screening. Relevant articles were reviewed to determine whether they met the eligibility criteria. Discrepancies were resolved through consensus. A broad overview of the search strategy is schematically presented in Figure 1.

## 3. Challenges Associated with the Pharmacovigilance of Biopharmaceuticals for Rheumatic Diseases

### 3.1. Importance of Pharmacovigilance for the Minimisation of Adverse Drug Reactions

According to the WHO, PV is defined as the ‘detection, evaluation, understanding, and prevention of adverse drug events or any other related problem’ [8]. This definition agrees with what is stipulated by the EMA, which defines PV as the ‘process and science of monitoring the safety of drugs and taking measures to reduce the risks and increase the benefits of drugs’ [9]. The WHO definition includes the term drug-related problem (DRP), which is defined by the second Granada consensus as negative clinical results, derived from pharmacotherapy and produced by various causes, leading to the non-achievement of the therapeutic objective or to the appearance of unwanted effects [10].

The best-known case of a DRP was related to the thalidomide tragedy in 1961. In this year, the PV system was created, and sought to offer an organised method where risks are identified and the population is notified early, decreasing the number of cases and ensuring the safety of patients [11,12]. In 1968, resolution 16.36 of the 16th WHO World Assembly considered the need to create an information system on ADRs occurring worldwide, which is why the Program for International Drug Monitoring (PIDM) and the database were created. VigiBase is powered by each of the 136 member countries and administered by the Uppsala Monitoring Centre and collaborating centres in India, Morocco, and Norway [13,14]. The objective of this database is to ‘promptly recognise signs that indicate serious ADR to a medicine, evaluate the dangers, and investigate mechanisms of action to contribute to the development of safer and more effective medicines’ [15].

VigiBase has several tools such as VigiLyze and VigiFlow; the first tool provides a general approach to VigiBase data and is designed for PV program affiliates and national centres, while VigiFlow handles the individual case safety reporting (ICSR) database and notifications for centres that do not have their own national database. This system uses the VigiAccess interface, which allows anyone to access suspected ADRs without revealing the personal data of the reported cases [16].

The American College of Rheumatology (ACR), since 2014, has implemented the Rheumatology Informatics System for Effectiveness (RISE). This is a database that extracts electronic medical records related to rheumatology [17]. On the other hand, Latin America and the Caribbean community (CARICOM), made up of 20 developing countries, can use the VigiCarib regional system created in December 2017 to report ADR and changes in the quality of pharmaceutical products [18]. For our case of interest, the biotherapeutic products used in RD are included in databases established by the Spanish rheumatology society BIOBADASER and in the US (the BIOBADAMERICA system), fed by the registries of each country. Similar systems are in use in Argentina (BIOBADASAR II), in Mexico (BIOBADAMEX), in Uruguay and Paraguay (BIOBADAGUAY), and Brazil (BIOBADABRASIL). The latter has a patient comparator group, which receives treatment with traditional disease-modifying antirheumatic drugs (DMARDs) [16,19,20,21,22]. Countries such as Australia, through the Australian Rheumatology Association database (ARAD), monitor the outcomes of patients with RD, mainly if they undergo treatment with biotherapeutics [23]. All the aforementioned databases are fed with ICSRs, which are the documents with the most complete information, provided by a primary source to describe suspected ADRs, related to the administration of one or more medications to a patient at any given moment [24,25,26].

**Figure 2:**
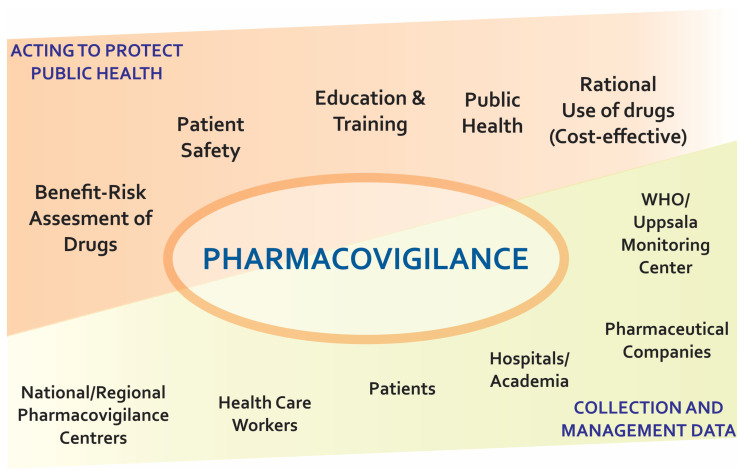
Main actors in the pharmacovigilance process.

ADR, according to the WHO, is a harmful, unintended response to authorised doses, which may be dose dependent or independent, and which may or may not occur unpredictably. An adequate PV system allows the identification of rare and severe ADR signals, making it easier to establish dose-dependent reactions, called type A or with increased pharmacological effect, and ADR type E, because they occur at the end of treatment. Notwithstanding, type B ADRs are generally identified post-marketing because they are idiosyncratic and associated with patient and/or environmental characteristics. In the case of ADR type D, occurring long after treatment has ceased, and ADR type C, which appears chronically, often an association with a drug cannot be identified [27,28]. The identification of possible ADRs can be carried out through two processes, the first through passive surveillance, with spontaneous notification to the WHO or regional control centres, or by physical or electronic methods, i.e., by filling out instruments such as the ‘yellow card’. The second process corresponds to an active system, where patients managed with a certain drug are followed up. This process has the advantage of having more complete patient data and ease of identifying AE, as well as data on the subgroups with the highest risk of presenting ADR [29,30]. Figure 2 shows the different actors involved in the PV process.

PV has two phases: the first phase is risk analysis, where a risk is identified, measured, and evaluated based on the identification of a signal, defined as information about a possible causal relationship between an ADR with a drug. The second phase, called risk management, is responsible for the implementation and monitoring of regulatory measures [10,12]. In this latter phase, the risk management plan (RMP) plays a fundamental role. This is a document maintained by the pharmaceutical industry, where the actions necessary to identify, characterise, and minimise the important risks for a drug are captured [26]. The European Commission (EC) indicates that this process must have a communication system with each of the parties and proper monitoring of the outcomes of the process [9,31].

### 3.2. Changes in the Approach and Implementation of Pharmacovigilance in Biological Therapy

Since the first detection and the establishment of ADR causality associated with different therapeutic strategies and in the understanding of the concept of PV in different countries and organisations, multiple activities have been generated to increase the safety of medications. After the formation of the first regulatory agencies in individual nations and the formation of cross-cutting entities worldwide by the WHO, the leadership of PV has also been assumed by different local and continental actors aimed at special groups oriented by pathologies and therapeutic groups.

**Figure 3:**
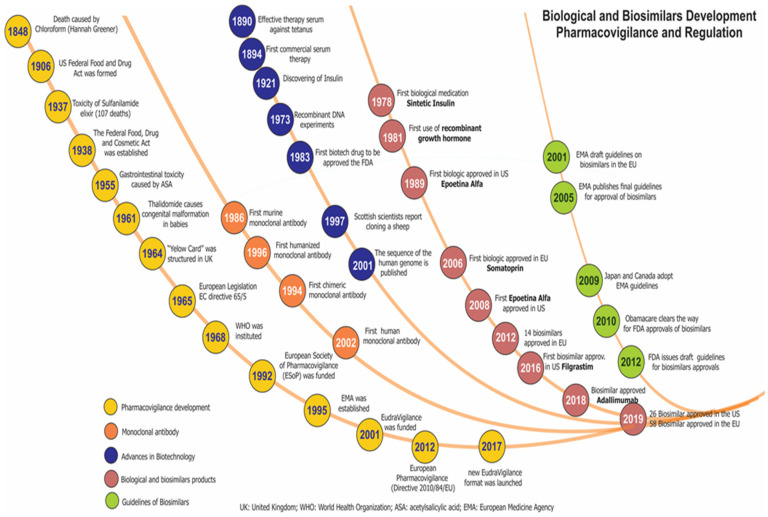
Timeline of pharmacovigilance and regulation of biopharmaceuticals and biosimilars.

As well, with the continuous development of new therapeutic agents, including chemically synthesised drugs and the first biotherapeutics, new challenges have been generated in PV, added to the need to establish additional measures and guidelines for the detection and management of the risk of associated ADRs. Given these needs, the EMA in 2012 established new regulations on PV and the formation of EudroVigilancE (European database for suspected adverse drug reaction reports), as well as the creation by different scientific groups of follow-up cohorts in biotherapeutics in Europe such as Rheumatoid Arthritis Observation of Biologic Therapy -RABBIT (Germany), AntiRheumatic Therapy-ARTIS (Sweden), British Society for Rheumatology Biologics Register for RA-BSRBR_RA (United Kingdom), Spanish Registry of adverse events of biological therapies in rheumatic diseases-BIOBADASER and Group of Biological Agents in Autoimmune Diseases-BIOGEAS (Spain), and Group for the Study of Early Arthritis-GISEA (Italy). In addition to these groups, patients play an important role in the identification of ADRs since they use social networks to increase the detection of early alerts in possible outcomes of ineffectiveness or adverse effects. Figure 3 presents the evolution of PV regulation for biological and biosimilar drugs. The main problems in the PV process are associated with cultural differences in medical practice, a lack of resources for regulatory activities, and little experience or interest among clinicians in performing PV [32].

## 4. Biopharmaceuticals in Rheumatic Diseases

RDs are a group of approximately 200 chronic pathologies of the musculoskeletal system. It is estimated that about 10% of the population suffers from a rheumatic disease. In Asia, these diseases are estimated to have the same prevalence as in the Western world, but have been reported to be more aggressive [33]. One of the problems with this group of diseases is the deterioration in quality of life, leading to physical and economic dependency, a loss of job opportunities, isolation and limitations in carrying out the activities of daily life [34]. The main RDs managed with biotherapeutics are RA, JIA, PsA, AS, SLE, and CAPS [35]. Table S1 summarises the main biological, biosimilar, and copy attempts of the main RDs [35,36,37,38,39,40,41,42,43].

### 4.1. Rheumatoid Arthritis

RA is a chronic, symmetrical inflammatory disease that attacks multiple joints, causing deformity, pain, and loss of function if proper treatment is not received [44]. It is of multiple aetiologies and can produce multiple manifestations at the extra-articular level. The diagnostic criteria published in 2010 by the ACR and the European League Against Rheumatism (EULAR) assess the number of joints involved, the chronicity of pain, and the patient’s paraclinical. The incidence of this disease is close to 40 per 100,000 people, with a female to male ratio of three to one [45,46]. Early treatment with DMARDs reduces limitation and disability. DMARDs are immunosuppressive drugs, and can be either traditional or biological [47]. The biological drugs used for the management of RA are tumour necrosis factor inhibitors (anti-TNF-α), interleukin (IL)-1 inhibitors, IL-6 inhibitors, T lymphocyte inhibitors, and B lymphocyte inhibitors.

Anti-TNF-α agents act by neutralising soluble and membrane TNF-α, preventing its binding with the Tumor Necrosis Receptors (TNFR)-I (p75) and TNFR-II (p55). This induces apoptosis, stops the cell cycle, inhibits angiogenesis and the inflammatory cascade at the synovial level, and suppresses chondrocytes and osteoclasts, thus reducing bone resorption and erosion [48]. The etanercept fusion protein and five different monoclonal antibodies (mAbs) are currently approved: adalimumab, certolizumab pegol, infliximab, tocilizumab, and golimumab. Infliximab was approved in 1999 in the USA as monotherapy for people who do not respond to methotrexate treatment, and in 2001 the joint management of these two drugs was approved. This biological has multiple biosimilars approved for commercialisation; among them is SB4, commercialised in Europe as Eticovo, in the USA as Benepali, and in Korea, Australia and Brazil with the name of Brenzys [28]. CT-P13 is marketed as Inflectra and Remsima. Golimumab was approved by the Food and Drug Administration (FDA) as Simponi in 2009 for subcutaneous administration and under the name of Simponi Aria in 2013 for intravenous administration [35].

IL-1 inhibitors block the receptors IL-1RI and IL-1RII, decreasing acute phase reactions. The exponent of this approved Anakinra group is a competitive inhibitor, a recombinant non-glycosylated analogue of IL-1Rα. IL-1 stimulates colony-stimulating factor (CSF) expression and activates endothelial cells, synovial fibroblasts, chondrocytes, osteoclasts, and components of the immune system, especially neutrophils [36].

Blocking IL-6 decreases T cell proliferation, B cell differentiation, and macrophage activation. Representatives of this group are sarilumab (human mAb) and tocilizumab (humanised mAb), which bind to sIL-6R and mIL-6R receptors, thereby decreasing levels of immunoglobulins (Ig) and suppressing the joint and systemic inflammatory response.

T lymphocyte inhibitors are represented by abatacept, a fusion protein composed of the domain associated with cytotoxic T-lymphocyte antigen 4 (CTLA-4), which works by preventing the second signal or costimulatory signal, thereby inhibiting the stimulation of T lymphocytes [35,48]. B cell blockers, such as rituximab, act by blocking CD20, which prevents the intermediate stage of B cell development, thus decreasing the number of these cells at the bone and synovial level [36].

### 4.2. Juvenile Idiopathic Arthritis

JIA corresponds to a heterogeneous group of persistent arthritis syndromes, which begin in children under 16 years of age with a minimum of 6 weeks of evolution; the aetiology is unknown. JIA requires a clinical diagnosis, using the criteria of the International League of Associations for Rheumatology (ILAR) from 2001. In systemic JIA, arthritis with the involvement of one or more joints, associated with fever on a daily basis, must be present for at least three consecutive days, lasting at least two weeks and accompanied by serositis, lymphadenopathy, evanescent erythematous exanthema, and hepato- or splenomegaly. Its most frequent extra-articular manifestation is uveitis. The prevalence of this group of diseases is from 37 to 84 cases per 100,000 children under 16 years of age [49,50,51]. Treatment is intraarticular corticosteroid infiltration alone or accompanied by a DMARD such as methotrexate or leflunomide and to a lesser extent sulfasalazine. In cases with no response, the use of the biologicals etanercept, canakinumab, tocilizumab, or abatacept is authorised [35,50,52,53]. Canakinumab is an mAb agonist for IL-1β, a proinflammatory cytokine that regulates innate immunity [36].

### 4.3. Psoriatic Arthritis

PsA is a chronic inflammatory disease where the Classification Criteria for Psoriatic Arthritis (CASPAR) criteria are used to evaluate the evidence of psoriatic dactylitis, nail dystrophy, and bone formation at the juxtaarticular level, accompanied by a negative test for rheumatoid factor. An incidence of 83 cases per 100,000 people/year and a prevalence of 133 cases per 100,000 people/year are estimated. The treatment proposed by the EULAR is to start with topical treatment for psoriasis and non-steroidal anti-inflammatory drugs; if required, continue with a DMARD such as methotrexate, sulfasalazine or leflunomide; if no response is observed, provide combinations of DMARDs and finally, if necessary, use the biologics adalimumab, certolizumab pegol, etanercept, infliximab, or ixekizumab. The latter is a humanised mAb that binds to the proinflammatory cytokines IL-17 A and IL-17 A/F [36,54,55,56].

### 4.4. Ankylosing Spondylitis

AS is a chronic autoimmune inflammatory disease that affects the axial skeleton, but can also affect peripheral joints and other organs. The diagnosis is made according to back pain inflammatory characteristics, acute phase reactants, and elevated human leukocyte antigen (HLA)-B37, associated with radiological alterations in the spine, in patients younger than 40 years of age. The first-line treatments are non-steroidal anti-inflammatory drugs (NSAIDs), physical therapy, and intra-atrial steroids. DMARDs such as methotrexate and leflunomide have no evidence supporting their use in this pathology, but sulfasalazine has shown satisfactory results in some studies. In case of no response, the approved biologics are anti-TNF-α, etanercept, adalimumab, certolizumab pegol, infliximab, and golimumab [35,57].

### 4.5. Systemic Lupus Erythematosus

SLE is an autoimmune disease that can compromise any organ. It has a prevalence of 3–500 per 100,000 people and mainly affects women between 16–55 years of age. Antinuclear antibodies (ANAs) and immunoassay tests are a diagnostic support for this pathology. The scales most used for its classification are the ACR and EULAR criteria updated in 2019, assessing the presence of fever, joint, skin, synovial, neurological, haematological, and renal involvement, as well as laboratory findings (antibodies against phospholipids are evaluated as well as ANAs) [58].

The British Society of Rheumatology (BSR) published guidelines for the treatment of SLE in 2018, where it stipulates that, depending on the activity of mild, moderate, or severe disease, induction can be performed with NSAIDs, cyclooxygenase inhibitors, local or systemic glucocorticoids, DMARDs such as methotrexate, azathioprine, mycophenolate mofetil, or cyclophosphamide. Likewise, they indicate that maintenance can be performed with hydroxychloroquine, the biotherapeutics belimumab and rituximab, or the drugs mentioned in induction, except for cyclophosphamide [35,51]. In addition to the biologicals mentioned previously, ocrelizumab is also authorised. It is key to bear in mind that there are several biotherapeutics under study for the treatment of this pathology, i.e., epratuzumab, abetimus, edratide, belimumab, atacicept, efalizumab, sifalimumab, rontalizumab. Moreover, there are biologics approved for other pathologies that are currently being evaluated for the management of SLE, such as anakinra, tocilizumab, infliximab, and abatacept [59].

### 4.6. Other Inflammatory Syndromes

Other biologically managed rheumatologic diseases are CAPS, which groups diseases caused by mutations in the NLRP3 gene. CAPS include neonatal onset multisystemic inflammatory disease, Muckle–Wells syndrome (MWS), and familial cold autoinflammatory syndrome (FCAS). The use of the IL-1 inhibitor rilonacept (a fusion protein with high affinity for the homologous form of IL-1β and to a lesser extent for IL-1α) was approved for these pathologies [60]. It is currently distributed in the USA, but in Europe it was withdrawn from the market on 20 September 2012 for commercial reasons [61].

According to what is reported in Table 1, the use of biotherapeutics varies in each country, according to the national databases; for example, in Argentina, etanercept is predominantly used at 25.12% and adalimumab at 13.3%; in Brazil, infliximab at 39.0% and adalimumab at 28.0%; in Paraguay–Uruguay, adalimumab at 56.5% and etanercept at 23.7%, in Mexico and China, etanercept at 25.6% and 35% and infliximab at 19.8% and 17%, respectively [37,62].

In the USA, on average, 40% of RD patients receive biological treatment, in contrast to the Middle East and north Africa where coverage is only 2% [33,63]. The factors considered for this inequality may be associated with the difficult access of BP and with the difficulties in monitoring treatment with biologicals. Additionally, in these regions, there are epidemics such as tuberculosis, making this therapy contraindicated [33]. In Latin America, access varies according to the biologicals, as 76% of children with SLE are treated with rituximab while for belimumab coverage is only 11% [57,58]. Some countries such as Cuba only have some biotherapeutics authorised, as can be seen when consulting the Centre for State Control of Medicines, Equipment and Medical Devices (CECMED) for RD, which mentions rituximab and tocilizumab [64].

Within the coverage of biosimilars for indications of different RDs, even to date the change or substitution (interchangeability) of an innovative biotherapeutic for any biosimilar has not been approved [28]. Interchangeability is a characteristic between two or more products, which allows for a substitution between them. According to the FDA, in the Biologics Price Competition and Innovation Act (BPCIA) of 2009, manufacturers must supply sufficient information to indicate the biosimilarity of the biotherapeutic, including the same clinical result as the reference product in any patient. Equally, if there is a change from an innovator to a biosimilar, it must be demonstrated that the administration of the biosimilar will not affect the safety and efficacy of the innovator [63,65,66].

## 5. Adverse Events Reported Post-Marketing with Biopharmaceuticals in Rheumatic Disease

Among the main global concerns of PV are the post-marketing changes of biotherapeutics. These changes can be low risk (changes in the manufacturing process of the final product, changes in the packaging of the final product), moderate risk (changes in the process of or analysis during the manufacture of the active substance), and high risk (changes in purification, lot size, detection limits of the active substance). These modifications mean that biotherapeutics have an impact on the safety and efficacy of the medicine and that they can present AE in patients. In the case of biotherapeutics for the treatment of RD, Vezér et al. evaluated the changes in the production of the original mAbs authorised by the documents of the European public assessment report (EPAR) from 1998 to 2014 (Table 2) [67]. Principal AE in RDs associated with biological drug treatment are presented in Table 3.

### 5.1. Postmarketing Reported Adverse Events with Biopharmaceuticals

**Figure 4:**
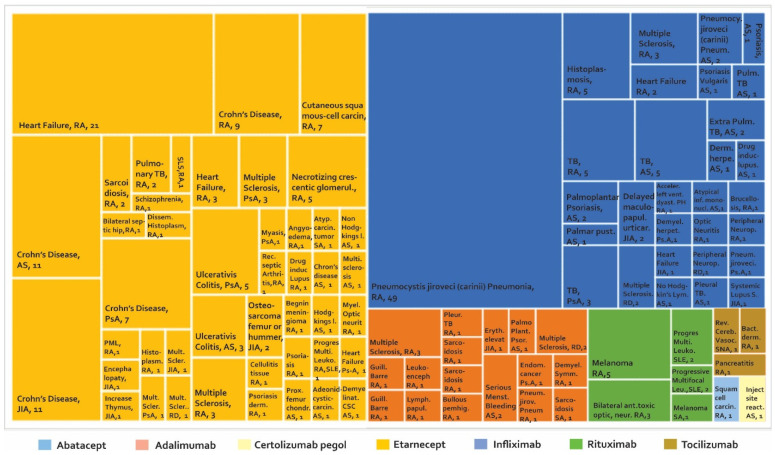
Adverse biotherapeutic events in rheumatic diseases presented in case reports and case series [69,70,71,72,73,74,75,76,77,78,79,80,81,82,83,84,85,86,87,88,89,90,91,92,93,94,95,96,97,98,99,100,101,102].

**Figure 5:**
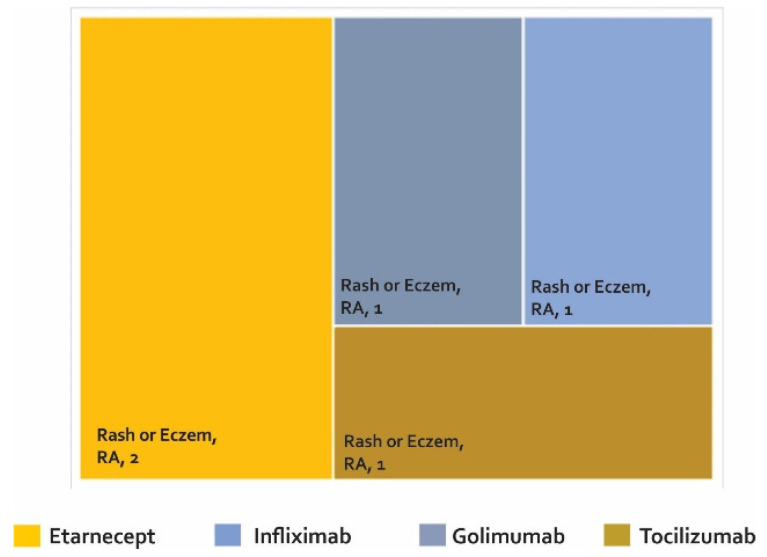
Adverse biotherapeutic events in rheumatic diseases presented in cross-sectional studies [103].

**Figure 6:**
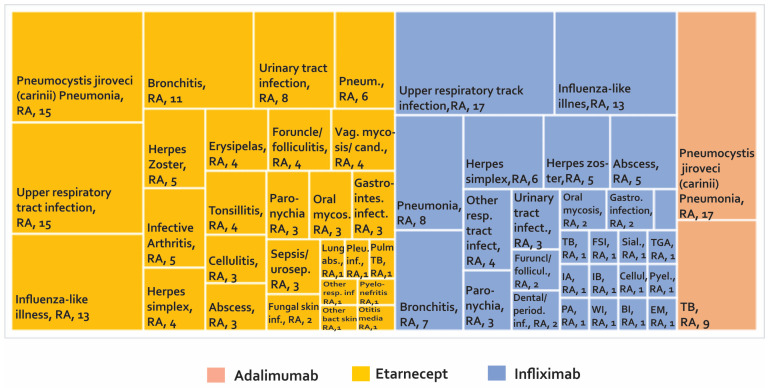
Adverse biotherapeutic events in rheumatic diseases presented in case-control studies [104,105,106,107].

**Figure 7:**
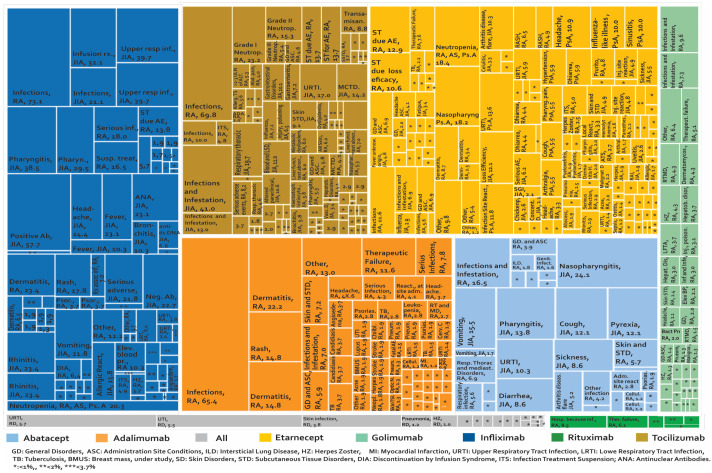
Adverse biotherapeutic events in rheumatic diseases presented in cohort studies (presented by percentages) [6,21,55,108,109,110,111,112,113,114,115,116,117,118,119,120,121,122,123,124,125,126,127,128,129,130,131,132,133,134,135,136,137,138,139,140,141,142,143,144,145,146,147,148,149,150,151,152].

**Figure 8:**
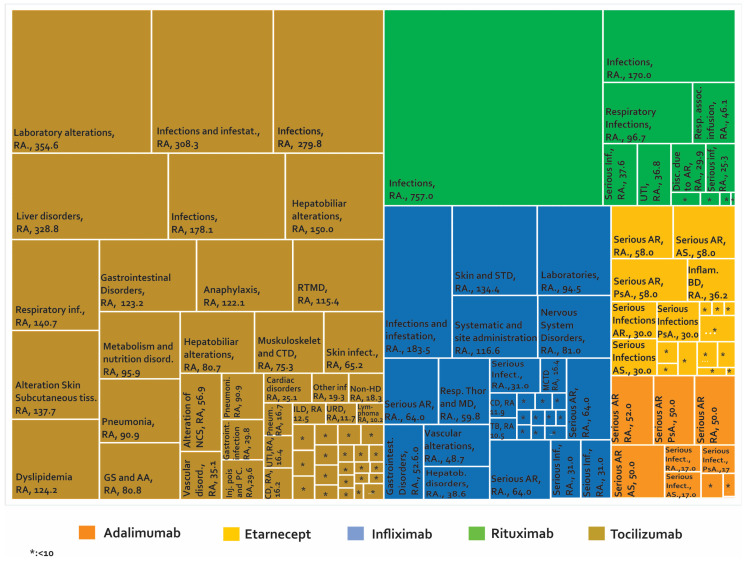
Adverse biotherapeutic events in rheumatic diseases presented in cases by 1000 patients/year [111,125,130,131,132,133,145,146,147,149,153,154,155,156,157,158,159,160,161,162,163,164,165,166,167,168,169].

Once the phase of controlled clinical trials has been passed and after approval by a regulatory entity, the number of people exposed to a drug increases exponentially. This could make the detection of AE easier, especially AE that perhaps were not identified in the early stages of development. It is estimated that the number of patients treated with etanercept after the approval of this biotherapeutic is more than 39 times the number of patients evaluated in clinical studies with RA, PsA, and JIA. For infliximab, it is estimated that 175 times the number of patients evaluated with RA in clinical studies have been monitored after marketing [68]. The development of phase IV studies makes new alerts visible in PV constructed from studies such as (i) case reports and case series (Figure 4), (ii) cross-sectional studies (Figure 5), (iii) cases and controls (Figure 6), and (iv) cohorts (presented by percentages) (Figure 7) and cases presented as 1000 patients/year (Figure 8). Supplementary Tables S2–S6 present the evidence found on the main AE documented in the post-marketing phase. The Table 3 shows the principal AE in RDs associated with biological drug treatment.

According to Figure 4 and supplementary Table S2, 102 AE are found in case series studies and case reports, with a higher number of reports for etanercept and infliximab. Among the AE that were presented most frequently, the following were found: (1) Crohn’s disease with more frequency of reporting in AS, JIA, PA with 11, 11, and seven cases, respectively, in patients treated with etanercept [92]. (2) *Pneumocystis jiroveci* (*carinii*) pneumonia was reported in RA with 49 cases in patients treated with infliximab, followed by Crohn’s disease in patients treated with etanercept in nine cases. (3) In seronegative arthritis, one case of reversible cerebral vasoconstriction was reported. (4) Thymic tumours, melanoma and sarcoidosis have been reported in spondyloarthropathy. (5) In SLE, three cases of progressive multifocal leukoencephalopathy (PML) with rituximab have been reported. (6) Studies on patients with RD reported two cases of multiple sclerosis with adalimumab, one with etanercept, and two with infliximab.

As can be seen in Figure 6 and supplementary Table S4, 80 AE have been reported in case-control studies. The most frequently reported AE are upper respiratory infection with infliximab in 17 cases and etanercept in 15 cases. Likewise, AE *Pneumocystis jiroveci* (*carinii*) pneumonia was reported in 17 cases with adalimumab and 15 cases with etanercept, followed by influenza-like illness in 13 cases with both infliximab and etanercept.

Figure 7, Figure 8 and supplementary Tables S5 and S6 show 267 AE reported in cohort studies. Figure 7 shows the studies in which the AE were analysed as a percentage of presentation, while Figure 8 shows the studies that used the prevalence rate as an indicator (cases presented per 1000 patients/year). Analysis of these data shows: (1) In AS, one case of peripheral neuropathy due to infliximab was reported, representing 1.3% of treated patients. (2) In JIA, 73.08% of the patients managed with infliximab had infections and 41.01% of the patients with tocilizumab presented infections and infestation. (3) In PsA, we only found one study carried out in Canada in 2011 for etanercept, where the most frequent AE was nasopharyngitis in 18.18%. (4) In AS, the rate of serious adverse reactions was 64 cases/1000 patients/year with infliximab and 58 cases/1000 patients/year with etanercept. (5) In JIA, the rate of inflammatory bowel disease as an AE from etanercept was 36.2 cases/1000 patients/year. (6) In PsA, serious adverse reactions were reported with a rate of 64 cases/1000 patients/year for infliximab and 58 cases/1000 patients/year with etanercept. (7) In RA treated with rituximab, infections were reported in 757 cases/1000 patients/year in international studies and decreased to 170 cases/1000 patients/year in a study carried out in Greece. When tocilizumab was evaluated, laboratory abnormalities were found in 354.6 cases/1000 patients/year. (8) Demyelination was reported in rheumatic diseases at a rate of 0.44 cases/1000 patients/year with infliximab and 0.43 cases/1000 patients/year with etanercept. (9) For spondylarthritis, five cases of myelitis with etanercept were reported in cohort studies.

Only one cross-sectional study was found (Figure 5 and supplementary Table S3), which found rash or eczema in two cases treated with etanercept and in one patient treated with golimumab, infliximab, and tocilizumab.

Additionally, studies were found as meta-analyses, where the presence of anti-drug antibody (ADA) was evaluated in patients with RA and treated with infliximab, etanercept, adalimumab, golimumab, tocilizumab, abatacept, and rituximab. The highest percentage of ADA found was for infliximab, with a range of 8–62% [153] and 26–52% [170] while the lowest percentage was found for golimumab with a range of 2–10% [170]. Another important variable is that each meta-analysis involves a different number of studies for each of the biotherapeutics. Infliximab had the largest number of included studies (48) while abatacept had only seven studies (Table S6). Interestingly, a narrative review study for tocilizumab for the treatment of RA was found, with a rate of 9.3 cases/1000 patients/year. These antibodies are rare in patients treated with abatacept or etanercept [170]. There is a clear association between the formation of ADA and the loss of efficacy of biotherapeutics, although in patients with RA treated with etanercept and certolizumab pegol, the evidence is not as robust [170]. In a study by Nikiphorou et al., they recommend measuring ADAs before switching to a biosimilar [171].

Other studies on AE were found in pregnant patients with RD and treated with biological therapies with tocilizumab or certolizumab pegol. The AE for tocilizumab were renal pielectasis, oesophageal fistula, cardiovascular malformations, and alterations in the development of the central nervous system (CNS), as well as reports of abortion [172,173]. In the case of certolizumab, the AE reported were abortion, in addition to accessory atrium, anal fistula, hydrocephalus, macrosomia, and polydactyly in neonates [174]. In infants, cetolizumab pegol is associated with respiratory infections, gastroesophageal reflux, and lichen striatus as AE, while in mothers, breast abscesses, respiratory infections, and headaches were reported [175].

All the studies had different methodological designs, as well as different outcomes and definitions of the AE and follow-up time, thereby limiting the definition of the most frequent ADRs.

There are other additional studies included in national databases of some countries. Such is the case of Japan, where the relationship between AE and etanercept and the duration of RA were analysed. Patients with a brief duration of this disease (less than 2 years) had 225 AE (31.8%) compared to patients who had the disease for 20 years, where 413 AE (37.9%) were reported, representing a statistically significant increasec [176]. In contrast, on the Dutch national basis it was found that most of the AE for etanercept in patients with JIA occurred in the first 15 months of treatment.

There are other reports of AE for etanercept for all pathologies including RD. Studies analysed the databases of Taiwan’s National Health Insurance (NHI) and death certificates, reporting four tuberculosis events in patients treated with etanercept, with an incidence rate of 679.5/100,000 people/year and a hazard ratio (HR) adjusted for 3.21 (95% CI (Confidence Intervale): 1.06–9.77) [133]. In this country, they also evaluated the risk of malignancy, finding a prevalence rate of 10,363.75 people/year, with an incidence rate of 6.85/1000 people/year. In Japan, the SECURE registry ‘safety of biologics in Clinical Use in Japanese patients with rheumatoid arthritis’ showed an unadjusted incidence rate of 7.57 (95% CI 6.77–8.85) per 1000 people/year for non-haematological malignancy [128,150,177]. In addition, in a retrospective study, therapeutic failure was reported in 23 cases of 109,335 treated patients, representing 0.02% [178,179,180,181]. Another study performed in Japan analysed the association between the use of this biotherapeutic and hepatitis B, but did not find a significant association, with an odds ratio of 0.8 (95% CI: 0.7–1.0) [182].

The Clinical Trials web page, currently reports four ongoing studies to monitor the efficacy and safety of Enbrel in people with RA; one of them is being performed in patients after infliximab failure and two are comparing biologicals with conventional treatment. A fifth study is evaluating the effects on endothelial function and blood pressure in patients with RA, PsA, or AS. A final ongoing study is evaluating cardiac function in patients with RA or AS treated with infliximab, adalimumab, and etanercept [183].

Regarding adalimumab, it has been associated with hepatitis B infection, as there were six cases reported by the FDA between 2004 and 2010, according to a case-control study, with an odds ratio (OR) of 0.1 (95% CI: 0.1–0.2) [182]. In addition, cases of multiple sclerosis (MS) (one case), nephrotic syndrome (one case), histoplasmosis (two cases in 31,448 patients treated until 30 June 2004), pulmonary nocardiosis (one case), listeriosis (one case), and systemic toxoplasmosis (one case) have been described [180,181,184,185]. Five tuberculosis (TB) AE have been reported in Taiwan in patients treated with anti-TNF-α, presenting an incidence rate of 354.3 people/year, an adjusted HR of 3.37 (95% CI: 1.12–10.17), and a rate of 6.85 per 1000 people/year [128,150]. Japan’s SECURE registry showed an unadjusted incidence of 9.61 (95% CI 6.57–13.61) per 1000 people/year for non-haematological malignancy [177]. At the moment, an AR study is being carried out on the effects of adalimumab on vascular abnormalities [183]. Additionally, the association between the use of this biotherapeutic and hepatitis B was analysed, finding a significant association with an odds ratio (OR) of 0.1 (95% CI: 0.1–0.2) [182].

Cases of SLE (three cases), systemic candidiasis (three by Keenan), systemic coccidioidomycosis, cryptococcosis, histoplasmosis (nine cases of Lee and two by Wood), and listeriosis (two cases, 28) have been reported in RA patients treated with infliximab (cases until 2002), along with sepsis due to *Staphylococcus aureus*, haemorrhagic colitis due to *Escherichia coli*, and the reactivation of brucellosis and tuberculosis (242 cases presented in 290,000 treated patients), calling attention to the atypical location [180,181]. A study is currently underway to evaluate the efficacy and safety of infliximab in this inflammatory disorder [186]. *Listeria monocytogenesis* meningitis has been reported in patients with JIA treated with infliximab (1 case) and listeriosis has been reported in patients with PsA (1 case) [180]. In Japan, an unadjusted incidence rate of 7.70 (95% CI 6.43–9.16) was observed per 1000 people/year for non-haematological malignancy and 3.38 (95% CI 2.57–4.38) for lymphoma [177].

With abatacept, a subgroup analysis was performed where AE such as severe infections, cancer, and death were found. These increase with age, leading to frequent stoppage of treatment [116]. An investigation carried out in 11 Italian rheumatology centres, with 72 patients who presented with hepatitis B among inactive carriers (occult or chronic hepatitis), found no reactivation of this infection after monitoring them for 24 months [187]. Currently, a study is being conducted in patients with RA on the relationship of abatacept and myocarditis [183]. An American study evaluated the association with cancer, finding an adjusted HR of 1.2 (95% CI: 1.03–1.39) for non-melanoma skin cancer, but this was not significant for breast cancer, lung cancer, lymphoma, or melanoma. When comparing abatacept with other biological DMARDs as initial biotherapeutics, no statistically significant difference was found in the risk of serious infections [163].

Regarding rituximab, most of the AE presented in the first 6 months, decreasing with each treatment cycle [113]. Infusion-related AE occurs more frequently in the first administration of the first cycle. An association has been found between this biological and urinary tract infection (UTI), anaemia, and leukopenia, with an increased risk of 1.7, 2.8, and five times, respectively [188]. A case-control study based on FDA reports between 2004 and 2010 found an association between rituximab and hepatitis B, with 12 reported cases and an OR of 7.2 (95% CI: 5.3–9.9) [182]. The association between this biotherapeutic and hepatitis B showed a significant association, with an OR of 7.2 (95% CI: 5.3–9.9) [182].

Other AE reported for rituximab are enterovirus myofasciitis, West Nile virus infectious polyneuropathy, encephalitis, John Cunningham virus, and progressive multifocal leukoencephalopathy [184]. When analysing the deaths in the population exposed to this biotherapeutic, 78 deaths were found, with 0.53 events/100 patients/year, which is the same rate adjusted for age and sex as the general population. It has not been observed to increase the risk of malignancy, even in the registry of Taiwan’s NHI, where there were no cases of cancer in patients with this biotherapeutic [149,150].

Currently, five studies are being carried out to evaluate the safety of tocilizumab compared to etanercept in RA patients, one analysing the long-term safety, another inflamed atherosclerotic plaques, a third the cardiovascular risk, a fourth the impact at the periodontal level, and the last assessing safety in patients with risk factors for cardiovascular disease. Two other studies are assessing efficacy and safety with and without methotrexate compared to adalimumab and etanercept [183]. A Japanese study reported an unadjusted incidence rate of 5.78 (95% CI 4.03–8.05) per 1000 people/year for non-hematologic malignancy for this drug [177].

According to an Argentine cohort with a population of 347 people, it was found that patients who suspended a biological DMARD due to the presence of AE tended to replace it with a second biological DMARD, even with a different mechanism of action, re-suspending it in a shorter period at four months [189].

### 5.2. Adverse Events Reported with Biosimilars

It has been considered that biosimilars can present different AE than those reported by the reference biologicals [190]. There is a theory that biosimilars in RD patients are more ‘immunologically active’, leading to more frequent AE [191]. Studies show that in patients with spondylarthritis managed initially with the infliximab innovator for more than 6 months and later with the CT-P13 biosimilar for 6 months, there were no statistically significant differences in the ARD presented. However, with the biosimilar, 1 case of severe palmar plantar psoriasis was reported after the second application of the biotherapeutic, so treatment was suspended [112]. One case of neurofibromatosis was also reported after the second dose of this biosimilar, which caused the innovative biotherapeutic to be administered again [171]. The Danish National Registry (DANBIO) reported a suspension of the biotherapeutic Benepali, a biosimilar of etanercept, due to a loss of effectiveness in 9.18% and AE in 4.74% [192].

### 5.3. Adverse Events Reported with ‘Intended Copies’

‘Intended copies’ or ‘non-comparable biologics’ are BPs that have undergone limited clinical trials to guarantee safety and efficacy or do not even a complete biocomparability study; that is, they have not been shown to have the same reliability as the reference biological. ‘Intended copies’ have no studies registered in Clinical Trials web page [39,183].

Etanar is an ‘intended copy’ described in the literature as a biotherapeutic with limited clinical studies, where equivalence has not been demonstrated. In Colombia, the National Food and Drug Surveillance Institute-INVIMA authorised this biopharmaceutical to enter the market through the approval route normally used for generic small molecule drugs [37,39]. In 2019, a retrospective study was carried out evaluating the effectiveness and safety of multiple biotherapeutics in RA, among which were Etanar and innovative etanercept with 92 and 81 patients, respectively [17]. The opinion of experts on the subject is pending, to determine if this is sufficient to consider Etanar a biosimilar. Another study on Etanar was carried out in a cohort of Colombian patients with RA, reporting a rate of 14 AE for every 100 people/year [142,193]. In addition, Etanar was compared with adalimumab and infliximab in an observational study conducted in Colombia of 158 patients, where Etanar was found to have fewer AE, with a statistically significant difference [193].

In China, this biotherapeutic has been marketed for a decade under the name Yisaipu, by Shanghai CP Goujian Pharmaceutical Co, but to date there are no published studies on AE presented in this country. Shangi CP produces this medicine to be marketed in India under the name of Etacept by the company Cipla Ltd. Etacept and its biosimilar Intacept have been assessed in a study carried out in India where four and two suspensions of treatment were reported, respectively, due to an AE [186]. Until a few years ago, Mexico commercialised a copy attempt called Etart.

Another ‘intended copy’ is Infinitam, which has an unpublished study, where this biosimilar + methotrexate is compared with etanercept (innovative) + methotrexate, but this study was very confusing and has the limitation that it was not performed head-to-head [39,42]. In Mexico, its marketing record expired in October 2017 and, when consulting the regulatory entity Federal Commission for Protection against Sanitary Risk-COFEPRIS, an active record does not appear [37,194]. However, when searching online you can still buy it at various pharmacies in this country [195,196,197].

Kikuzumab, an ‘intended copy’ of rituximab, was withdrawn from the Mexican market on March 28 2014, in the absence of clinical studies showing its safety and efficacy. It was also associated with an alert issued by the Mexico PV program in 2012, where they warned about anaphylactic reactions when switching from the innovative biopharmaceutical to kikuzumab or vice versa [37,42]. For Riditux, there are no clinical studies, but it is also striking that investigations show significant differences in the physicochemical properties compared to the reference biotherapeutic [42].

In Colombia and Mexico in 2014, a study was carried out with 219 patients with RD (AS, SLE, and RA), where Infinitam/Etanar and kikuzumab were compared, presenting AE grade 1–2 in 83.1% and grade 3–4 in 16.9%. However, it is striking that the ADR presented in such a short period of time, including on the day of exposure [198].

### 5.4. Adverse Events Reported with a Change in a Biological

The literature reports that, in addition to the AE reported with innovative biologicals, biosimilars, and copy attempts, AE can also present with a change from the innovative biological to a biosimilar or vice versa.

In Southampton, UK, 56 patients with RA, PsA, or AS were analysed, and four patients with AE were found after using the infliximab biosimilar Inflectram. One patient presented with myalgia and another with generalised pain after the administration of two infusions; the first patient was switched to ustekinumab and the second returned to the innovative biotherapeutic. A third patient presented with osteomyelitis prior to the change and a fourth patient presented with dizziness, low concentration, and labile pressure, also before the biotherapeutic change. In the latter, it was determined that there was no relationship with the biological [199].

In Japan, AE were studied in people with RA treated with etanercept, finding 265 AE (29.2%) in patients with previous infliximab treatment and 2159 AE (34.9%) in patients never treated with this biopharmaceutical, finding a statistically significant difference for any type of AE as well as for serious ADR [176]. Therefore, in addition to the current treatment, all treatments received previously should be considered.

## 6. Conclusions

It is clear, once again, that PV processes for biotherapeutics drugs and the constant generation of information on their safety profile have allowed the detection and prevention of some AE. In this article, we tried to consolidate the information on the AE of different biotherapeutics in patients with RD, documented in phase IV studies. The exercise of PV must be maintained constantly, due to the fact that biologic drugs are in a constant state of change from the initial moment of their commercialization (innovative product), as well as the progressive inclusion of biosimilars once patent protection times are met.

## Figures and Tables

**Table 1:** MeSH and Decs used in electronic database.

Term	MeSH	Supplementary Concept	Decs
1. Rheumatic Diseases	Spondylitis, Ankylosing		Spondylitis, Ankylosing
	Reiter Syndrome		
	Spondylarthropathies		Spondylarthropathies
	Spondylarthritis		Spondylarthritis
	Arthritis, Psoriatic		Arthritis, Psoriatic
	Arthritis, Reactive		Arthritis, Reactive
	Lupus Erythematosus, Systemic		Lupus Erythematosus, Systemic
	Arthritis, Juvenile		Arthritis, Juvenile
	Arthritis, Rheumatoid		Arthritis, Rheumatoid
	Rheumatic Diseases		Rheumatic Diseases
	Arthritis		Arthritis
2. Biotherapeutics	Biological Products	tocilizumab SB2 infliximab golimumab	Biological Products
			Tocilizumab
			Golimumab
	Biosimilar Pharmaceuticals		Biosimilar Pharmaceuticals
	Rituximab		Rituximab
	Infliximab		Infliximab
	Etanercept		Etanercept
	Certolizumab Pegol		Certolizumab Pegol
	Interleukin 1 Receptor Antagonist Protein		Interleukin 1 Receptor Antagonist Protein
	Adalimumab		Adalimumab
	Abatacept		Abatacept
3. Postmarketing Studies	Product Surveillance, Postmarketing		Product Surveillance, Postmarketing
	Adverse Drug Reaction Reporting Systems		Adverse Drug Reaction Reporting Systems

**Table 2:** Changes authorised by European public assessment report (EPAR) in the production of monoclonal antibodies (mAbs) for rheumatic diseases [67].

Active Substance	Risk			Total
	High	Moderate	Low	
Rituximab	1	15	7	23
Infliximab	3	34	13	50
Adalimumab	3	17	8	28
Certolizumab pegol	0	13	2	15
Golimumab	2	8	3	13
Canakinumab	0	5	1	6
Ustekinumab	0	6	3	9
Tocilizumab	1	4	2	7

**Table 3:** Principal adverse effects (AE) in rheumatic diseases (RDs) associated with biological drug treatment.

RD	ADVERSE EVENTS		
**AS**	Palmoplantar psoriasis	Injection site reactions	Cutaneous adenoid-cystic carcinoma
	Serious menstrual bleeding	Crohn’s disease	Demyelinating cervical spinal cord
	Hodgkin’s lymphoma	Multiple sclerosis	Non-Hodgkin’s lymphoma
	Proximal femur chondroblast	Ulcerative colitis	Atypical infectious mononucleosis
	Dermatitis herpetiformis	Drug induced lupus	Psoriasis/Psoriasis vulgaris
	Palmar pustulosis	Pulmonary and extrapulmonary TB	Pneumocystis jiroveci (carinii) Pneumonia
	Peripheral neuropathy	Serious infections	Hydrocephalus infant
	Heart failure	Sarcoidosis	Neutropenia
**JIA**	Erythema elevatum diutinum	Crohn’s disease	Encephalopathy
	Osteosarcoma	Multiple sclerosis	Increase in thymus
	Systemic lupus syndrome	Heart failure	Delayed maculopapular urticarial rash
	Abdominal pain	Abscess	Acne
	Allergic reactions	Alopecia	Antinuclear antibodies
	Arthralgia	Arthritis disease flare	Bacterial infection
	Blood and lymphatic system disorders	Cardiac disorders	Cellulitis
	Chickenpox	Colitis	Concentration disorder
	Crohn’s disease	Death	Dermatitis herpetiformis
	Diarrhoea	Anaphylactoid reaction	Vascular disorders
	Pneumonia	Urticaria	Uveitis
	Epistaxis	Epstein–Barr virus infection	Extrapulmonary TB
	Eye disorders	Fever	Gastrointestinal disorders
	Gastroenteritis	Haematophagic histiocytosis	Inflammatory bowel disease
	Headache	Haemolytic anaemia	Hepatobiliary disorders
	Herpes virus infection	Infusion reactions	Injection site reactions
	Lymphadenopathy	Osteoporosis	Otitis
	Pharyngitis	Pruritus	Pyelonephritis
	Sarcoidosis	Seizures	Sepsis
	Upper respiratory tract infection	Pulmonary TB	Ulcerative colitis
	Uveitis		
**PsA**	Endometrial cancer	Crohn’s disease	Heart failure
	Multiple sclerosis	Myiasis	Ulcerative colitis
	Demyelinating lesions	Pulmonary TB	Pneumocystis jiroveci (carinii) Pneumonia
	Arthralgia	Cough	Diarrhoea
	Headache	Hypertension	Injection site reaction
	Influenza-like illness	Nasopharyngitis	Pharyngolaryngeal pain
	Sinusitis	Neutropenia	Upper respiratory tract infection
	Serious infections		
**RA**	Abscess	Acute osteomyelitis	Bacterial peritonitis
	Borrelia infection	Bronchitis	Cellulitis
	Conjunctivitis	Dental/periodontal infection	Endometritis
	Erysipelas	Oesophageal candidiasis	Fungal skin infection
	Furuncle/folliculitis	Gastrointestinal infection	Herpes viral infections
	Infective arthritis	Influenza-like illness	Lung abscess
	Oral mycosis	Otitis media	Parapharyngeal abscess
	Paronychia	Pleural infection	Pneumocystis jiroveci (carinii) Pneumonia
	Pneumonia	Pulmonary TB	Pyelonephritis
	Sepsis	Sialadenitis	Thyroid gland abscess
	Tonsillitis	Upper respiratory tract infection	Vaginal mycosis/Candidiasis
	Wound infection	Diastolic dysfunction/Pulmonary hypertension	Angioedema
	Bacterial dermohypodermitis	Benign meningioma	Bilateral anterior toxic optic neuropathy
	Brucellosis	Bullous pemphigoid	Crohn’s disease
	Cutaneous squamous-cell carcinoma	Disseminated histoplasmosis	Distal Acquired Demyelinating Symmetric
	Drug induced lupus	Guillain–Barré	Heart failure
	Histoplasmosis	Leukoencephalopathy	Lymphomatoid Papulosis
	Melanoma	Multiple sclerosis	Myelitis and optic neuritis
	Necrotizing crescentic glomerulonephritis	Pancreatitis	Peripheral neuropathy
	Pleural TB	Psoriasiform dermatitis	Psoriasis
	Recurrent septic arthritis	Sarcoidosis	Schizophrenia-like disorder
	Squamous cell carcinoma of tongue	Breast cancer	Bone and joint infections
	Gastrointestinal infections	Colorectal cancer	Genitourinary tract infections
	Lung cancer	Lymphoma	Myeloma
	Severe COPD exacerbation	Serious infections	Vasculitis-like event
	Angina pectoris	Asystole	Benign gastrointestinal neoplasm
	Benign respiratory tract neoplasm	Encephalitis	Myocardial infarction
	Cerebral infarction	Facial paresis	Ovarian cancer
	Cervical cancer	Headache	Pancytopenia
	Coronary artery disorder	Myelodysplastic syndrome	Primary liver cancer
	Pulmonary oedema	Rectal cancer	Subarachnoid haemorrhage
	Venous thrombosis	Ear and labyrinth alterations	Endocrine disorders
	Metabolic and nutritional disorders	Listeriosis	Eye disorders
	Gastrointestinal perforation	Death	Rash or eczema
	Neutropenia	Congenital heart defect	Reversible Cerebral Vasoconstriction
**Sp**	Chronic inflammatory demyelinating polyneuropathy	Guillain–Barré syndrome	Motor neuropathy
	Multiple sclerosis	Myelitis	Optic neuritis
**SLE**	Progressive Multifocal Leukoencephalopathy		

RA: rheumatoid arthritis, JIA: juvenile idiopathic arthritis, PsA: psoriatic arthritis, AS: ankylosing spondylitis, Sp: Spondyloarthropaties, SLE: systemic lupus erythematosus, TB: tuberculosis.

## Supplementary Materials

The following are available online at https://www.mdpi.com/2227-9059/8/9/303/s1, Table S1: Approved biotherapeutics for rheumatic diseases, Table S2: Case series studies and case reports, Table S3: Adverse events in rheumatic diseases presented in cross-sectional studies, Table S4: Adverse biotherapeutic events in rheumatic diseases presented in case-control studies, Table S5: Adverse biotherapeutic events in rheumatic diseases presented in cohort studies (presented by percentages), Table S6: Adverse biotherapeutic events in rheumatic diseases presented in cases by 1000 patients/year.
