# Supplementary material for: Pharmacovigilance of Biopharmaceuticals in Rheumatic Diseases, Adverse Events, Evolution, and Perspective: An Overview
Source: Biomedicines. 2020 Aug 23;8(9):303. doi: 10.3390/biomedicines8090303 (PMC7555940; doi:10.3390/biomedicines8090303)
Supplement: Supplementary file 1 [file biomedicines-08-00303-s001.zip › Tablas Suplementarias/Table S2_Case reports.docx]

**Table S2. Case series studies and case reports**

| **Active principle** | **Disease** | **Biotherapeutic** | **Adverse events** | **Cases** | **Source of information** | **Country** | **Date** | **Ref** |
| --- | --- | --- | --- | --- | --- | --- | --- | --- |
| Abatacept | RA | Orencia | Squamous cell carcinoma of tongue | 1 | NS | Italy | 2014 | 75 |
| Adalimumab | AS | NS | Palmoplantar psoriasis | 1 | NS | Turkey | 2013 | 86 |
| Adalimumab | AS | NS | Serious menstrual bleeding | 2 | NS | Turkey | 2013 | 86 |
| Adalimumab | JIA | NS | Erythema elevatum diutinum | 1 | Hospital Israelita Albert Einstein and Research Institut | Brazil | 2008 | 79 |
| Adalimumab | PsA | NS | Endometrial cancer | 1 | NS | Turkey | 2013 | 86 |
| Adalimumab | RA | NS | Bullous pemhigoid | 1 | NS | Turkey | 2013 | 86 |
| Adalimumab | RA | NS | Distal Acquired Demyelinating Symmetric | 1 | NS | Ireland | 2015 | 91 |
| Adalimumab | RA | NS | Guillain Barre | 1 |  | Spain | 2013 | 69 |
| Adalimumab | RA | NS | Guillain Barre | 1 | NS | Spain | 2013 | 69 |
| Adalimumab | RA | NS | Leukoencephalopathy | 1 | NS | South Korea | 2012 | 96 |
| Adalimumab | RA | NS | Lymphomatoid Papulosis | 1 | NS | South Korea | 2012 | 93 |
| Adalimumab | RA | NS | Multiple sclerosis | 3 | NS | Inernational | 2013 | 90 |
| Adalimumab | RA | NS | Pleural TB | 1 | NS | Turkey | 2013 | 86 |
| Adalimumab | RA | NS | Pneumocystis jiroveci (carinii) Pneumonia | 1 | NS | Turkey | 2013 | 86 |
| Adalimumab | RA | NS | Sarcoidosis | 1 | NS | France | 2011 | 82 |
| Adalimumab | RA | NS | Sarcoidosis | 1 | NS | France | 2015 | 97 |
| Adalimumab | RD | NS | Multiple sclerosis | 2 | NS | Denmark | 2014 | 99 |
| Adalimumab | SU | NS | Sarcoidosis | 1 | NS | France | 2015 | 97 |
| Certolizumab pegol | AS | NS | Injection site reactions | 1 | NS | Turkey | 2013 | 86 |
| Etanercept | AS | NS | Crohn's disease | 1 | NS | Turkey | 2013 | 86 |
| Etanercept | AS | NS | Crohn's disease | 11 | BWH patient database and the FDA Adverse Event Reporting System y FAERS | United States | 2016 | 92 |
| Etanercept | AS | NS | Cutaneous adenoid-cystic carcinoma | 1 | NS | Turkey | 2013 | 86 |
| Etanercept | AS | NS | Demyelinating cervical spinal cord | 1 | NS | Turkey | 2013 | 86 |
| Etanercept | AS | NS | Hodgkin’s lymphoma | 1 | NS | Turkey | 2013 | 86 |
| Etanercept | AS | NS | Multiple sclerosis | 1 | NS | Inernational | 2013 | 90 |
| Etanercept | AS | NS | Multiple sclerosis | 1 | NS | Inernational | 2013 | 90 |
| Etanercept | AS | NS | Non Hodgkin’s lymphoma | 1 | NS | Turkey | 2013 | 86 |
| Etanercept | AS | NS | Proximal femur chondroblast | 1 | NS | China | 2018 | 73 |
| Etanercept | AS | NS | Ulcerative colitis | 3 | Brigham and Women’s (BWH) patient database and the FDA Adverse Event Reporting System y FAERS Food and Drug Administration Adverse Event Reporting System | United States | 2016 | 92 |
| Etanercept | JIA | NS | Crohn's disease | 11 | Brigham and Women’s (BWH) patient database and the FDA Adverse Event Reporting System y FAERS Food and Drug Administration Adverse Event Reporting System | United States | 2016 | 92 |
| Etanercept | JIA | NS | Encephalopathy | 1 | NS | Turkey | 2013 | 86 |
| Etanercept | JIA | NS | Increase of thymus and constitutional symptoms | 1 | NS | Turkey | 2013 | 86 |
| Etanercept | JIA | NS | Multiple sclerosis | 1 | NS | Inernational | 2013 | 90 |
| Etanercept | JIA | NS | Ostesarcoma en femur o humero | 2 | NS | China | 2018 | 73 |
| Etanercept | PsA | NS | Crohn's disease | 7 | Brigham and Women’s (BWH) patient database and the FDA Adverse Event Reporting System y FAERS Food and Drug Administration Adverse Event Reporting System | United States | 2016 | 92 |
| Etanercept | PsA | NS | Heart failure | 1 | FDA’s MedWatch program | United States | 2003 | 87 |
| Etanercept | PsA | NS | Multiple sclerosis | 1 | NS | Inernational | 2013 | 90 |
| Etanercept | PsA | NS | Multiple sclerosis | 3 | NS | Inernational | 2013 | 90 |
| Etanercept | PsA | NS | Myiasis | 1 | NS | Turkey | 2013 | 84 |
| Etanercept | PsA | NS | Ulcerative colitis | 5 | Brigham and Women’s (BWH) patient database and the FDA Adverse Event Reporting System y FAERS Food and Drug Administration Adverse Event Reporting System | United States | 2016 | 92 |
| Etanercept | RA | NS | Angioedema | 1 | NS | Turkey | 2013 | 86 |
| Etanercept | RA | NS | Benign meningioma | 1 | NS | Turkey | 2013 | 86 |
| Etanercept | RA | NS | Bilateral septic hip arthritis due to Staphylococcus aureus | 1 | Hadassah-Hebrew University School of Medicine | Israel | 2003 | 70 |
| Etanercept | RA | NS | cellulitis causing tissue defect | 1 | NS | Turkey | 2013 | 86 |
| Etanercept | RA | NS | Crohn's disease | 9 | Brigham and Women’s (BWH) patient database and the FDA Adverse Event Reporting System y FAERS Food and Drug Administration Adverse Event Reporting System | United States | 2016 | 92 |
| Etanercept | RA | NS | Cutaneous squamous-cell carcinoma | 7 | National Naval Medical Center | United States | 2001 | 98 |
| Etanercept | RA | NS | Disseminated histoplasmosis | 1 | Johns Hopkins Hospital | United States | 2004 | 78 |
| Etanercept | RA | NS | Drug induced lupus | 1 | NS | Turkey | 2013 | 86 |
| Etanercept | RA | NS | Heart failure | 3 | FDA’s MedWatch program | United States | 2003 | 87 |
| Etanercept | RA | NS | Histoplasmosis | 1 | FDA Adverse Event Reporting System (AERS) | United States | 2002 | 88 |
| Etanercept | RA | NS | Multiple sclerosis | 3 | NS | Inernational | 2013 | 90 |
| Etanercept | RA | NS | Myelitis and optic neuritis | 1 | Tokyo Medical And Dental University, Medical Hospital | Japan | 2014 | 102 |
| Etanercept | RA | NS | Necrotizing crescentic glomerulonephritis | 5 | NS | Inernational | 2010 | 84 |
| Etanercept | RA | NS | Psoriasiform dermatitis | 1 | NS | Turkey | 2013 | 86 |
| Etanercept | RA | NS | Psoriasis | 1 | NS | Turkey | 2013 | 86 |
| Etanercept | RA | NS | Pulmonary TB | 2 | NS | Turkey | 2013 | 86 |
| Etanercept | RA | NS | Recurrent septic arthritis | 1 | NS | Turkey | 2013 | 86 |
| Etanercept | RA | NS | Sarcoidosis | 2 | NS | France | 2011 | 82 |
| Etanercept | RA | NS | Schizophrenia-like disorder | 1 | NS | UK | 2014 | 71 |
| Etanercept | RA | Enbrel | Systemic lupus syndrome | 1 | Georges Pompidou Teaching Hospital | France | 2003 | 74 |
| Etanercept | RA and SLE | NS | Progressive Multifocal Leukoencephalopathy | 1 | Clinica Mayo | United States | 2012 | 80 |
| Etanercept | RA, AS and PsA | NS | Heart failure | 21 | U.S. National Data Bank for Rheumatic Diseases, | United States | 2013 | 86 |
| Etanercept | RD | NS | Multiple sclerosis | 1 | NS | Denmark | 2014 | 99 |
| Etanercept | SU | NS | Atypical carcinoid tumor of the thymus with ectopic ACTH production | 1 | NS | Turkey | 2013 | 86 |
| Infliximab | AS | NS | Atypical infectious mononucleosis | 1 | NS | Turkey | 2013 | 86 |
| Infliximab | AS | NS | Dermatitis herpetiformis | 1 | NS | Turkey | 2013 | 86 |
| Infliximab | AS | NS | Drug induced lupus | 1 | NS | Turkey | 2013 | 86 |
| Infliximab | AS | NS | Extrapulmonary TB | 2 | NS | Turkey | 2013 | 86 |
| Infliximab | AS | NS | No Hodgkin’s lymphoma | 1 | NS | Turkey | 2013 | 86 |
| Infliximab | AS | NS | Palmar pustulosis | 1 | NS | Turkey | 2013 | 86 |
| Infliximab | AS | NS | Palmoplantar psoriasis | 2 | NS | Turkey | 2013 | 86 |
| Infliximab | AS | NS | Pleural TB | 1 | NS | Turkey | 2013 | 86 |
| Infliximab | AS | NS | Pneumocystis jiroveci (carinii) Pneumonia | 2 | Adverse Event Reporting System | United States | 2007 | 85 |
| Infliximab | AS | NS | Psoriasis | 1 | NS | Turkey | 2013 | 86 |
| Infliximab | AS | NS | Psoriasis vulgaris | 1 | NS | Turkey | 2013 | 86 |
| Infliximab | AS | NS | Pulmonary and extrapulmonary TB | 1 | NS | Turkey | 2013 | 86 |
| Infliximab | AS | NS | TB | 5 | FDA’s MedWatch program | United States | 2007 | 95 |
| Infliximab | JIA | NS | Delayed maculopapular urticarial rash | 2 | NS | Turkey | 2013 | 86 |
| Infliximab | JIA | NS | Heart failure | 1 | FDA’s MedWatch program | United States | 2003 | 87 |
| Infliximab | JIA | NS | Systemic lupus syndrome | 1 | Georges Pompidou Teaching Hospital | France | 2003 | 74 |
| Infliximab | PsA | NS | Demyelinating lesions | 1 | NS | Greek | 2014 | 83 |
| Infliximab | PsA | NS | Pneumocystis jiroveci (carinii) Pneumonia | 1 | Adverse Event Reporting System | United States | 2007 | 85 |
| Infliximab | PsA | NS | TB | 3 | FDA’s MedWatch program | United States | 2007 | 95 |
| Infliximab | RA | NS | Acceleration of left-ventricular diastolic dysfunction and pulmonary hypertension | 1 | NS | Turkey | 2013 | 86 |
| Infliximab | RA | NS | Brucellosis | 1 | NS | Turkey | 2013 | 86 |
| Infliximab | RA | NS | Heart failure | 2 | FDA’s MedWatch program | United States | 2003 | 87 |
| Infliximab | RA | NS | Histoplasmosis | 5 | FDA Adverse Event Reporting System (AERS) | United States | 2002 | 88 |
| Infliximab | RA | NS | Multiple sclerosis | 3 | NS | Inernational | 2013 | 90 |
| Infliximab | RA | NS | Optic neuritis | 1 | NS | Turkey | 2013 | 86 |
| Infliximab | RA | NS | Peripheral neuropathy | 1 | NS | Mediterranean | 2014 | 76 |
| Infliximab | RA | NS | Pneumocystis jiroveci (carinii) Pneumonia | 49 | Adverse Event Reporting System | United States | 2007 | 85 |
| Infliximab | RA | NS | TB | 5 | FDA’s MedWatch program | United States | 2007 | 95 |
| Infliximab | RD | NS | Multiple sclerosis | 2 | NS | Denmark | 2014 | 99 |
| Infliximab | RD | NS | Peripheral neuropathy | 1 | NS | Denmark | 2014 | 99 |
| Rituximab | RA | NS | Bilateral anterior toxic optic neuropathy | 3 | Netherlands Pharmacovigilance Centre Lareb | Netherlands | 2003 | 100 |
| Rituximab | RA | NS | Melanoma | 5 | EudraVigilance | Germany, France, Italy, United States | 2013 | 94 |
| Rituximab | SLE | NS | Progressive Multifocal Leukoencephalopathy | 2 | Hospital de Santo António, Centro Hospitalar do Porto | Portugal | 2012 | 72 |
| Rituximab | SLE | NS | Progressive Multifocal Leukoencephalopathy | 1 | Cameron Hospital | UK | 2007 | 81 |
| Rituximab | SpA | NS | Melanoma | 1 | NS | UK | 2013 | 94 |
| Tocilizumab | RA | NS | Bacterial dermohypodermitis | 1 | NS | France | 2017 | 101 |
| Tocilizumab | RA | RoActemra | Pancreatitis | 1 | NS | Germany | 2016 | 77 |
| Tocilizumab | SeA | NS | Reversible Cerebral Vasoconstriction | 1 | NS | Spain | 2019 | 89 |
