# Supplementary material for: Pharmacovigilance of Biopharmaceuticals in Rheumatic Diseases, Adverse Events, Evolution, and Perspective: An Overview
Source: Biomedicines. 2020 Aug 23;8(9):303. doi: 10.3390/biomedicines8090303 (PMC7555940; doi:10.3390/biomedicines8090303)
Supplement: Supplementary file 1 [file biomedicines-08-00303-s001.zip › Tablas Suplementarias/Table S3_Cross sectional.docx]

**Table S3. Adverse events in rheumatic diseases presented in cross-sectional studies**

| **Active principle** | **Disease** | **Biotherapeutic** | **Adverse events** | **Cases** | **Cases %** | **Source of information** | **n** | **Country** | **Reference** |
| --- | --- | --- | --- | --- | --- | --- | --- | --- | --- |
| Abatacept | Rheumatoid arthritis | Orencia | Rash or eczema | 2 | 0,03 | Rheumatoid Arthritis Center | 6793* | Colombia | 103 |
| Certolizumab pegol | Rheumatoid arthritis | Cimzia | Rash or eczema | 5 | 0,07 | Rheumatoid Arthritis Center | 6793* | Colombia | 103 |
| Etanercept | Rheumatoid arthritis | NS | Rash or eczema | 2 | 0,03 | Rheumatoid Arthritis Center | 6793* | Colombia | 103 |
| Golimumab | Rheumatoid arthritis | NS | Rash or eczema | 1 | 0,01 | Rheumatoid Arthritis Center | 6793* | Colombia | 103 |
| Infliximab | Rheumatoid arthritis | NS | Rash or eczema | 1 | 0,01 | Rheumatoid Arthritis Center | 6793* | Colombia | 103 |
| Tocilizumab | Rheumatoid arthritis | NS | Rash or eczema | 1 | 0,01 | Rheumatoid Arthritis Center | 6793* | Colombia | 103 |
