# Supplementary material for: Pharmacovigilance of Biopharmaceuticals in Rheumatic Diseases, Adverse Events, Evolution, and Perspective: An Overview
Source: Biomedicines. 2020 Aug 23;8(9):303. doi: 10.3390/biomedicines8090303 (PMC7555940; doi:10.3390/biomedicines8090303)
Supplement: Supplementary file 1 [file biomedicines-08-00303-s001.zip › Tablas Suplementarias/Table S4_Case-control.docx]

**Table S4. Adverse biotherapeutic events in rheumatic diseases presented in case-control studies**

| **Active principle** | **Disease** | **Biotherapeutic** | **Adverse events** | **Cases** | **Source of information** | **Directionality** | **n** | **Country** | **Date** | **Reference** |
| --- | --- | --- | --- | --- | --- | --- | --- | --- | --- | --- |
| Adalimumab | RA | NS | Pneumocystis jiroveci (carinii) Pneumonia | 17 | 16 Hospitals | Retrospective | NS | Japan | 2013 | 107 |
| Adalimumab | RA | NS | TB | 9 |  |  | 7755 | Japan | 2016 | 106 |
| Etanercept | RA | NS | Abscess | 3 | RABBIT | Prospective | 512 | Germany | 2005 | 162 |
| Etanercept | RA | NS | Acute osteomyelitis | 0 | RABBIT | Prospective | 512 | Germany | 2005 | 162 |
| Etanercept | RA | NS | Bacterial peritonitis | 0 | RABBIT | Prospective | 512 | Germany | 2005 | 162 |
| Etanercept | RA | NS | Borrelia infection | 0 | RABBIT | Prospective | 512 | Germany | 2005 | 162 |
| Etanercept | RA | NS | Bronchitis | 11 | RABBIT | Prospective | 512 | Germany | 2005 | 162 |
| Etanercept | RA | NS | Cellulitis | 3 | RABBIT | Prospective | 512 | Germany | 2005 | 162 |
| Etanercept | RA | NS | Conjunctivitis | 0 | RABBIT | Prospective | 512 | Germany | 2005 | 162 |
| Etanercept | RA | NS | Dental/periodontal infection | 0 | RABBIT | Prospective | 512 | Germany | 2005 | 162 |
| Etanercept | RA | NS | Endometritis | 0 | RABBIT | Prospective | 512 | Germany | 2005 | 162 |
| Etanercept | RA | NS | Erysipelas | 4 | RABBIT | Prospective | 512 | Germany | 2005 | 162 |
| Etanercept | RA | NS | Esophageal candidiasis | 0 | RABBIT | Prospective | 512 | Germany | 2005 | 162 |
| Etanercept | RA | NS | Fungal skin infection | 2 | RABBIT | Prospective | 512 | Germany | 2005 | 162 |
| Etanercept | RA | NS | Furuncle/folliculitis | 4 | RABBIT | Prospective | 512 | Germany | 2005 | 162 |
| Etanercept | RA | NS | Gastrointestinal infection | 3 | RABBIT | Prospective | 512 | Germany | 2005 | 162 |
| Etanercept | RA | NS | Herpes simplex | 4 | RABBIT | Prospective | 512 | Germany | 2005 | 162 |
| Etanercept | RA | NS | Herpes zoster | 5 | RABBIT | Prospective | 512 | Germany | 2005 | 162 |
| Etanercept | RA | NS | Infective arthritis | 5 | RABBIT | Prospective | 512 | Germany | 2005 | 162 |
| Etanercept | RA | NS | Infective bursitis | 0 | RABBIT | Prospective | 512 | Germany | 2005 | 162 |
| Etanercept | RA | NS | Influenza‐like illness | 13 | RABBIT | Prospective | 512 | Germany | 2005 | 162 |
| Etanercept | RA | NS | Lung abscess | 1 | RABBIT | Prospective | 512 | Germany | 2005 | 162 |
| Etanercept | RA | NS | Oral mycosis | 3 | RABBIT | Prospective | 512 | Germany | 2005 | 162 |
| Etanercept | RA | NS | Other bacterial skin infection | 1 | RABBIT | Prospective | 512 | Germany | 2005 | 162 |
| Etanercept | RA | NS | Other respiratory tract infection | 0 | RABBIT | Prospective | 512 | Germany | 2005 | 162 |
| Etanercept | RA | NS | Otitis media | 1 | RABBIT | Prospective | 512 | Germany | 2005 | 162 |
| Etanercept | RA | NS | Parapharyngeal abscess | 0 | RABBIT | Prospective | 512 | Germany | 2005 | 162 |
| Etanercept | RA | NS | Paronychia | 3 | RABBIT | Prospective | 512 | Germany | 2005 | 162 |
| Etanercept | RA | NS | Pleural infection | 1 | RABBIT | Prospective | 512 | Germany | 2005 | 162 |
| Etanercept | RA | NS | Pneumocystis jiroveci (carinii) Pneumonia | 15 | 21 Hospitals | Retrospective | NS | Japan | 2012 | 105 |
| Etanercept | RA | NS | Pneumonia | 6 | RABBIT | Prospective | 512 | Germany | 2005 | 162 |
| Etanercept | RA | NS | Pulmonary TB | 0 | RABBIT | Prospective | 512 | Germany | 2005 | 162 |
| Etanercept | RA | NS | Pyelonephritis | 1 | RABBIT | Prospective | 512 | Germany | 2005 | 162 |
| Etanercept | RA | NS | Sepsis/urosepsis | 3 | RABBIT | Prospective | 512 | Germany | 2005 | 162 |
| Etanercept | RA | NS | Sialadenitis | 0 | RABBIT | Prospective | 512 | Germany | 2005 | 162 |
| Etanercept | RA | NS | Thyroid gland abscess | 0 | RABBIT | Prospective | 512 | Germany | 2005 | 162 |
| Etanercept | RA | NS | Tonsillitis | 4 | RABBIT | Prospective | 512 | Germany | 2005 | 162 |
| Etanercept | RA | NS | Upper respiratory tract infection | 15 | RABBIT | Prospective | 512 | Germany | 2005 | 162 |
| Etanercept | RA | NS | Urinary tract infection | 8 | RABBIT | Prospective | 512 | Germany | 2005 | 162 |
| Infliximab | RA | NS | Abscess | 5 | RABBIT | Prospective | 346 | Germany | 2005 | 162 |
| Etanercept | RA | NS | Vaginal mycosis/candidiasis | 4 | RABBIT | Prospective | 512 | Germany | 2005 | 162 |
| Etanercept | RA | NS | Wound infection | 0 | RABBIT | Prospective | 512 | Germany | 2005 | 162 |
| Infliximab | RA | NS | Acute osteomyelitis | 0 | RABBIT | Prospective | 346 | Germany | 2005 | 162 |
| Infliximab | RA | NS | Bacterial peritonitis | 0 | RABBIT | Prospective | 346 | Germany | 2005 | 162 |
| Infliximab | RA | NS | Borrelia infection | 1 | RABBIT | Prospective | 346 | Germany | 2005 | 162 |
| Infliximab | RA | NS | Bronchitis | 7 | RABBIT | Prospective | 346 | Germany | 2005 | 162 |
| Infliximab | RA | NS | Cellulitis | 1 | RABBIT | Prospective | 346 | Germany | 2005 | 162 |
| Infliximab | RA | NS | Conjunctivitis | 0 | RABBIT | Prospective | 346 | Germany | 2005 | 162 |
| Infliximab | RA | NS | Dental/periodontal infection | 2 | RABBIT | Prospective | 346 | Germany | 2005 | 162 |
| Infliximab | RA | NS | Endometritis | 1 | RABBIT | Prospective | 346 | Germany | 2005 | 162 |
| Infliximab | RA | NS | Erysipelas | 0 | RABBIT | Prospective | 346 | Germany | 2005 | 162 |
| Infliximab | RA | NS | Esophageal candidiasis | 0 | RABBIT | Prospective | 346 | Germany | 2005 | 162 |
| Infliximab | RA | NS | Fungal skin infection | 1 | RABBIT | Prospective | 346 | Germany | 2005 | 162 |
| Infliximab | RA | NS | Furuncle/folliculitis | 2 | RABBIT | Prospective | 346 | Germany | 2005 | 162 |
| Infliximab | RA | NS | Gastrointestinal infection | 2 | RABBIT | Prospective | 346 | Germany | 2005 | 162 |
| Infliximab | RA | NS | Herpes simplex | 6 | RABBIT | Prospective | 346 | Germany | 2005 | 162 |
| Infliximab | RA | NS | Herpes zoster | 5 | RABBIT | Prospective | 346 | Germany | 2005 | 162 |
| Infliximab | RA | NS | Infective arthritis | 1 | RABBIT | Prospective | 346 | Germany | 2005 | 162 |
| Infliximab | RA | NS | Infective bursitis | 1 | RABBIT | Prospective | 346 | Germany | 2005 | 162 |
| Infliximab | RA | NS | Influenza‐like illness | 13 | RABBIT | Prospective | 346 | Germany | 2005 | 162 |
| Infliximab | RA | NS | Lung abscess | 0 | RABBIT | Prospective | 346 | Germany | 2005 | 162 |
| Infliximab | RA | NS | Oral mycosis | 2 | RABBIT | Prospective | 346 | Germany | 2005 | 162 |
| Infliximab | RA | NS | Other bacterial skin infection | 0 | RABBIT | Prospective | 346 | Germany | 2005 | 162 |
| Infliximab | RA | NS | Other respiratory tract infection | 4 | RABBIT | Prospective | 346 | Germany | 2005 | 162 |
| Infliximab | RA | NS | Otitis media | 0 | RABBIT | Prospective | 346 | Germany | 2005 | 162 |
| Infliximab | RA | NS | Parapharyngeal abscess | 1 | RABBIT | Prospective | 346 | Germany | 2005 | 162 |
| Infliximab | RA | NS | Paronychia | 3 | RABBIT | Prospective | 346 | Germany | 2005 | 162 |
| Infliximab | RA | NS | Pleural infection | 0 | RABBIT | Prospective | 346 | Germany | 2005 | 162 |
| Infliximab | RA | NS | Pneumocystis jiroveci (carinii) Pneumonia | 1 | 14 Hospitals | Retrospective | NS | Japan | 2009 | 104 |
| Infliximab | RA | NS | Pneumonia | 8 | RABBIT | Prospective | 346 | Germany | 2005 | 162 |
| Infliximab | RA | NS | Pulmonary TB | 1 | RABBIT | Prospective | 346 | Germany | 2005 | 162 |
| Infliximab | RA | NS | Pyelonephritis | 1 | RABBIT | Prospective | 346 | Germany | 2005 | 162 |
| Infliximab | RA | NS | Sepsis/urosepsis | 0 | RABBIT | Prospective | 346 | Germany | 2005 | 162 |
| Infliximab | RA | NS | Sialadenitis | 1 | RABBIT | Prospective | 346 | Germany | 2005 | 162 |
| Infliximab | RA | NS | Thyroid gland abscess | 1 | RABBIT | Prospective | 346 | Germany | 2005 | 162 |
| Infliximab | RA | NS | Tonsillitis | 0 | RABBIT | Prospective | 346 | Germany | 2005 | 162 |
| Infliximab | RA | NS | Upper respiratory tract infection | 17 | RABBIT | Prospective | 346 | Germany | 2005 | 162 |
| Infliximab | RA | NS | Urinary tract infection | 3 | RABBIT | Prospective | 346 | Germany | 2005 | 162 |
| Infliximab | RA | NS | Vaginal mycosis/candidiasis | 0 | RABBIT | Prospective | 346 | Germany | 2005 | 162 |
| Infliximab | RA | NS | Wound infection | 1 | RABBIT | Prospective | 346 | Germany | 2005 | 162 |
