# Supplementary material for: Pharmacovigilance of Biopharmaceuticals in Rheumatic Diseases, Adverse Events, Evolution, and Perspective: An Overview
Source: Biomedicines. 2020 Aug 23;8(9):303. doi: 10.3390/biomedicines8090303 (PMC7555940; doi:10.3390/biomedicines8090303)
Supplement: Supplementary file 1 [file biomedicines-08-00303-s001.zip › Tablas Suplementarias/Table S5_Cohort %.docx]

**Table S5. Adverse biotherapeutic events in rheumatic diseases presented in cohort studies (presented by percentages)**

| **Active principle** | **Disease** | **Biotherapeutic** | **Adverse events** | **Cases** | **Cases %** | **Source of information** | **Directionality** | **n** | **Country** | **Date** | **Reference** |
| --- | --- | --- | --- | --- | --- | --- | --- | --- | --- | --- | --- |
| Abatacept | JIA | NS | Arthralgia | 0 | 0 | 43 International Pediatric Rheumatology Testing Organizations / Pediatric Rheumatology Collaborative Study Group and Pediatric Rheumatology Centers | Prospective | 58 | North America, Latin America and Europe | 2010 | 117 |
| Abatacept | JIA | NS | Arthritis disease flare | 3 | 5,2 | 43 International Pediatric Rheumatology Testing Organizations / Pediatric Rheumatology Collaborative Study Group and Pediatric Rheumatology Centers | Prospective | 58 | North America, Latin America and Europe | 2010 | 117 |
| Abatacept | JIA | NS | Cough | 7 | 12,1 | 43 International Pediatric Rheumatology Testing Organizations / Pediatric Rheumatology Collaborative Study Group and Pediatric Rheumatology Centers | Prospective | 58 | North America, Latin America and Europe | 2010 | 117 |
| Abatacept | JIA | NS | Diarrhea | 5 | 8,6 | 43 International Pediatric Rheumatology Testing Organizations / Pediatric Rheumatology Collaborative Study Group and Pediatric Rheumatology Centers | Prospective | 58 | North America, Latin America and Europe | 2010 | 117 |
| Abatacept | JIA | NS | Nasopharyngitis | 14 | 24,1 | 43 International Pediatric Rheumatology Testing Organizations / Pediatric Rheumatology Collaborative Study Group and Pediatric Rheumatology Centers | Prospective | 58 | North America, Latin America and Europe | 2010 | 117 |
| Abatacept | JIA | NS | Pharyngitis | 8 | 13,8 | 43 International Pediatric Rheumatology Testing Organizations / Pediatric Rheumatology Collaborative Study Group and Pediatric Rheumatology Centers | Prospective | 58 | North America, Latin America and Europe | 2010 | 117 |
| Abatacept | JIA | NS | Pyrexia | 7 | 12,1 | 43 International Pediatric Rheumatology Testing Organizations / Pediatric Rheumatology Collaborative Study Group and Pediatric Rheumatology Centers | Prospective | 58 | North America, Latin America and Europe | 2010 | 117 |
| Abatacept | JIA | NS | Pyrexia £ | 1 | 1,7 | 43 International Pediatric Rheumatology Testing Organizations / Pediatric Rheumatology Collaborative Study Group and Pediatric Rheumatology Centers | Prospective | 58 | North America, Latin America and Europe | 2010 | 117 |
| Abatacept | JIA | NS | Sickness | 5 | 8,6 | 43 International Pediatric Rheumatology Testing Organizations / Pediatric Rheumatology Collaborative Study Group and Pediatric Rheumatology Centers | Prospective | 58 | North America, Latin America and Europe | 2010 | 117 |
| Abatacept | JIA | NS | Upper respiratory tract infection | 6 | 10,3 | 43 International Pediatric Rheumatology Testing Organizations / Pediatric Rheumatology Collaborative Study Group and Pediatric Rheumatology Centers | Prospective | 58 | North America, Latin America and Europe | 2010 | 117 |
| Abatacept | JIA | NS | Vomiting | 9 | 15,5 | 43 International Pediatric Rheumatology Testing Organizations / Pediatric Rheumatology Collaborative Study Group and Pediatric Rheumatology Centers | Prospective | 58 | North America, Latin America and Europe | 2010 | 117 |
| Abatacept | JIA | NS | Vomiting | 1 | 1,7 | 43 International Pediatric Rheumatology Testing Organizations / Pediatric Rheumatology Collaborative Study Group and Pediatric Rheumatology Centers | Prospective | 58 | North America, Latin America and Europe | 2010 | 117 |
| Abatacept | RA | Orencia | Administration site reactions | 14 | 2,75 | multicentric | Prospective | 509 | Japan | 2018 | 115 |
| Abatacept | RA | Orencia | Bronchitis | 35 | 0,9 | 772 centers | Prospective | 3882 | Japan | 2016 | 114 |
| Abatacept | RA | Orencia | Cutaneous infection | 10 | 0,98 | French Registry of Orencia and AR Registry | Prospective | 1017 | France | 2016 | 116 |
| Abatacept | RA | Orencia | General disorders and administration site conditions | 20 | 3,93 | Multicentric | Prospective | 509 | Japan | 2018 | 115 |
| Abatacept | RA | Orencia | Genitourinary infection | 16 | 1,57 | French Registry of Orencia and AR Registry | Prospective | 1017 | France | 2016 | 116 |
| Abatacept | RA | Orencia | Herpes zoster | 39 | 1 | 772 centers | Prospective | 3882 | Japan | 2016 | 114 |
| Abatacept | RA | Orencia | Herpes zoster | 3 | 0,59 | Multicentric | Prospective | 509 | Japan | 2018 | 115 |
| Abatacept | RA | Orencia | Infections and infestations | 84 | 16,5 | Multicentric | Prospective | 509 | Japan | 2018 | 115 |
| Abatacept | RA | Orencia | Interstitial lung disease | 9 | 1,77 | Multicentric | Prospective | 509 | Japan | 2018 | 115 |
| Abatacept | RA | Orencia | Liver function test abnormal | 29 | 0,75 | 772 centers | Prospective | 3882 | Japan | 2016 | 114 |
| Abatacept | RA | Orencia | Malignant neoplasm | 6 | 1,18 | Multicentric | Prospective | 509 | Japan | 2018 | 115 |
| Abatacept | RA | Orencia | Nasopharyngitis | 31 | 0,8 | 772 centers | Prospective | 3882 | Japan | 2016 | 114 |
| Abatacept | RA | Orencia | Other infections | 43 | 4,23 | French Registry of Orencia and AR Registry | Prospective | 1017 | France | 2016 | 116 |
| Abatacept | RA | Orencia | Pancytopenia | 1 | 0,2 | Multicentric | Prospective | 509 | Japan | 2018 | 115 |
| Abatacept | RA | Orencia | Pneumocystis jiroveci (carinii) Pneumonia | 0 | 0 | Multicentric | Prospective | 509 | Japan | 2018 | 115 |
| Abatacept | RA | Orencia | Post-operative infection | 2 | 0,2 | French Registry of Orencia and AR Registry | Prospective | 1017 | France | 2016 | 116 |
| Abatacept | RA | Orencia | Prosthetic joint infection | 8 | 0,79 | French Registry of Orencia and AR Registry | Prospective | 1017 | France | 2016 | 116 |
| Abatacept | RA | Orencia | Pyrexia | 24 | 0,62 | 772 centers | Prospective | 3882 | Japan | 2016 | 114 |
| Abatacept | RA | Orencia | Rash | 23 | 0,59 | 772 centers | Prospective | 3882 | Japan | 2016 | 114 |
| Abatacept | RA | Orencia | Respiratory infection | 57 | 5,6 | French Registry of Orencia and AR Registry | Prospective | 1017 | France | 2016 | 116 |
| Abatacept | RA | Orencia | Respiratory, thoracic and mediastinal disorders | 35 | 6,88 | Multicentric | Prospective | 509 | Japan | 2018 | 115 |
| Abatacept | RA | Orencia | Sepsis | 1 | 0,2 | Multicentric | Prospective | 509 | Japan | 2018 | 115 |
| Abatacept | RA | Orencia | Septicemia | 11 | 1,08 | French Registry of Orencia and AR Registry | Prospective | 1017 | France | 2016 | 116 |
| Abatacept | RA | Orencia | Skin and subcutaneous tissue disorders | 29 | 5,7 | Multicentric | Prospective | 509 | Japan | 2018 | 115 |
| Abatacept | RA | Orencia | Stomatitis | 34 | 0,88 | 772 centers | Prospective | 3882 | Japan | 2016 | 114 |
| Abatacept | RA | Orencia | TB | 0 | 0 | Multicentric | Prospective | 509 | Japan | 2018 | 115 |
| Abatacept | RA | NS | Total malignancies (excluding non-melanoma skin cancer) | NS | NS | National Data Bank for Rheumatic Diseases -  Patient questionnaire | Prospective | 10499 | United States | 2008 | 145 |
| Abatacept | RA | Orencia | Upper respiratory tract inflammation | 47 | 1,21 | 772 centers | Prospective | 3882 | Japan | 2016 | 114 |
| Adalimumab | RA | Humira | Acne | 2 | 1,85 | Rheumatoid Arthritis Reference Center | Retrospective | 108 | Colombia | 2016 | 141 |
| Adalimumab | RA | Humira | Angioedema | 4 | 3,7 | Rheumatoid Arthritis Reference Center | Retrospective | 108 | Colombia | 2016 | 141 |
| Adalimumab | RA | NS | Atypical mycobacteria | 6 | 0,08 | 1722 medical centers | Prospective | 7740 | Japan | 2014 | 139 |
| Adalimumab | RA | NS | Autoinmune disease | 11 | 0,14 | 1722 medical centers | Prospective | 7740 | Japan | 2014 | 139 |
| Adalimumab | RA | Humira | Breast mass, under study | 2 | 1,85 | Rheumatoid Arthritis Reference Center | Retrospective | 108 | Colombia | 2016 | 141 |
| Adalimumab | RA | NS | Cancer | 1 | 0,62 | 76 centers | Prospective | 162 | 15 Australasia, Europe, North and South america countries | 2013 | 122 |
| Adalimumab | RA | NS | Candidiasis | 4 | 3,7 | Rheumatoid Arthritis Reference Center | Retrospective | 108 | Colombia | 2019 | 118 |
| Adalimumab | RA | Humira | Candidiasis | 4 | 3,7 | Rheumatoid Arthritis Reference Center | Retrospective | 108 | Colombia | 2016 | 141 |
| Adalimumab | RA | NS | Cellulitis | 2 | 1,23 | 76 centers | Prospective | 162 | 15 Australasia, Europe, North and South america countries | 2013 | 122 |
| Adalimumab | RA | NS | Cellulitis | 0 | 0 | Rheumatoid Arthritis Reference Center | Retrospective | 108 | Colombia | 2019 | 118 |
| Adalimumab | RA | Humira | Cellulitis | 0 | 0 | Rheumatoid Arthritis Reference Center | Retrospective | 108 | Colombia | 2016 | 141 |
| Adalimumab | RA | Humira | Cervical cancer | 2 | 1,85 | Rheumatoid Arthritis Reference Center | Retrospective | 108 | Colombia | 2016 | 141 |
| Adalimumab | RA | Humira | Chilblains | 2 | 1,85 | Rheumatoid Arthritis Reference Center | Retrospective | 108 | Colombia | 2016 | 141 |
| Adalimumab | RA | NS | Cord demyelinating disorder | 2 | 0,03 | 1722 medical centers | Prospective | 7740 | Japan | 2014 | 139 |
| Adalimumab | RA | NS | Dermatitis | 16 | 14,81 | Rheumatoid Arthritis Reference Center | Retrospective | 108 | Colombia | 2019 | 118 |
| Adalimumab | RA | Humira | Dermatitis | 24 | 22,22 | Rheumatoid Arthritis Reference Center | Retrospective | 108 | Colombia | 2016 | 141 |
| Adalimumab | RA | NS | Dermatomycosis | 0 | 0 | Rheumatoid Arthritis Reference Center | Retrospective | 108 | Colombia | 2019 | 118 |
| Adalimumab | RA | NS | Diarrhea | 0 | 0 | Rheumatoid Arthritis Reference Center | Retrospective | 108 | Colombia | 2019 | 118 |
| Adalimumab | RA | Humira | Diarrhea | 0 | 0 | Rheumatoid Arthritis Reference Center | Retrospective | 108 | Colombia | 2016 | 141 |
| Adalimumab | RA | NS | Elevated blood pressure | 0 | 0 | Rheumatoid Arthritis Reference Center | Retrospective | 108 | Colombia | 2019 | 118 |
| Adalimumab | RA | Humira | Elevated blood pressure | 0 | 0 | Rheumatoid Arthritis Reference Center | Retrospective | 108 | Colombia | 2016 | 141 |
| Adalimumab | RA | Humira | Fatal adverse events | 102 | 1,54 | ReAct/ReAlise | Prospective | 6610 | Multicentric | 2014 | 120 |
| Adalimumab | RA | NS | Fungal infection | 8 | 0,1 | 1722 medical centers | Prospective | 7740 | Japan | 2014 | 139 |
| Adalimumab | RA | NS | General disorders and administration site conditions | 459 | 5,93 | 1722 medical centers | Prospective | 7740 | Japan | 2014 | 139 |
| Adalimumab | RA | NS | Headache | 5 | 4,63 | Rheumatoid Arthritis Reference Center | Retrospective | 108 | Colombia | 2019 | 118 |
| Adalimumab | RA | Humira | Headache | 4 | 3,7 | Rheumatoid Arthritis Reference Center | Retrospective | 108 | Colombia | 2016 | 141 |
| Adalimumab | RA | NS | Heart failure | 2 | 0,03 | 1722 medical centers | Prospective | 7740 | Japan | 2014 | 139 |
| Adalimumab | RA | NS | Herpes zoster | 1 | 0,93 | Rheumatoid Arthritis Reference Center | Retrospective | 108 | Colombia | 2019 | 118 |
| Adalimumab | RA | Humira | Herpes zoster | 2 | 1,85 | Rheumatoid Arthritis Reference Center | Retrospective | 108 | Colombia | 2016 | 141 |
| Adalimumab | RA | NS | Herpes zoster | 56 | 0,72 | 1722 medical centers | Prospective | 7740 | Japan | 2014 | 139 |
| Adalimumab | RA | NS | Hospitalization because of an infection | 77 | - | National Registry of Biological Treatment in Finland and hospital records of the Central Hospital of Central Finland | Prospective | NS | Finland | 2015 | 108 |
| Adalimumab | RA | NS | Hypersensitivity reactions | 1 | 0,62 | 76 centers | Prospective | 162 | 15 Australasia, Europe, North and South america countries | 2013 | 122 |
| Adalimumab | RA | NS | Infections | 106 | 65,43 | 76 centers | Prospective | 162 | 15 Australasia, Europe, North and South america countries | 2013 | 122 |
| Adalimumab | RA | NS | Infections and infestations | 538 | 6,95 | 1722 medical centers | Prospective | 7740 | Japan | 2014 | 139 |
| Adalimumab | RA | NS | Influenza | 14 | 0,18 | 1722 medical centers | Prospective | 7740 | Japan | 2014 | 139 |
| Adalimumab | RA | NS | Interstitial pneumonial | 52 | 0,67 | 1722 medical centers | Prospective | 7740 | Japan | 2014 | 139 |
| Adalimumab | RA | Humira | Leukopenia | 3 | 2,78 | Rheumatoid Arthritis Reference Center | Retrospective | 108 | Colombia | 2016 | 141 |
| Adalimumab | RA | Humira | Local reaction | 0 | 0 | Rheumatoid Arthritis Reference Center | Retrospective | 108 | Colombia | 2016 | 141 |
| Adalimumab | RA | NS | Lower respiratory tract infection | 12 | 1,5 | GISEA | Ambispective | 802 | Italia | 2012 | 111 |
| Adalimumab | RA | Humira | Lupus like | 2 | 1,85 | Rheumatoid Arthritis Reference Center | Retrospective | 108 | Colombia | 2016 | 141 |
| Adalimumab | RA | Humira | Lymphomas | 15 | 0,23 | ReAct/ReAlise | Prospective | 6610 | Multicentric | 2014 | 120 |
| Adalimumab | RA | NS | Malignant diseases | 8 | 0,51 | NHIRD | Retrospective | 1577 | Taiwan | 2014 | 150 |
| Adalimumab | RA | NS | Malignant neoplasm | 13 | 0,17 | 1722 medical centers | Prospective | 7740 | Japan | 2014 | 139 |
| Adalimumab | RA | NS | Miller – Fisher syndrome | 1 | NS | BIOGEAS registro 2009 | Prospective | NS | Spain | 2011 | 119 |
| Adalimumab | RA | NS | Multiple monoeuritis | 1 | NS | BIOGEAS registro 2009 | Prospective | NS | Spain | 2011 | 119 |
| Adalimumab | RA | Humira | Myalgia | 0 | 0 | Rheumatoid Arthritis Reference Center | Retrospective | 108 | Colombia | 2016 | 141 |
| Adalimumab | RA | NS | Myocardial infarction or acute coronary syndrome | 2 | 1,23 | 76 centers | Prospective | 162 | 15 Australasia, Europe, North and South america countries | 2013 | 122 |
| Adalimumab | RA | Humira | Neoplasms | 121 | 1,83 | ReAct/ReAlise | Prospective | 6610 | Multicentric | 2014 | 120 |
| Adalimumab | RA | NS | Optic neuritis | 1 | 1,3 | NS | Prospective | 77 | Greece | 2014 | 120 |
| Adalimumab | RA | NS | Osteoarticular | 1 | 0,12 | GISEA | Ambispective | 802 | Italia | 2012 | 111 |
| Adalimumab | RA | NS | Other | 14 | 12,96 | Rheumatoid Arthritis Reference Center | Retrospective | 108 | Colombia | 2019 | 118 |
| Adalimumab | RA | Humira | Other | 2 | 1,85 | Rheumatoid Arthritis Reference Center | Retrospective | 108 | Colombia | 2016 | 141 |
| Adalimumab | RA | NS | Pancytopenia | 4 | 0,05 | 1722 medical centers | Prospective | 7740 | Japan | 2014 | 139 |
| Adalimumab | RA | Humira | Paresthesia | 0 | 0 | Rheumatoid Arthritis Reference Center | Retrospective | 108 | Colombia | 2016 | 141 |
| Adalimumab | RA | NS | Parotiditis | 1 | 0,62 | 76 centers | Prospective | 162 | 15 Australasia, Europe, North and South america countries | 2013 | 122 |
| Adalimumab | RA | NS | Pneumocystis jiroveci (carinii) Pneumonia | 26 | 0,34 | 1722 medical centers | Prospective | 7740 | Japan | 2014 | 139 |
| Adalimumab | RA | NS | Pneumonia | 2 | 1,85 | Rheumatoid Arthritis Reference Center | Retrospective | 108 | Colombia | 2019 | 118 |
| Adalimumab | RA | Humira | Pneumonia | 2 | 1,85 | Rheumatoid Arthritis Reference Center | Retrospective | 108 | Colombia | 2016 | 141 |
| Adalimumab | RA | NS | Pneumonia | 103 | 1,33 | 1722 medical centers | Prospective | 7740 | Japan | 2014 | 139 |
| Adalimumab | RA | Humira | Polyarthralgias increase | 0 | 0 | Rheumatoid Arthritis Reference Center | Retrospective | 108 | Colombia | 2016 | 141 |
| Adalimumab | RA | NS | Psoriasis | 3 | 2,78 | Rheumatoid Arthritis Reference Center | Retrospective | 108 | Colombia | 2019 | 118 |
| Adalimumab | RA | Humira | Psoriasis | 2 | 1,85 | Rheumatoid Arthritis Reference Center | Retrospective | 108 | Colombia | 2016 | 141 |
| Adalimumab | RA | NS | Rash | 16 | 14,81 | Rheumatoid Arthritis Reference Center | Retrospective | 108 | Colombia | 2019 | 118 |
| Adalimumab | RA | NS | Reactions at the administration site | 317 | 4,1 | 1722 medical centers | Prospective | 7740 | Japan | 2014 | 139 |
| Adalimumab | RA | NS | Respiratory, thoracic and mediastinal disorders | 210 | 2,71 | 1722 medical centers | Prospective | 7740 | Japan | 2014 | 139 |
| Adalimumab | RA | NS | Sepsis | 0 | 0 | Rheumatoid Arthritis Reference Center | Retrospective | 108 | Colombia | 2019 | 118 |
| Adalimumab | RA | NS | Sepsis | 15 | 0,19 | 1722 medical centers | Prospective | 7740 | Japan | 2014 | 139 |
| Adalimumab | RA | Humira | Septic arthritis | 0 | 0 | Rheumatoid Arthritis Reference Center | Retrospective | 108 | Colombia | 2016 | 141 |
| Adalimumab | RA | Humira | Serious adverse events | 58 | 0,88 | ReAct/ReAlise | Prospective | 6610 | Multicentric | 2014 | 120 |
| Adalimumab | RA | Humira | Serius infections | 518 | 7,84 | ReAct/ReAlise | Prospective | 6610 | Multicentric | 2014 | 120 |
| Adalimumab | RA | NS | Serius infections | 7 | 4,32 | 76 centers | Prospective | 162 | 15 Australasia, Europe, North and South america countries | 2013 | 122 |
| Adalimumab | RA | NS | Sinusitis | 1 | 0,62 | 76 centers | Prospective | 162 | 15 Australasia, Europe, North and South america countries | 2013 | 122 |
| Adalimumab | RA | NS | Skin and soft tissue infections | 10 | 1,25 | GISEA | Ambispective | 802 | Italia | 2012 | 111 |
| Adalimumab | RA | NS | Skin and subcutaneous tissue disorders | 560 | 7,24 | 1722 medical centers | Prospective | 7740 | Japan | 2014 | 139 |
| Adalimumab | RA | Humira | Stroke | 56 | 0,85 | ReAct/ReAlise | Prospective | 6610 | Multicentric | 2014 | 120 |
| Adalimumab | RA | NS | Stroke | 1 | 0,62 | 76 centers | Prospective | 162 | 15 Australasia, Europe, North and South america countries | 2013 | 122 |
| Adalimumab | RA | NS | Stroke | 2 | 1,85 | Rheumatoid Arthritis Reference Center | Retrospective | 108 | Colombia | 2019 | 118 |
| Adalimumab | RA | Humira | Stroke | 2 | 1,85 | Rheumatoid Arthritis Reference Center | Retrospective | 108 | Colombia | 2016 | 141 |
| Adalimumab | RA | Humira | Tachycardia | 0 | 0 | Rheumatoid Arthritis Reference Center | Retrospective | 108 | Colombia | 2016 | 141 |
| Adalimumab | RA | Humira | TB | 35 | 0,53 | ReAct/ReAlise | Prospective | 6610 | Multicentric | 2014 | 120 |
| Adalimumab | RA | NS | TB | 3 | 2,78 | Rheumatoid Arthritis Reference Center | Retrospective | 108 | Colombia | 2019 | 118 |
| Adalimumab | RA | Humira | TB | 4 | 3,7 | Rheumatoid Arthritis Reference Center | Retrospective | 108 | Colombia | 2016 | 141 |
| Adalimumab | RA | NS | TB | 9 | 0,12 | 1722 medical centers | Prospective | 7740 | Japan | 2014 | 139 |
| Adalimumab | RA | NS | TB | 5 | 1,45 | NHIRDy certificados de defunción | Retrospective | 345 | Taiwan | 2013 | 128 |
| Adalimumab | RA | Humira | Therapeutic failure | 47 | 0,71 | ReAct/ReAlise | Prospective | 6610 | Multicentric | 2014 | 120 |
| Adalimumab | RA | NS | Therapeutic failure | 20 | 11,63 | Padre Billini Teaching Hospital | Ambispective | 172 | Dominican Republic | 2018 | 133 |
| Adalimumab | RA | NS | Upper respiratory tract infection | 11 | 1,37 | GISEA | Ambispective | 802 | Italia | 2012 | 111 |
| Adalimumab | RA | NS | Urosepsis | 1 | 0,62 | 76 centers | Prospective | 162 | 15 Australasia, Europe, North and South america countries | 2013 | 122 |
| Adalimumab | RA | NS | UTI | 1 | 0,12 | GISEA | Ambispective | 802 | Italia | 2012 | 111 |
| Adalimumab | RA | NS | UTI | 1 | 0,62 | 76 centers | Prospective | 162 | 15 Australasia, Europe, North and South america countries | 2013 | 122 |
| Adalimumab | RA | Humira | UTI | 2 | 1,85 | Rheumatoid Arthritis Reference Center | Retrospective | 108 | Colombia | 2016 | 141 |
| Adalimumab | RA | NS | Viral labyrinthitis | 1 | 0,62 | 76 centers | Prospective | 162 | 15 Australasia, Europe, North and South america countries | 2013 | 122 |
| All | RD | NS | Arthritis bacterial | 4 | 0,38 | BIOBADABRAIL | Prospective | 1039 | Brazil | 2011 | 21 |
| All | RD | NS | Bronchitis | 6 | 0,58 | BIOBADABRAIL | Prospective | 1039 | Brazil | 2011 | 21 |
| All | RD | NS | Conjunctivitis | 2 | 0,19 | BIOBADABRAIL | Prospective | 1039 | Brazil | 2011 | 21 |
| All | RD | NS | Gastrointestinal infection | 4 | 0,38 | BIOBADABRAIL | Prospective | 1039 | Brazil | 2011 | 21 |
| All | RD | NS | Genital candidiasis | 4 | 0,38 | BIOBADABRAIL | Prospective | 1039 | Brazil | 2011 | 21 |
| All | RD | NS | Herpes zoster | 10 | 0,96 | BIOBADABRAIL | Prospective | 1039 | Brazil | 2011 | 21 |
| All | RD | NS | Muscle abscess | 1 | 0,1 | BIOBADABRAIL | Prospective | 1039 | Brazil | 2011 | 21 |
| All | RD | NS | Otitis | 1 | 0,1 | BIOBADABRAIL | Prospective | 1039 | Brazil | 2011 | 21 |
| All | RD | NS | Pneumonia | 12 | 1,15 | BIOBADABRAIL | Prospective | 1039 | Brazil | 2011 | 21 |
| All | RD | NS | Prosthetic joint infection | 1 | 0,1 | BIOBADABRAIL | Prospective | 1039 | Brazil | 2011 | 21 |
| All | RD | NS | Pyelonephritis | 4 | 0,38 | BIOBADABRAIL | Prospective | 1039 | Brazil | 2011 | 21 |
| All | RD | NS | Sepsis | 1 | 0,1 | BIOBADABRAIL | Prospective | 1039 | Brazil | 2011 | 21 |
| All | RD | NS | Skin infection | 39 | 3,75 | BIOBADABRAIL | Prospective | 1039 | Brazil | 2011 | 21 |
| All | RD | NS | Stomatitis | 2 | 0,19 | BIOBADABRAIL | Prospective | 1039 | Brazil | 2011 | 21 |
| All | RD | NS | TB | 3 | 0,29 | BIOBADABRAIL | Prospective | 1039 | Brazil | 2011 | 21 |
| All | RD | NS | Tuberculoid leprosy | 1 | 0,1 | BIOBADABRAIL | Prospective | 1039 | Brazil | 2011 | 21 |
| All | RD | NS | Upper respiratory tract infection | 59 | 5,68 | BIOBADABRAIL | Prospective | 1039 | Brazil | 2011 | 21 |
| All | RD | NS | UTI | 57 | 5,49 | BIOBADABRAIL | Prospective | 1039 | Brazil | 2011 | 21 |
| Etanercept | JIA | NS | Abdominal pain | 1 | 0,68 | Dutch national register | Prospective | 146 | Holland | 2009 | 137 |
| Etanercept | JIA | NS | Abscess | 2 | 1,9 | Pediatric Rheumatology Collaborative Study Group | Prospective | 103 | United States and Canada | 2009 | 123 |
| Etanercept | JIA | NS | Abscess | 0 | 0 | Pediatric Rheumatology Collaborative Study Group | Prospective | 103 | United States and Canada | 2009 | 123 |
| Etanercept | JIA | NS | Acne | 1 | 0,98 | Medical centers | Prospective | 102 | Japan | 2018 | 136 |
| Etanercept | RA | Etanar | Acne | 2 | 2,17 | Rheumatoid Arthritis Reference Center | Retrospective | 92 | Colombia | 2016 | 140 |
| Etanercept | RA | NS | Allergy treatment suspension | 12 | 2,71 | REAL | Prospective | 442 | Japan | 2012 | 140 |
| Etanercept | JIA | NS | Allergic reaction | 1 | 1,72 | NS | Prospective | 58 | United States | 2008 | 132 |
| Etanercept | RA | Etanar | Angioedema | 0 | 0 | Rheumatoid Arthritis Reference Center | Retrospective | 92 | Colombia | 2016 | 141 |
| Etanercept | RA | Etanar | Breast mass, under study | 0 | 0 | Rheumatoid Arthritis Reference Center | Retrospective | 92 | Colombia | 2016 | 141 |
| Etanercept | RA | NS | Candidiasis | 0 | 0 | Rheumatoid Arthritis Reference Center | Retrospective | 81 | Colombia | 2019 | 118 |
| Etanercept | RA | Enbrel | Candidiasis | 0 | 0 | Rheumatoid Arthritis Reference Center | Retrospective | 92 | Colombia | 2019 | 118 |
| Etanercept | RA | Etanar | Candidiasis | 0 | 0 | Rheumatoid Arthritis Reference Center | Retrospective | 92 | Colombia | 2016 | 141 |
| Etanercept | RA | Enbrel | Cellulitis | 0 | 0 | Rheumatoid Arthritis Reference Center | Retrospective | 92 | Colombia | 2019 | 118 |
| Etanercept | RA | NS | Cellulitis | 1 | 1,23 | Rheumatoid Arthritis Reference Center | Retrospective | 81 | Colombia | 2019 | 118 |
| Etanercept | RA | Etanar | Cellulitis | 0 | 0 | Rheumatoid Arthritis Reference Center | Retrospective | 92 | Colombia | 2016 | 141 |
| Etanercept | RA | Etanar | Cervical cancer | 0 | 0 | Rheumatoid Arthritis Reference Center | Retrospective | 92 | Colombia | 2016 | 141 |
| Etanercept | RA | Etanar | Chilblains | 0 | 0 | Rheumatoid Arthritis Reference Center | Retrospective | 92 | Colombia | 2016 | 141 |
| Etanercept | PsA | NS | TB | 1 | NS | AERS of the FDA | Retrospective | NS | United States. Europe e India | 2004 | 134 |
| Etanercept | JIA | NS | Alopecia | 1 | 0,98 | Medical centers | Prospective | 102 | Japan | 2018 | 136 |
| Etanercept | JIA | NS | Arthralgia | 1 | 1,72 | NS | Prospective | 58 | United States | 2008 | 132 |
| Etanercept | JIA | NS | Arthritis disease flare | 6 | 10,34 | NS | Prospective | 58 | United States | 2008 | 132 |
| Etanercept | JIA | NS | Bacterial infection | 0 | 0 | Pediatric Rheumatology Collaborative Study Group | Prospective | 103 | United States and Canada | 2009 | 123 |
| Etanercept | JIA | NS | Bacterial infection | 0 | 0 | Pediatric Rheumatology Collaborative Study Group | Prospective | 103 | United States and Canada | 2009 | 123 |
| Etanercept | JIA | NS | Blood culture positive | 1 | 1 | Pediatric Rheumatology Collaborative Study Group | Prospective | 103 | United States and Canada | 2009 | 123 |
| Etanercept | JIA | NS | Blood culture positive | 0 | 0 | Pediatric Rheumatology Collaborative Study Group | Prospective | 103 | United States and Canada | 2009 | 123 |
| Etanercept | JIA | NS | Bronchitis | 1 | 1 | Pediatric Rheumatology Collaborative Study Group | Prospective | 103 | United States and Canada | 2009 | 123 |
| Etanercept | JIA | NS | Bronchitis | 0 | 0 | Pediatric Rheumatology Collaborative Study Group | Prospective | 103 | United States and Canada | 2009 | 123 |
| Etanercept | JIA | Intacept | Cellulitis | 1 | 3,33 | Institute of Child Health | Prospective | 30 | India | 2016 | 144 |
| Etanercept | JIA | NS | Chickenpox | 1 | 2,56 | Institute of Child Health | Prospective | 39 | India | 2016 | 144 |
| Etanercept | JIA | NS | Chronic cough | 2 | 1,37 | Dutch national register | Prospective | 146 | Holland | 2009 | 137 |
| Etanercept | JIA | NS | Colitis | 1 | 1 | Pediatric Rheumatology Collaborative Study Group | Prospective | 103 | United States and Canada | 2009 | 123 |
| Etanercept | JIA | NS | Colitis | 0 | 0 | Pediatric Rheumatology Collaborative Study Group | Prospective | 103 | United States and Canada | 2009 | 123 |
| Etanercept | JIA | NS | Concentration disorder | 3 | 2,05 | Dutch national register | Prospective | 146 | Holland | 2009 | 137 |
| Etanercept | JIA | NS | Crohn´s disease | 1 | 0,68 | Dutch national register | Prospective | 146 | Holland | 2009 | 137 |
| Etanercept | JIA | NS | Death | 0 | 0 | NS | Prospective | 58 | United States | 2008 | 132 |
| Etanercept | JIA | NS | Dermatitis herpetiformis | 2 | 1,96 | Medical centers | Prospective | 102 | Japan | 2018 | 136 |
| Etanercept | JIA | NS | Diarrhea | 1 | 0,98 | Medical centers | Prospective | 102 | Japan | 2018 | 136 |
| Etanercept | JIA | NS | Discomfort | 1 | 0,98 | Medical centers | Prospective | 102 | Japan | 2018 | 136 |
| Etanercept | JIA | NS | Epistaxis | 1 | 0,98 | Medical centers | Prospective | 102 | Japan | 2018 | 136 |
| Etanercept | JIA | NS | Epstein–Barr virus infection | 1 | 0,68 | Dutch national register | Prospective | 146 | Holland | 2009 | 137 |
| Etanercept | JIA | NS | Extrapulmonary TB | 2 | NS | AERS of the FDA | Retrospective | NS | United States. Europe e India | 2004 | 134 |
| Etanercept | JIA | NS | Eye disorders | 1 | 0,98 | Medical centers | Prospective | 102 | Japan | 2018 | 136 |
| Etanercept | JIA | NS | Fatigue | 5 | 3,42 | Dutch national register | Prospective | 146 | Holland | 2009 | 137 |
| Etanercept | JIA | NS | Fatigue | 1 | 0,98 | Medical centers | Prospective | 102 | Japan | 2018 | 136 |
| Etanercept | JIA | NS | Fever of unknown origin | 7 | 4,79 | Dutch national register | Prospective | 146 | Holland | 2009 | 137 |
| Etanercept | JIA | NS | Gastritis | 1 | 0,98 | Medical centers | Prospective | 102 | Japan | 2018 | 136 |
| Etanercept | JIA | NS | Gastroenteritis | 1 | 1 | Pediatric Rheumatology Collaborative Study Group | Prospective | 103 | United States and Canada | 2009 | 123 |
| Etanercept | JIA | NS | Gastroenteritis | 0 | 0 | Pediatric Rheumatology Collaborative Study Group | Prospective | 103 | United States and Canada | 2009 | 123 |
| Etanercept | JIA | NS | Gastroenteritis | 1 | 0,98 | Medical centers | Prospective | 102 | Japan | 2018 | 136 |
| Etanercept | JIA | NS | Gastroenteritis viral | 1 | 0,98 | Medical centers | Prospective | 102 | Japan | 2018 | 136 |
| Etanercept | JIA | NS | Gastrointestinal disorders | 2 | 1,96 | Medical centers | Prospective | 102 | Japan | 2018 | 136 |
| Etanercept | JIA | NS | Gastrointestinal infection | 2 | 1,37 | Dutch national register | Prospective | 146 | Holland | 2009 | 137 |
| Etanercept | JIA | NS | General disorders and administration site conditions | 7 | 6,86 | Medical centers | Prospective | 102 | Japan | 2018 | 136 |
| Etanercept | JIA | NS | Hair loss | 2 | 1,37 | Dutch national register | Prospective | 146 | Holland | 2009 | 137 |
| Etanercept | JIA | NS | Headache | 6 | 4,11 | Dutch national register | Prospective | 146 | Holland | 2009 | 137 |
| Etanercept | JIA | NS | Headache | 1 | 0,98 | Medical centers | Prospective | 102 | Japan | 2018 | 136 |
| Etanercept | JIA | NS | Hemolytic anemia | 1 | 2,56 | Institute of Child Health | Prospective | 39 | India | 2016 | 144 |
| Etanercept | JIA | NS | Herpes | 1 | 0,68 | Dutch national register | Prospective | 146 | Holland | 2009 | 137 |
| Etanercept | JIA | NS | Herpes Zoster | 2 | 1,9 | Pediatric Rheumatology Collaborative Study Group | Prospective | 103 | United States and Canada | 2009 | 123 |
| Etanercept | JIA | NS | Herpes Zoster | 0 | 0 | Pediatric Rheumatology Collaborative Study Group | Prospective | 103 | United States and Canada | 2009 | 123 |
| Etanercept | JIA | NS | Infection ear lobe | 1 | 0,68 | Dutch national register | Prospective | 146 | Holland | 2009 | 137 |
| Etanercept | JIA | NS | Infections | 2 | 1,9 | Pediatric Rheumatology Collaborative Study Group | Prospective | 103 | United States and Canada | 2009 | 123 |
| Etanercept | JIA | NS | Infections | 1 | 1 | Pediatric Rheumatology Collaborative Study Group | Prospective | 103 | United States and Canada | 2009 | 123 |
| Etanercept | JIA | NS | Infections | 17 | 11,64 | Dutch national register | Prospective | 146 | Holland | 2009 | 137 |
| Etanercept | JIA | Enbrel | Infections | 3 | 5,56 | Switzerland database | Prospective | 54 | Suiza | 2010 | 143 |
| Etanercept | JIA | NS | Infections and infestations | 7 | 6,86 | Medical centers | Prospective | 102 | Japan | 2018 | 136 |
| Etanercept | JIA | NS | Influenza | 3 | 2,94 | Medical centers | Prospective | 102 | Japan | 2018 | 136 |
| Etanercept | JIA | NS | Injection site erythema | 1 | 0,98 | Medical centers | Prospective | 102 | Japan | 2018 | 136 |
| Etanercept | JIA | NS | Injection site reaction | 7 | 4,79 | Dutch national register | Prospective | 146 | Holland | 2009 | 137 |
| Etanercept | JIA | NS | Injection site reactions | 5 | 4,9 | Medical centers | Prospective | 102 | Japan | 2018 | 136 |
| Etanercept | JIA | NS | Iritis | 1 | 0,98 | Medical centers | Prospective | 102 | Japan | 2018 | 136 |
| Etanercept | JIA | NS | Liver function test abnormal | 2 | 1,96 | Medical centers | Prospective | 102 | Japan | 2018 | 136 |
| Etanercept | JIA | NS | Liver function test abnormal | 1 | 0,98 | Medical centers | Prospective | 102 | Japan | 2018 | 136 |
| Etanercept | JIA | Enbrel | Liver function test abnormal | 1 | 1,85 | Switzerland database | Prospective | 54 | Suiza | 2010 | 143 |
| Etanercept | JIA | NS | Loss of efficiency | 7 | 12,1 | NS | Prospective | 58 | United States | 2008 | 132 |
| Etanercept | JIA | NS | Lymphadenopathy | 1 | 0,68 | Dutch national register | Prospective | 146 | Holland | 2009 | 137 |
| Etanercept | JIA | NS | Nervous system disorders | 1 | 0,98 | Medical centers | Prospective | 102 | Japan | 2018 | 136 |
| Etanercept | JIA | NS | Osteoporosis | 1 | 0,68 | Dutch national register | Prospective | 146 | Holland | 2009 | 137 |
| Etanercept | JIA | NS | Otitis | 2 | 1,37 | Dutch national register | Prospective | 146 | Holland | 2009 | 137 |
| Etanercept | JIA | NS | Pharyngitis | 1 | 1 | Pediatric Rheumatology Collaborative Study Group | Prospective | 103 | United States and Canada | 2009 | 121 |
| Etanercept | JIA | NS | Pharyngitis | 0 | 0 | Pediatric Rheumatology Collaborative Study Group | Prospective | 103 | United States and Canada | 2009 | 123 |
| Etanercept | RA, AS and PsA | NS | Neutropenia | 49 | 18,4 | Rheumatology Department's database at the Derbyshire Royal Infirmary | Retrospective | 267 | United Kingdom | 2010 | 124 |
| Etanercept | RA, AS and PsA | NS | Severe infections associated with neutropenia | 1 | 0,4 | Rheumatology Department's database at the Derbyshire Royal Infirmary | Retrospective | 267 | United Kingdom | 2010 | 124 |
| Etanercept | RA | NS | Dermatitis | 2 | 2,47 | Rheumatoid Arthritis Reference Center | Retrospective | 81 | Colombia | 2019 | 118 |
| Etanercept | JIA | NS | Pharyngitis | 2 | 1,96 | Medical centers | Prospective | 102 | Japan | 2018 | 136 |
| Etanercept | JIA | NS | Pruritus | 1 | 0,98 | Medical centers | Prospective | 102 | Japan | 2018 | 136 |
| Etanercept | JIA | NS | Pyelonephritis | 1 | 1,72 | NS | Prospective | 58 | United States | 2008 | 132 |
| Etanercept | JIA | NS | Pyelonephritis | 2 | 1,9 | Pediatric Rheumatology Collaborative Study Group | Prospective | 103 | United States and Canada | 2009 | 123 |
| Etanercept | JIA | NS | Pyelonephritis | 0 | 0 | Pediatric Rheumatology Collaborative Study Group | Prospective | 103 | United States and Canada | 2009 | 123 |
| Etanercept | JIA | Enbrel | Reactions associated infusion | 1 | 1,85 | Switzerland database | Prospective | 54 | Suiza | 2010 | 143 |
| Etanercept | JIA | NS | Sarcoidosis | 2 | 1,37 | Dutch national register | Prospective | 146 | Holland | 2009 | 137 |
| Etanercept | JIA | NS | Seizures | 1 | 0,68 | Dutch national register | Prospective | 146 | Holland | 2009 | 137 |
| Etanercept | JIA | NS | Sepsis | 1 | 1 | Pediatric Rheumatology Collaborative Study Group | Prospective | 103 | United States and Canada | 2009 | 123 |
| Etanercept | JIA | NS | Sepsis | 0 | 0 | Pediatric Rheumatology Collaborative Study Group | Prospective | 103 | United States and Canada | 2009 | 123 |
| Etanercept | JIA | NS | Serious adverse events | 9 | 6,16 | Dutch national register | Prospective | 146 | Holland | 2009 | 137 |
| Etanercept | JIA | NS | Serius gastrointestinal infection | 3 | 2,05 | Dutch national register | Prospective | 146 | Holland | 2009 | 137 |
| Etanercept | JIA | NS | Sickness | 8 | 5,48 | Dutch national register | Prospective | 146 | Holland | 2009 | 137 |
| Etanercept | JIA | NS | Sinusitis | 0 | 0 | Pediatric Rheumatology Collaborative Study Group | Prospective | 103 | United States and Canada | 2009 | 123 |
| Etanercept | JIA | NS | Sinusitis | 0 | 0 | Pediatric Rheumatology Collaborative Study Group | Prospective | 103 | United States and Canada | 2009 | 123 |
| Etanercept | JIA | NS | Skin and subcutaneous tissue disorders | 5 | 4,9 | Medical centers | Prospective | 102 | Japan | 2018 | 136 |
| Etanercept | JIA | NS | TB | 0 | 0 | NS | Prospective | 58 | United States | 2008 | 132 |
| Etanercept | JIA | NS | Ulcerative colitis | 1 | 0,68 | Dutch national register | Prospective | 146 | Holland | 2009 | 137 |
| Etanercept | JIA | NS | Upper respiratory tract infection | 2 | 1,37 | Dutch national register | Prospective | 146 | Holland | 2009 | 137 |
| Etanercept | JIA | NS | Upper respiratory tract inflammation | 6 | 5,88 | Medical centers | Prospective | 102 | Japan | 2018 | 136 |
| Etanercept | JIA | NS | Urosepsis (Escherichia coli and Enterococcus) | 1 | 0,68 | Dutch national register | Prospective | 146 | Holland | 2009 | 137 |
| Etanercept | JIA | NS | Urticaria | 1 | 0,98 | Medical centers | Prospective | 102 | Japan | 2018 | 136 |
| Etanercept | JIA | NS | Urticariform Rash, Late Maculopapular | 2 | 1,37 | Dutch national register | Prospective | 146 | Holland | 2009 | 137 |
| Etanercept | JIA | NS | UTI | 1 | 1 | Pediatric Rheumatology Collaborative Study Group | Prospective | 103 | United States and Canada | 2009 | 123 |
| Etanercept | JIA | NS | UTI | 0 | 0 | Pediatric Rheumatology Collaborative Study Group | Prospective | 103 | United States and Canada | 2009 | 123 |
| Etanercept | JIA | NS | Uveitis | 1 | 2,56 | Institute of Child Health | Prospective | 39 | India | 2016 | 144 |
| Etanercept | JIA | NS | Viral infections | 1 | 1 | Pediatric Rheumatology Collaborative Study Group | Prospective | 103 | United States and Canada | 2009 | 123 |
| Etanercept | RA | Enbrel | Dermatitis | 5 | 5,43 | Rheumatoid Arthritis Reference Center | Retrospective | 92 | Colombia | 2019 | 118 |
| Etanercept | RA | Etanar | Dermatitis | 8 | 8,7 | Rheumatoid Arthritis Reference Center | Retrospective | 92 | Colombia | 2016 | 141 |
| Etanercept | RA | NS | Dermatomycosis | 0 | 0 | Rheumatoid Arthritis Reference Center | Retrospective | 81 | Colombia | 2019 | 118 |
| Etanercept | RA | Enbrel | Dermatomycosis | 0 | 0 | Rheumatoid Arthritis Reference Center | Retrospective | 92 | Colombia | 2019 | 118 |
| Etanercept | RA | NS | Diarrhea | 0 | 0 | Rheumatoid Arthritis Reference Center | Retrospective | 81 | Colombia | 2019 | 118 |
| Etanercept | RA | Enbrel | Diarrhea | 4 | 4,35 | Rheumatoid Arthritis Reference Center | Retrospective | 92 | Colombia | 2019 | 118 |
| Etanercept | RA | Etanar | Diarrhea | 4 | 4,35 | Rheumatoid Arthritis Reference Center | Retrospective | 92 | Colombia | 2016 | 141 |
| Etanercept | RA | Etanar | Diarrhea | 1 | 0,95 | 13 medical centers | NS | 105 | Colombia | 2015 | 142 |
| Etanercept | RA | NS | Elevated blood pressure | 0 | 0 | Rheumatoid Arthritis Reference Center | Retrospective | 81 | Colombia | 2019 | 118 |
| Etanercept | RA | Enbrel | Elevated blood pressure | 0 | 0 | Rheumatoid Arthritis Reference Center | Retrospective | 92 | Colombia | 2019 | 118 |
| Etanercept | RA | Etanar | Elevated blood pressure | 0 | 0 | Rheumatoid Arthritis Reference Center | Retrospective | 92 | Colombia | 2016 | 141 |
| Etanercept | RA | NS | Extrapulmonary TB | 9 | NS | AERS of the FDA | Retrospective | NS | United States. Europe e India | 2004 | 134 |
| Etanercept | RA | NS | Headache | 1 | 1,23 | Rheumatoid Arthritis Reference Center | Retrospective | 81 | Colombia | 2019 | 118 |
| Etanercept | RA | Enbrel | Headache | 3 | 3,26 | Rheumatoid Arthritis Reference Center | Retrospective | 92 | Colombia | 2019 | 118 |
| Etanercept | RA | Etanar | Headache | 0 | 0 | Rheumatoid Arthritis Reference Center | Retrospective | 92 | Colombia | 2016 | 141 |
| Etanercept | RA | Etanar | Headache | 1 | 0,95 | 13 medical centers | NS | 105 | Colombia | 2015 | 142 |
| Etanercept | RA | Etanar | Herpes labialis | 1 | 0,95 | 13 medical centers | NS | 105 | Colombia | 2015 | 142 |
| Etanercept | RA | NS | Herpes zoster | 2 | 2,47 | Rheumatoid Arthritis Reference Center | Retrospective | 81 | Colombia | 2019 | 118 |
| Etanercept | RA | Enbrel | Herpes zoster | 1 | 1,09 | Rheumatoid Arthritis Reference Center | Retrospective | 92 | Colombia | 2019 | 118 |
| Etanercept | RA | Etanar | Herpes zoster | 1 | 1,09 | Rheumatoid Arthritis Reference Center | Retrospective | 92 | Colombia | 2016 | 141 |
| Etanercept | RA | NS | Hospitalization because of an infection | 68 | - | National Registry of Biological Treatment in Finland and hospital records of the Central Hospital of Central Finland | Prospective | NS | Finland | 2015 | 108 |
| Etanercept | RA | NS | Infection treatment suspension | 22 | 4,98 | REAL | Prospective | 442 | Japan | 2012 | 140 |
| Etanercept | RA | Etanar | Injection site reaction | 1 | 0,95 | 13 medical centers | NS | 105 | Colombia | 2015 | 142 |
| Etanercept | RA | NS | Intra-abdominal infections | 4 | 0,35 | GISEA | Ambispective | 1130 | Italia | 2012 | 111 |
| Etanercept | RA | Etanar | Leukopenia | 0 | 0 | Rheumatoid Arthritis Reference Center | Retrospective | 92 | Colombia | 2016 | 141 |
| Etanercept | RA | Etanar | Leukopenia | 1 | 0,95 | 13 medical centers | NS | 105 | Colombia | 2015 | 142 |
| Etanercept | RA | Etanar | Local reaction | 3 | 3,26 | Rheumatoid Arthritis Reference Center | Retrospective | 92 | Colombia | 2016 | 141 |
| Etanercept | RA | NS | Lower respiratory tract infection | 6 | 0,53 | GISEA | Ambispective | 1130 | Italia | 2012 | 111 |
| Etanercept | RA | Etanar | Lower member edema | 1 | 0,95 | 13 medical centers | NS | 105 | Colombia | 2015 | 142 |
| Etanercept | RA | Etanar | Lupus like | 0 | 0 | Rheumatoid Arthritis Reference Center | Retrospective | 92 | Colombia | 2016 | 141 |
| Etanercept | RA | NS | Malignant diseases | 71 | 2,17 | NHIRD | Retrospective | 3270 | Taiwan | 2014 | 150 |
| Etanercept | RA | Etanar | Myalgia | 0 | 0 | Rheumatoid Arthritis Reference Center | Retrospective | 92 | Colombia | 2016 | 141 |
| Etanercept | RA | NS | Myelitis | 2 | 0,02 | BIOGEAS registro 2009 | Prospective | 10930 | Spain | 2011 | 119 |
| Etanercept | RA | NS | Osteoarticular infection | 3 | 0,27 | GISEA | Ambispective | 1130 | Italia | 2012 | 111 |
| Etanercept | RA | NS | Other | 1 | 1,23 | Rheumatoid Arthritis Reference Center | Retrospective | 81 | Colombia | 2019 | 118 |
| Etanercept | RA | Enbrel | Other | 9 | 9,78 | Rheumatoid Arthritis Reference Center | Retrospective | 92 | Colombia | 2019 | 118 |
| Etanercept | RA | Etanar | Other | 5 | 5,43 | Rheumatoid Arthritis Reference Center | Retrospective | 92 | Colombia | 2016 | 141 |
| Etanercept | RA | Etanar | Paresthesia | 2 | 2,17 | Rheumatoid Arthritis Reference Center | Retrospective | 92 | Colombia | 2016 | 141 |
| Etanercept | RA | Etanar | Peripheral neuropathy | 1 | 0,95 | 13 medical centers | NS | 105 | Colombia | 2015 | 142 |
| Etanercept | RA | Enbrel | Pneumonia | 0 | 0 | Rheumatoid Arthritis Reference Center | Retrospective | 92 | Colombia | 2019 | 118 |
| Etanercept | RA | NS | Pneumonia | 2 | 2,47 | Rheumatoid Arthritis Reference Center | Retrospective | 81 | Colombia | 2019 | 118 |
| Etanercept | RA | Etanar | Pneumonia | 0 | 0 | Rheumatoid Arthritis Reference Center | Retrospective | 92 | Colombia | 2016 | 141 |
| Etanercept | RA | Etanar | Polyarthralgias increase | 1 | 1,09 | Rheumatoid Arthritis Reference Center | Retrospective | 92 | Colombia | 2016 | 141 |
| Etanercept | RA | NS | Psoriasis | 0 | 0 | Rheumatoid Arthritis Reference Center | Retrospective | 81 | Colombia | 2019 | 118 |
| Etanercept | RA | Enbrel | Psoriasis | 0 | 0 | Rheumatoid Arthritis Reference Center | Retrospective | 92 | Colombia | 2019 | 118 |
| Etanercept | RA | Etanar | Psoriasis | 1 | 1,09 | Rheumatoid Arthritis Reference Center | Retrospective | 92 | Colombia | 2016 | 141 |
| Etanercept | RA | Etanar | Purito | 5 | 4,76 | 13 medical centers | NS | 105 | Colombia | 2015 | 142 |
| Etanercept | RA | NS | Rash | 4 | 4,94 | Rheumatoid Arthritis Reference Center | Retrospective | 81 | Colombia | 2019 | 118 |
| Etanercept | RA | Enbrel | Rash | 6 | 6,52 | Rheumatoid Arthritis Reference Center | Retrospective | 92 | Colombia | 2019 | 118 |
| Etanercept | RA | Etanar | Rhinitis | 3 | 2,86 | 13 medical centers | NS | 105 | Colombia | 2015 | 142 |
| Etanercept | RA | NS | Sepsis | 1 | 0,09 | GISEA | Ambispective | 1130 | Italia | 2012 | 111 |
| Etanercept | RA | Enbrel | Sepsis | 0 | 0 | Rheumatoid Arthritis Reference Center | Retrospective | 92 | Colombia | 2019 | 118 |
| Etanercept | RA | NS | Sepsis | 1 | 1,23 | Rheumatoid Arthritis Reference Center | Retrospective | 81 | Colombia | 2019 | 118 |
| Etanercept | RA | NS | Sepsis due to Listeria monocytogenesis | 1 | NS | FDA Adverse Event Reporting System | Retrospective | NS | Norway | 2003 | 146 |
| Etanercept | RA | Etanar | Septic arthritis | 0 | 0 | Rheumatoid Arthritis Reference Center | Retrospective | 92 | Colombia | 2016 | 141 |
| Etanercept | RA | NS | Serius infections | 33 | 2,92 | GISEA | Ambispective | 1130 | Italia | 2012 | 111 |
| Etanercept | RA | NS | Skin and soft tissue infections | 7 | 0,62 | GISEA | Ambispective | 1130 | Italia | 2012 | 111 |
| Etanercept | RA | NS | Stroke | 0 | 0 | Rheumatoid Arthritis Reference Center | Retrospective | 81 | Colombia | 2019 | 118 |
| Etanercept | RA | Enbrel | Stroke | 0 | 0 | Rheumatoid Arthritis Reference Center | Retrospective | 92 | Colombia | 2019 | 118 |
| Etanercept | RA | Etanar | Stroke | 0 | 0 | Rheumatoid Arthritis Reference Center | Retrospective | 92 | Colombia | 2016 | 141 |
| Etanercept | RA | NS | Suspension of treatment due to loss of efficacy | 47 | 10,63 | REAL | Prospective | 442 | Japan | 2012 | 140 |
| Etanercept | RA | NS | Suspension of treatment for adverse events | 57 | 12,9 | REAL | Prospective | 442 | Japan | 2012 | 140 |
| Etanercept | RA | NS | Suspension of treatment for cardiovascular disease | 2 | 0,45 | REAL | Prospective | 442 | Japan | 2012 | 140 |
| Etanercept | RA | NS | Suspension of treatment for malignancy | 3 | 0,68 | REAL | Prospective | 442 | Japan | 2012 | 140 |
| Etanercept | RA | NS | Suspension of treatment for non-infectious lung disease | 7 | 1,58 | REAL | Prospective | 442 | Japan | 2012 | 140 |
| Etanercept | RA | Etanar | Tachycardia | 0 | 0 | Rheumatoid Arthritis Reference Center | Retrospective | 92 | Colombia | 2016 | 141 |
| Etanercept | RA | NS | TB | 10 | NS | AERS of the FDA | Retrospective | NS | United States. Europe e India | 2004 | 134 |
| Etanercept | RA | NS | TB | 1 | 0,09 | GISEA | Ambispective | 1130 | Italia | 2012 | 111 |
| Etanercept | RA | NS | TB | 0 | 0 | Rheumatoid Arthritis Reference Center | Retrospective | 81 | Colombia | 2019 | 118 |
| Etanercept | RA | Enbrel | TB | 2 | 2,17 | Rheumatoid Arthritis Reference Center | Retrospective | 92 | Colombia | 2019 | 118 |
| Etanercept | RA | Etanar | TB | 1 | 1,09 | Rheumatoid Arthritis Reference Center | Retrospective | 92 | Colombia | 2016 | 141 |
| Etanercept | RA | NS | TB | 4 | 0,83 | NHIRDy certificados de defunción | Retrospective | 484 | Taiwan | 2013 | 128 |
| Etanercept | RA | NS | Therapeutic failure | 8 | 7,55 | Padre Billini Teaching Hospital | Ambispective | 106 | Dominican Republic | 2018 | 133 |
| Etanercept | RA | NS | Upper respiratory tract infection | 5 | 0,44 | GISEA | Ambispective | 1130 | Italia | 2012 | 111 |
| Etanercept | RA | NS | UTI | 1 | 0,09 | GISEA | Ambispective | 1130 | Italia | 2012 | 111 |
| Etanercept | JIA | NS | Viral infections | 0 | 0 | Pediatric Rheumatology Collaborative Study Group | Prospective | 103 | United States and Canada | 2009 | 123 |
| Etanercept | JIA | NS | Vomiting | 1 | 0,68 | Dutch national register | Prospective | 146 | Holland | 2009 | 137 |
| Etanercept | JIA | NS | Weightloss | 1 | 0,68 | Dutch national register | Prospective | 146 | Holland | 2009 | 137 |
| Etanercept | RA | Etanar | UTI | 0 | 0 | Rheumatoid Arthritis Reference Center | Retrospective | 92 | Colombia | 2016 | 141 |
| Etanercept | RA | Etanar | Vascular ulcer | 1 | 0,95 | 13 medical centers | NS | 105 | Colombia | 2015 | 142 |
| Etanercept | SP | NS | Myelitis | 5 | NS | BIOGEAS registro 2006 | Prospective | NS | Spain | 2011 | 119 |
| Golimumab | RA | NS | Blood pressure decreased | 1 | 0,33 | 119 centers | Prospective | 301 | Japan | 2019 | 148 |
| Golimumab | RA | NS | Alkaline phosphatase increased | 1 | 0,33 | 119 centers | Prospective | 301 | Japan | 2019 | 148 |
| Golimumab | RA | NS | Bacterial pneumonia | 6 | 1,99 | 119 centers | Prospective | 301 | Japan | 2019 | 148 |
| Golimumab | RA | Subcutaneous Actemra | Benign, malignant and unspecified neoplasm | 25 | 0,49 | Janssen Pharmaceutical |  | 5138 | Japan | 2018 | 127 |
| Golimumab | RA | Subcutaneous Actemra | Blood and lymphatic system disorders | 36 | 0,7 | Janssen Pharmaceutical |  | 5139 | Japan | 2018 | 127 |
| Golimumab | RA | NS | Blood creatinine increased | 1 | 0,33 | 119 centers | Prospective | 301 | Japan | 2019 | 148 |
| Golimumab | RA | NS | Candidiasis | 0 | 0 | Rheumatoid Arthritis Reference Center | Retrospective | 47 | Colombia | 2019 | 118 |
| Golimumab | RA | Subcutaneous Actemra | Cardiac disorders | 22 | 0,43 | Janssen Pharmaceutical |  | 5147 | Japan | 2018 | 127 |
| Golimumab | RA | NS | Cellulitis | 0 | 0 | Rheumatoid Arthritis Reference Center | Retrospective | 47 | Colombia | 2019 | 118 |
| Golimumab | RA | NS | Cholesterol increased | 1 | 0,33 | 119 centers | Prospective | 301 | Japan | 2019 | 148 |
| Golimumab | RA | NS | Dermatitis | 0 | 0 | Rheumatoid Arthritis Reference Center | Retrospective | 47 | Colombia | 2019 | 118 |
| Golimumab | RA | NS | Dermatomycosis | 2 | 4,26 | Rheumatoid Arthritis Reference Center | Retrospective | 47 | Colombia | 2019 | 118 |
| Golimumab | RA | NS | Diarrhea | 0 | 0 | Rheumatoid Arthritis Reference Center | Retrospective | 47 | Colombia | 2019 | 118 |
| Golimumab | RA | Subcutaneous Actemra | Ear and labyrinth disorders | 4 | 0,08 | Janssen Pharmaceutical |  | 5146 | Japan | 2018 | 127 |
| Golimumab | RA | NS | Elevated blood pressure | 1 | 2,13 | Rheumatoid Arthritis Reference Center | Retrospective | 47 | Colombia | 2019 | 118 |
| Golimumab | RA | Subcutaneous Actemra | Endocrine disorders | 3 | 0,06 | Janssen Pharmaceutical |  | 5141 | Japan | 2018 | 127 |
| Golimumab | RA | Subcutaneous Actemra | Eye disorders | 8 | 0,16 | Janssen Pharmaceutical |  | 5145 | Japan | 2018 | 127 |
| Golimumab | RA | Subcutaneous Actemra | Gastrointestinal disorders | 104 | 2,02 | Janssen Pharmaceutical |  | 5150 | Japan | 2018 | 127 |
| Golimumab | RA | Subcutaneous Actemra | General disorders and administration site conditions | 73 | 1,42 | Janssen Pharmaceutical |  | 5156 | Japan | 2018 | 127 |
| Golimumab | RA | NS | Headache | 1 | 2,13 | Rheumatoid Arthritis Reference Center | Retrospective | 47 | Colombia | 2019 | 118 |
| Golimumab | RA | Subcutaneous Actemra | Hepatobiliary disorders | 189 | 3,67 | Janssen Pharmaceutical |  | 5151 | Japan | 2018 | 127 |
| Golimumab | RA | NS | Hepatobiliary disorders | 9 | 2,99 | 119 centers | Prospective | 301 | Japan | 2019 | 148 |
| Golimumab | RA | NS | Herpes zoster | 2 | 4,26 | Rheumatoid Arthritis Reference Center | Retrospective | 47 | Colombia | 2019 | 118 |
| Golimumab | RA | NS | Herpes zoster | 4 | 1,33 | 119 centers | Prospective | 301 | Japan | 2019 | 148 |
| Golimumab | RA | Subcutaneous Actemra | Immune system disorders | 3 | 0,06 | Janssen Pharmaceutical |  | 5140 | Japan | 2018 | 127 |
| Golimumab | RA | Subcutaneous Actemra | Infections and infestations | 375 | 7,3 | Janssen Pharmaceutical |  | 5137 | Japan | 2018 | 127 |
| Golimumab | RA | NS | Infections and infestations | 29 | 9,63 | 119 centers | Prospective | 301 | Japan | 2019 | 148 |
| Golimumab | RA | NS | Infections and infestations | 9 | 2,99 | 119 centers | Prospective | 301 | Japan | 2019 | 148 |
| Golimumab | RA | Subcutaneous Actemra | Injury, poisoning and procedural complications | 159 | 3,08 | Janssen Pharmaceutical |  | 5157 | Japan | 2018 | 127 |
| Golimumab | RA | NS | Interstitial lung disease | 2 | 0,66 | 119 centers | Prospective | 301 | Japan | 2019 | 148 |
| Golimumab | RA | NS | Liver function test abnormal | 11 | 3,65 | 119 centers | Prospective | 301 | Japan | 2019 | 148 |
| Golimumab | RA | NS | Malignancy | 4 | 1,33 | 119 centers | Prospective | 301 | Japan | 2019 | 148 |
| Golimumab | RA | Subcutaneous Actemra | Metabolism and nutrition disorders | 23 | 0,45 | Janssen Pharmaceutical |  | 5142 | Japan | 2018 | 127 |
| Golimumab | RA | Subcutaneous Actemra | Musculoskeletal and connective tissue disorders | 44 | 0,85 | Janssen Pharmaceutical |  | 5153 | Japan | 2018 | 127 |
| Golimumab | RA | Subcutaneous Actemra | Nervous system disorders | 32 | 0,62 | Janssen Pharmaceutical |  | 5144 | Japan | 2018 | 127 |
| Golimumab | RA | NS | Other | 3 | 6,38 | Rheumatoid Arthritis Reference Center | Retrospective | 47 | Colombia | 2019 | 118 |
| Golimumab | RA | NS | Pneumocystis jiroveci (carinii) Pneumonia | 4 | 1,33 | 119 centers | Prospective | 301 | Japan | 2019 | 148 |
| Golimumab | RA | NS | Pneumonia | 0 | 0 | Rheumatoid Arthritis Reference Center | Retrospective | 47 | Colombia | 2019 | 118 |
| Golimumab | RA | NS | Psoriasis | 0 | 0 | Rheumatoid Arthritis Reference Center | Retrospective | 47 | Colombia | 2019 | 118 |
| Golimumab | RA | Subcutaneous Actemra | Psychiatric disorders | 11 | 0,21 | Janssen Pharmaceutical |  | 5143 | Japan | 2018 | 127 |
| Golimumab | RA | NS | Rash | 0 | 0 | Rheumatoid Arthritis Reference Center | Retrospective | 47 | Colombia | 2019 | 118 |
| Golimumab | RA | Subcutaneous Actemra | Renal and urinary disorders | 20 | 0,39 | Janssen Pharmaceutical |  | 5154 | Japan | 2018 | 127 |
| Golimumab | RA | Subcutaneous Actemra | Reproductive system and breast disorders | 1 | 0,02 | Janssen Pharmaceutical |  | 5155 | Japan | 2018 | 127 |
| Golimumab | RA | Subcutaneous Actemra | Reproductive system and breast disorders | 3 | 0,06 | Janssen Pharmaceutical |  | 5159 | Japan | 2018 | 127 |
| Golimumab | RA | Subcutaneous Actemra | Respiratory, thoracic and mediastinal disorders | 75 | 1,46 | Janssen Pharmaceutical |  | 5149 | Japan | 2018 | 127 |
| Golimumab | RA | NS | Respiratory, thoracic and mediastinal disorders | 13 | 4,32 | 119 centers | Prospective | 301 | Japan | 2019 | 148 |
| Golimumab | RA | NS | Sepsis | 0 | 0 | Rheumatoid Arthritis Reference Center | Retrospective | 47 | Colombia | 2019 | 118 |
| Golimumab | RA | Subcutaneous Actemra | Skin and subcutaneous tissue disorders | 122 | 2,37 | Janssen Pharmaceutical |  | 5152 | Japan | 2018 | 127 |
| Golimumab | RA | NS | Stroke | 0 | 0 | Rheumatoid Arthritis Reference Center | Retrospective | 47 | Colombia | 2019 | 118 |
| Golimumab | RA | Subcutaneous Actemra | Surgical and medical procedures | 41 | 0,79 | Janssen Pharmaceutical |  | 5158 | Japan | 2018 | 127 |
| Golimumab | RA | NS | TB | 0 | 0 | Rheumatoid Arthritis Reference Center | Retrospective | 47 | Colombia | 2019 | 118 |
| Golimumab | RA | NS | TB | 0 | 0 | 119 centers | Prospective | 301 | Japan | 2019 | 148 |
| Golimumab | RA | NS | Therapeutic failure | 4 | 5,06 | Padre Billini Teaching Hospital | Ambispective | 79 | Dominican Republic | 2018 | 133 |
| Golimumab | RA | Subcutaneous Actemra | Vascular disorders | 16 | 0,31 | Janssen Pharmaceutical |  | 5148 | Japan | 2018 | 127 |
| Golimumab | RA | NS | White blood cell count decreased | 3 | 1 | 119 centers | Prospective | 301 | Japan | 2019 | 148 |
| Infliximab | AS | NS | Peripheral neuropathy | 1 | 1,3 | NS | Prospective | 77 | Greece | 2014 | 120 |
| Infliximab | RA | Remicade | Acne | 0 | 0 | Rheumatoid Arthritis Reference Center | Retrospective | 107 | Colombia | 2016 | 141 |
| Infliximab | RA | Remicade | Angioedema | 4 | 3,74 | Rheumatoid Arthritis Reference Center | Retrospective | 107 | Colombia | 2016 | 141 |
| Infliximab | RA | NS | Axonal polyneuropathy | 3 | NS | BIOGEAS registro 2007 | Prospective | NS | Spain | 2011 | 119 |
| Infliximab | RA | Remicade | Breast mass, under study | 0 | 0 | Rheumatoid Arthritis Reference Center | Retrospective | 107 | Colombia | 2016 | 141 |
| Infliximab | RA | CT-P13 | Bursitis | 2 | 1,27 | PLANETRA study extension | Prospective | 158 | International | 2017 | 152 |
| Infliximab | RA | NS | Candidiasis | 2 | 1,87 | Rheumatoid Arthritis Reference Center | Retrospective | 107 | Colombia | 2019 | 118 |
| Infliximab | RA | Remicade | Candidiasis | 2 | 1,87 | Rheumatoid Arthritis Reference Center | Retrospective | 107 | Colombia | 2016 | 141 |
| Infliximab | RA | NS | Cellulitis | 2 | 1,87 | Rheumatoid Arthritis Reference Center | Retrospective | 107 | Colombia | 2019 | 118 |
| Infliximab | RA | Remicade | Cellulitis | 2 | 1,87 | Rheumatoid Arthritis Reference Center | Retrospective | 107 | Colombia | 2016 | 141 |
| Infliximab | RA | Remicade | Cervical cancer | 0 | 0 | Rheumatoid Arthritis Reference Center | Retrospective | 107 | Colombia | 2016 | 141 |
| Infliximab | RA | Remicade | Chilblains | 0 | 0 | Rheumatoid Arthritis Reference Center | Retrospective | 107 | Colombia | 2016 | 141 |
| Infliximab | US | NS | ADA | 27,76 ± 17.13 | NS | Rheumatology centers | Prospective | 41* | Italia | 2017 | 112 |
| Infliximab | US | NS | ADA | 27.27 ± 17.28 | NS | Rheumatology centers | Prospective | 41* | Italia | 2017 | 112 |
| Infliximab | RA | NS | Dermatitis | 7 | 6,54 | Rheumatoid Arthritis Reference Center | Retrospective | 107 | Colombia | 2019 | 118 |
| Infliximab | RA | Remicade | Dermatitis | 25 | 23,36 | Rheumatoid Arthritis Reference Center | Retrospective | 107 | Colombia | 2016 | 141 |
| Infliximab | RA | NS | Dermatomycosis | 0 | 0 | Rheumatoid Arthritis Reference Center | Retrospective | 107 | Colombia | 2019 | 118 |
| Infliximab | RA | NS | Diarrhea | 2 | 1,87 | Rheumatoid Arthritis Reference Center | Retrospective | 107 | Colombia | 2019 | 118 |
| Infliximab | RA | Remicade | Diarrhea | 3 | 2,8 | Rheumatoid Arthritis Reference Center | Retrospective | 107 | Colombia | 2016 | 141 |
| Infliximab | RA | NS | Elevated blood pressure | 1 | 0,93 | Rheumatoid Arthritis Reference Center | Retrospective | 107 | Colombia | 2019 | 118 |
| Infliximab | RA | Remicade | Elevated blood pressure | 11 | 10,28 | Rheumatoid Arthritis Reference Center | Retrospective | 107 | Colombia | 2016 | 141 |
| Infliximab | RA | NS | Headache | 0 | 0 | Rheumatoid Arthritis Reference Center | Retrospective | 107 | Colombia | 2019 | 118 |
| Infliximab | RA | Remicade | Headache | 0 | 0 | Rheumatoid Arthritis Reference Center | Retrospective | 107 | Colombia | 2016 | 141 |
| Infliximab | RA | NS | Herpes zoster | 4 | 3,74 | Rheumatoid Arthritis Reference Center | Retrospective | 107 | Colombia | 2019 | 118 |
| Infliximab | RA | Remicade | Herpes zoster | 1 | 0,93 | Rheumatoid Arthritis Reference Center | Retrospective | 107 | Colombia | 2016 | 141 |
| Infliximab | RA | NS | Hospitalization because of an infection | 53 | - | National Registry of Biological Treatment in Finland and hospital records of the Central Hospital of Central Finland | Prospective | NS | Finland | 2015 | 108 |
| Infliximab | RA | NS | Infection treatment suspension | 20 | 4,85 | REAL | Prospective | 412 | Japan | 2012 | 140 |
| Infliximab | RA | NS | Intra-abdominal infections | 3 | 0,36 | GISEA | Ambispective | 837 | Italia | 2012 | 111 |
| Infliximab | RA | Remicade | Leukopenia | 0 | 0 | Rheumatoid Arthritis Reference Center | Retrospective | 107 | Colombia | 2016 | 141 |
| Infliximab | RA | NS | Lewis – Sumner syndrome (MADSAM neuropathy) | 2 | NS | BIOGEAS registro 2008 | Prospective | NS | Spain | 2011 | 119 |
| Infliximab | RA | NS | Listeria monocytogenesis meningitis | 2 | NS | FDA Adverse Event Reporting System | Retrospective | NS | United States | 2003 | 146 |
| Infliximab | RA | CT-P13 | Liver function test abnormal | 1 | 0,63 | PLANETRA study extension | Prospective | 158 | International | 2017 | 152 |
| Infliximab | RA | Remicade | Local reaction | 0 | 0 | Rheumatoid Arthritis Reference Center | Retrospective | 107 | Colombia | 2016 | 141 |
| Infliximab | RA | NS | Lower respiratory tract infection | 15 | 1,79 | GISEA | Ambispective | 837 | Italia | 2012 | 111 |
| Infliximab | RA | CT-P13 | Lower respiratory tract infection | 4 | 2,53 | PLANETRA study extension | Prospective | 158 | International | 2017 | 152 |
| Infliximab | RA | Remicade | Lupus like | 0 | 0 | Rheumatoid Arthritis Reference Center | Retrospective | 107 | Colombia | 2016 | 141 |
| Infliximab | RA | NS | Medical and surgical procedures | NS | 0 | Postmarketing study | Prospective | 5000 | Japan | 2008 | 147 |
| Infliximab | RA | NS | Meningitis and sepsis due to Listeria monocytogenesis | 1 | NS | FDA Adverse Event Reporting System | Retrospective | NS | United States | 2003 | 146 |
| Infliximab | RA | NS | Miller – Fisher syndrome | 1 | NS | BIOGEAS registro 2009 | Prospective | NS | Spain | 2011 | 119 |
| Infliximab | RA | NS | Multiple monoeuritis | 2 | NS | BIOGEAS registro 2006 | Prospective | NS | Spain | 2011 | 119 |
| Infliximab | RA | Remicade | Myalgia | 2 | 1,87 | Rheumatoid Arthritis Reference Center | Retrospective | 107 | Colombia | 2016 | 141 |
| Infliximab | RA | NS | Ocular infection | 5 | 0,6 | GISEA | Ambispective | 837 | Italia | 2012 | 111 |
| Infliximab | RA | NS | Osteoarticular infection | 6 | 0,72 | GISEA | Ambispective | 837 | Italia | 2012 | 111 |
| Infliximab | RA | NS | Other | 13 | 12,15 | Rheumatoid Arthritis Reference Center | Retrospective | 107 | Colombia | 2019 | 118 |
| Infliximab | RA | Remicade | Other | 4 | 3,74 | Rheumatoid Arthritis Reference Center | Retrospective | 107 | Colombia | 2016 | 141 |
| Infliximab | RA | Remicade | Paresthesia | 0 | 0 | Rheumatoid Arthritis Reference Center | Retrospective | 107 | Colombia | 2016 | 141 |
| Infliximab | RA | NS | Pneumonia | 3 | 2,8 | Rheumatoid Arthritis Reference Center | Retrospective | 107 | Colombia | 2019 | 118 |
| Infliximab | RA | Remicade | Pneumonia | 2 | 1,87 | Rheumatoid Arthritis Reference Center | Retrospective | 107 | Colombia | 2016 | 141 |
| Infliximab | RA | Remicade | Polyarthralgias increase | 4 | 3,74 | Rheumatoid Arthritis Reference Center | Retrospective | 107 | Colombia | 2016 | 141 |
| Infliximab | RA | NS | Psoriasis | 4 | 3,74 | Rheumatoid Arthritis Reference Center | Retrospective | 107 | Colombia | 2019 | 118 |
| Infliximab | RA | Remicade | Psoriasis | 4 | 3,74 | Rheumatoid Arthritis Reference Center | Retrospective | 107 | Colombia | 2016 | 141 |
| Infliximab | RA | NS | Rash | 19 | 17,76 | Rheumatoid Arthritis Reference Center | Retrospective | 107 | Colombia | 2019 | 118 |
| Infliximab | RA | CT-P13 | Reactions associated infusion | 11 | 6,96 | PLANETRA study extension | Prospective | 158 | International | 2017 | 152 |
| Infliximab | RA | NS | Sepsis | 2 | 0,24 | GISEA | Ambispective | 837 | Italia | 2012 | 111 |
| Infliximab | RA | NS | Sepsis | 0 | 0 | Rheumatoid Arthritis Reference Center | Retrospective | 107 | Colombia | 2019 | 118 |
| Infliximab | RA | NS | Sepsis due to Listeria monocytogenesis | 4 | NS | FDA Adverse Event Reporting System | Retrospective | NS | United States, Germany and Canada | 2003 | 146 |
| Infliximab | RA | Remicade | Septic arthritis | 2 | 1,87 | Rheumatoid Arthritis Reference Center | Retrospective | 107 | Colombia | 2016 | 141 |
| Infliximab | RA | NS | Septic arthritis Listeria monocytogenesis | 1 | NS | FDA Adverse Event Reporting System | Retrospective | NS | France | 2003 | 146 |
| Infliximab | RA | NS | Serius infections | 151 | 18,04 | GISEA | Ambispective | 837 | Italia | 2012 | 111 |
| Infliximab | JIA | Remicade | Allergic reactions | 6 | 15,79 | Switzerland database | Prospective | 38 | Suiza | 2010 | 143 |
| Infliximab | JIA | NS | Antibody to double-stranded DNA | 4 | 6,56 | NS | Prospective | 61 | North America, Latin America and Europe | 2010 | 138 |
| Infliximab | JIA | NS | Antinuclear antibodies | 15 | 25,86 | NS | Prospective | 58 | North America, Latin America and Europe | 2010 | 138 |
| Infliximab | JIA | NS | Antinuclear antibodies | 1 | 1,28 | NS | Prospective | 78 | North America, Latin America and Europe | 2010 | 138 |
| Infliximab | JIA | NS | Bronchitis | 8 | 10,26 | NS | Prospective | 78 | North America, Latin America and Europe | 2010 | 138 |
| Infliximab | JIA | NS | Discontinuation by Anaphylactoid reaction | 1 | 1,28 | NS | Prospective | 78 | North America, Latin America and Europe | 2010 | 138 |
| Infliximab | JIA | NS | Discontinuation by Chills | 1 | 1,28 | NS | Prospective | 78 | North America, Latin America and Europe | 2010 | 138 |
| Infliximab | JIA | NS | Discontinuation by Coughing | 1 | 1,28 | NS | Prospective | 78 | North America, Latin America and Europe | 2010 | 138 |
| Infliximab | JIA | NS | Discontinuation by fever | 1 | 1,28 | NS | Prospective | 78 | North America, Latin America and Europe | 2010 | 138 |
| Infliximab | JIA | NS | Discontinuation by Infusion syndrome | 5 | 6,41 | NS | Prospective | 78 | North America, Latin America and Europe | 2010 | 138 |
| Infliximab | JIA | NS | Discontinuation by pneumonia | 2 | 2,56 | NS | Prospective | 78 | North America, Latin America and Europe | 2010 | 138 |
| Infliximab | JIA | NS | Discontinuation by Urticaria | 1 | 1,28 | NS | Prospective | 78 | North America, Latin America and Europe | 2010 | 138 |
| Infliximab | JIA | NS | Discontinuation by uveitis | 1 | 1,28 | NS | Prospective | 78 | North America, Latin America and Europe | 2010 | 138 |
| Infliximab | JIA | NS | Discontinuation by vomiting | 1 | 1,28 | NS | Prospective | 78 | North America, Latin America and Europe | 2010 | 138 |
| Infliximab | JIA | NS | Fever | 8 | 10,26 | NS | Prospective | 78 | North America, Latin America and Europe | 2010 | 138 |
| Infliximab | JIA | NS | Fever | 18 | 23,08 | NS | Prospective | 78 | North America, Latin America and Europe | 2010 | 138 |
| Infliximab | JIA | NS | Headache | 19 | 24,36 | NS | Prospective | 78 | North America, Latin America and Europe | 2010 | 138 |
| Infliximab | JIA | NS | Infections | 57 | 73,08 | NS | Prospective | 78 | North America, Latin America and Europe | 2010 | 138 |
| Infliximab | JIA | Remicade | Infections | 8 | 21,05 | Switzerland database | Prospective | 38 | Suiza | 2010 | 143 |
| Infliximab | JIA | NS | Infusion reactions | 25 | 32,05 | NS | Prospective | 78 | North America, Latin America and Europe | 2010 | 138 |
| Infliximab | JIA | NS | Negative antibodies | 5 | 22,73 | NS | Prospective | 22 | North America, Latin America and Europe | 2010 | 138 |
| Infliximab | JIA | NS | Pharyngitis | 30 | 38,46 | NS | Prospective | 78 | North America, Latin America and Europe | 2010 | 138 |
| Infliximab | JIA | NS | Pharyngitis | 23 | 29,49 | NS | Prospective | 78 | North America, Latin America and Europe | 2010 | 138 |
| Infliximab | JIA | NS | Positive antibodies | 15 | 57,69 | NS | Prospective | 26 | North America, Latin America and Europe | 2010 | 138 |
| Infliximab | JIA | NS | Possible delayed hypersensitivity reaction | 0 | 0 | NS | Prospective | 78 | North America, Latin America and Europe | 2010 | 138 |
| Infliximab | JIA | Remicade | Psorasiform rash and alopecia | 1 | 2,63 | Switzerland database | Prospective | 38 | Suiza | 2010 | 143 |
| Infliximab | JIA | NS | Rhinitis | 18 | 23,08 | NS | Prospective | 78 | North America, Latin America and Europe | 2010 | 138 |
| Infliximab | JIA | NS | Rhinitis | 12 | 15,38 | NS | Prospective | 78 | North America, Latin America and Europe | 2010 | 138 |
| Infliximab | JIA | NS | Serious adverse events | 17 | 21,79 | NS | Prospective | 78 | North America, Latin America and Europe | 2010 | 138 |
| Infliximab | JIA | NS | Serious infusion reactions | 2 | 2,56 | NS | Prospective | 78 | North America, Latin America and Europe | 2010 | 138 |
| Infliximab | RA | NS | Skin and soft tissue infections | 10 | 1,19 | GISEA | Ambispective | 837 | Italia | 2012 | 111 |
| Infliximab | RA | NS | Stroke | 1 | 0,93 | Rheumatoid Arthritis Reference Center | Retrospective | 107 | Colombia | 2019 | 118 |
| Infliximab | RA | Remicade | Stroke | 2 | 1,87 | Rheumatoid Arthritis Reference Center | Retrospective | 107 | Colombia | 2016 | 140 |
| Infliximab | RA | NS | Suspension of treatment due to infusion reaction | 6 | 1,46 | REAL | Prospective | 412 | Japan | 2012 | 140 |
| Infliximab | RA | NS | Suspension of treatment due to loss of efficacy | 68 | 16,5 | REAL | Prospective | 412 | Japan | 2012 | 140 |
| Infliximab | RA | NS | Suspension of treatment for adverse events | 57 | 13,83 | REAL | Prospective | 412 | Japan | 2012 | 140 |
| Infliximab | RA | NS | Suspension of treatment for cardiovascular disease | 2 | 0,49 | REAL | Prospective | 412 | Japan | 2012 | 140 |
| Infliximab | JIA | NS | Upper respiratory tract infection | 31 | 39,74 | NS | Prospective | 78 | North America, Latin America and Europe | 2010 | 138 |
| Infliximab | JIA | NS | Upper respiratory tract infection | 25 | 32,05 | NS | Prospective | 78 | North America, Latin America and Europe | 2010 | 138 |
| Infliximab | JIA | NS | Vomiting | 17 | 21,79 | NS | Prospective | 78 | North America, Latin America and Europe | 2010 | 138 |
| Infliximab | RA | NS | Suspension of treatment for malignancy | 7 | 1,7 | REAL | Prospective | 412 | Japan | 2012 | 140 |
| Infliximab | RA | NS | Suspension of treatment for non-infectious lung disease | 7 | 1,7 | REAL | Prospective | 412 | Japan | 2012 | 140 |
| Infliximab | RA | Remicade | Tachycardia | 2 | 1,87 | Rheumatoid Arthritis Reference Center | Retrospective | 107 | Colombia | 2016 | 141 |
| Infliximab | RA | NS | TB | 6 | 0,72 | GISEA | Ambispective | 837 | Italia | 2012 | 111 |
| Infliximab | RA | NS | TB | 0 | 0 | Rheumatoid Arthritis Reference Center | Retrospective | 107 | Colombia | 2019 | 118 |
| Infliximab | RA | Remicade | TB | 0 | 0 | Rheumatoid Arthritis Reference Center | Retrospective | 107 | Colombia | 2016 | 141 |
| Infliximab | RA | CT-P13 | TB | 9 | 5,7 | PLANETRA study extension | Prospective | 158 | International | 2017 | 152 |
| Infliximab | RA | NS | Upper respiratory tract infection | 67 | 8 | GISEA | Ambispective | 837 | Italia | 2012 | 111 |
| Infliximab | RA | CT-P13 | Upper respiratory tract infection | 6 | 3,8 | PLANETRA study extension | Prospective | 158 | International | 2017 | 152 |
| Infliximab | RA | NS | UTI | 28 | 3,35 | GISEA | Ambispective | 837 | Italia | 2012 | 111 |
| Infliximab | RA | Remicade | UTI | 0 | 0 | Rheumatoid Arthritis Reference Center | Retrospective | 107 | Colombia | 2016 | 141 |
| Infliximab | RA | CT-P13 | UTI | 2 | 1,27 | PLANETRA study extension | Prospective | 158 | International | 2017 | 152 |
| Infliximab | RA, AS and PsA | NS | Neutropenia | 14 | 20,3 | Rheumatology Department's database at the Derbyshire Royal Infirmary | Retrospective | 69 | United Kingdom | 2010 | 124 |
| Infliximab, etanercept, adalimumab y otros | RA + SP + IBD | NS | Chronic inflammatory demyelinating polyneuropathy | 5 | NS | BIOGEAS registro 2011 | Prospective | NS | Spain | 2011 | 119 |
| Infliximab, etanercept, adalimumab y otros | RA + SP + IBD | NS | Guillain Barre syndrome | 14 | NS | BIOGEAS registro 2009 | Prospective | NS | Spain | 2011 | 119 |
| Infliximab, etanercept, adalimumab y otros | RA + SP + IBD | NS | Motor neuropathy with muliple driving block | 10 | NS | BIOGEAS registro 2010 | Prospective | NS | Spain | 2011 | 119 |
| Infliximab, etanercept, adalimumab y otros | RA + SP + IBD | NS | Multiple sclerosis | 28 | NS | BIOGEAS registro 2008 | Prospective | NS | Spain | 2011 | 119 |
| Infliximab, etanercept, adalimumab y otros | RA + SP + IBD | NS | Optic neuritis | 116 | NS | BIOGEAS registro 2007 | Prospective | NS | Spain | 2011 | 119 |
| Rituximab | RA | NS | Exacerbations RA | 83 | 2,3 | Global Clinical Trial Program | Prospective | 3595 | International | 2015 | 149 |
| Rituximab | RA | NS | Hospitalization because of an infection | 37 | 8,45 | National Registry of Biological Treatment in Finland and hospital records of the Central Hospital of Central Finland | Prospective | 438 | Finland | 2015 | 108 |
| Rituximab | RA | NS | Osteoarthritis | 55 | 1,5 | Global Clinical Trial Program | Prospective | 3595 | International | 2015 | 149 |
| Rituximab | RA | NS | Pneumonia | 74 | 2,1 | Global Clinical Trial Program | Prospective | 3595 | International | 2015 | 149 |
| Rituximab | RA | NS | Progressive multifocal leukoencephalopathy | 9 | 0 | NS | Prospective | 351396 | International | 2018 | 113 |
| Rituximab | RA | NS | Relapses | 62 | 1,7 | Global Clinical Trial Program | Prospective | 3595 | International | 2015 | 149 |
| Rituximab | RA | NS | Therapeutic failure | 4 | 6,06 | Padre Billini Teaching Hospital | Ambispective | 66 | Dominican Republic | 2018 | 133 |
| Tocilizumab | JIA | Actemra | Altered paraclinics | 44 | 10,55 | Chugai Pharmaceutical | Prospective | 417 | Japan | 2016 | 151 |
| Tocilizumab | JIA | Actemra | Blood and lymphatic system disorders | 46 | 11,03 | Chugai Pharmaceutical | Prospective | 417 | Japan | 2016 | 151 |
| Tocilizumab | JIA | Actemra | Bronchitis | 24 | 5,76 | Chugai Pharmaceutical | Prospective | 417 | Japan | 2016 | 151 |
| Tocilizumab | JIA | Actemra | Cardiac disorders | 3 | 0,72 | Chugai Pharmaceutical | Prospective | 417 | Japan | 2016 | 151 |
| Tocilizumab | JIA | Actemra | Eye disorders | 12 | 2,88 | Chugai Pharmaceutical | Prospective | 417 | Japan | 2016 | 151 |
| Tocilizumab | JIA | Actemra | Gastroenteritis | 30 | 7,19 | Chugai Pharmaceutical | Prospective | 417 | Japan | 2016 | 151 |
| Tocilizumab | JIA | Actemra | Gastrointestinal disorders | 42 | 10,07 | Chugai Pharmaceutical | Prospective | 417 | Japan | 2016 | 151 |
| Tocilizumab | JIA | Actemra | General disorders and administration site conditions | 24 | 5,76 | Chugai Pharmaceutical | Prospective | 417 | Japan | 2016 | 151 |
| Tocilizumab | JIA | Actemra | Haematophagic histiocytosis | 24 | 5,76 | Chugai Pharmaceutical | Prospective | 417 | Japan | 2016 | 151 |
| Tocilizumab | JIA | Actemra | Hepatobiliary disorders | 35 | 8,39 | Chugai Pharmaceutical | Prospective | 417 | Japan | 2016 | 151 |
| Tocilizumab | JIA | Actemra | Immune system disorders | 5 | 1,2 | Chugai Pharmaceutical | Prospective | 417 | Japan | 2016 | 151 |
| Tocilizumab | JIA | Actemra | Infections and infestations | 171 | 41,01 | Chugai Pharmaceutical | Prospective | 417 | Japan | 2016 | 151 |
| Tocilizumab | JIA | Actemra | Infections and infestations | 55 | 13,19 | Chugai Pharmaceutical | Prospective | 417 | Japan | 2016 | 151 |
| Tocilizumab | JIA | Actemra | Influenza | 30 | 7,19 | Chugai Pharmaceutical | Prospective | 417 | Japan | 2016 | 151 |
| Tocilizumab | JIA | Actemra | Injury, poisoning and procedural complications | 27 | 6,47 | Chugai Pharmaceutical | Prospective | 417 | Japan | 2016 | 151 |
| Tocilizumab | JIA | Actemra | Liver function test abnormal | 29 | 6,95 | Chugai Pharmaceutical | Prospective | 417 | Japan | 2016 | 151 |
| Tocilizumab | JIA | Actemra | Medical and surgical procedures | 1 | 0,24 | Chugai Pharmaceutical | Prospective | 417 | Japan | 2016 | 151 |
| Tocilizumab | JIA | Actemra | Metabolism and nutrition disorders | 12 | 2,88 | Chugai Pharmaceutical | Prospective | 417 | Japan | 2016 | 151 |
| Tocilizumab | JIA | Actemra | Musculoskeletal and connective tissue disorders | 59 | 14,15 | Chugai Pharmaceutical | Prospective | 417 | Japan | 2016 | 151 |
| Tocilizumab | JIA | Actemra | Nasopharyngitis | 22 | 5,28 | Chugai Pharmaceutical | Prospective | 417 | Japan | 2016 | 151 |
| Tocilizumab | JIA | Actemra | Nervous system disorders | 12 | 2,88 | Chugai Pharmaceutical | Prospective | 417 | Japan | 2016 | 151 |
| Tocilizumab | JIA | Actemra | Pharyngitis | 22 | 5,28 | Chugai Pharmaceutical | Prospective | 417 | Japan | 2016 | 151 |
| Tocilizumab | JIA | Actemra | Renal and urinary disorders | 7 | 1,68 | Chugai Pharmaceutical | Prospective | 417 | Japan | 2016 | 151 |
| Tocilizumab | JIA | Actemra | Respiratory, thoracic and mediastinal disorders | 82 | 19,66 | Chugai Pharmaceutical | Prospective | 417 | Japan | 2016 | 151 |
| Tocilizumab | JIA | Actemra | Skin and subcutaneous tissue disorders | 38 | 9,11 | Chugai Pharmaceutical | Prospective | 417 | Japan | 2016 | 151 |
| Tocilizumab | JIA | Actemra | Upper respiratory tract inflammation | 71 | 17,03 | Chugai Pharmaceutical | Prospective | 417 | Japan | 2016 | 151 |
| Tocilizumab | JIA | Actemra | Vascular disorders | 14 | 3,36 | Chugai Pharmaceutical | Prospective | 417 | Japan | 2016 | 151 |
| Tocilizumab | RA | NS | Suspension of treatment due to infusion reaction | 0 | 0 | REAL | Prospective | 168 | Japan | 2012 | 140 |
| Tocilizumab | RA | NS | >2-3 ULN of ALT | 8 | 5,3 | 76 centers | Prospective | 151 | 15 countries australia, europe, north and south america. | 2013 | 122 |
| Tocilizumab | RA | NS | >2-3 ULN of AST | 3 | 1,95 | 76 centers | Prospective | 154 | 15 countries australia, europe, north and south america. | 2013 | 122 |
| Tocilizumab | RA | NS | >3-5 ULN of ALT | 2 | 1,32 | 76 centers | Prospective | 151 | 15 countries australia, europe, north and south america. | 2013 | 122 |
| Tocilizumab | RA | Subcutaneous Actemra | >3-5 ULN of ALT | 6 | 1,7 | Multicentric | Prospective | 353 | 22 countries | 2018 | 121 |
| Tocilizumab | RA | NS | >3-5 ULN of AST | 2 | 1,3 | 76 centers | Prospective | 154 | 15 countries australia, europe, north and south america. | 2013 | 122 |
| Tocilizumab | RA | Subcutaneous Actemra | >3-5 ULN of AST | 2 | 0,57 | Multicentric | Prospective | 353 | 22 countries | 2018 | 121 |
| Tocilizumab | RA | NS | Abdominal pain | 2 | 4 | 6 Specialized centers sponsored by Roche Products | Prospective | 50 | Argentina | 2017 | 135 |
| Tocilizumab | RA | Subcutaneous Actemra | ADA | 7 | 1,98 | Multicentric | Prospective | 353 | 22 countries | 2018 | 121 |
| Tocilizumab | RA | NS | Allergy treatment suspension | 6 | 3,57 | REAL | Prospective | 168 | Japan | 2012 | 140 |
| Tocilizumab | RA | Subcutaneous Actemra | AR Reproductive system | 1 | 0,46 | Multicentric | Prospective | 219 | Japan | 2018 | 110 |
| Tocilizumab | RA | NS | AR Skin | 3 | 6 | 6 Specialized centers sponsored by Roche Products | Prospective | 50 | Argentina | 2017 | 135 |
| Tocilizumab | RA | Actemra | Arthritis bacterial | 51 | 0,67 | Database of adverse events Chugai Pharmaceutical Co | Prospective | 7653 | Japan | 2018 | 109 |
| Tocilizumab | RA | Actemra | Arthritis infective | 17 | 0,22 | Database of adverse events Chugai Pharmaceutical Co | Prospective | 7653 | Japan | 2018 | 109 |
| Tocilizumab | RA | Actemra | Bacterial pneumonia | 44 | 0,57 | Database of adverse events Chugai Pharmaceutical Co | Prospective | 7653 | Japan | 2018 | 109 |
| Tocilizumab | RA | NS | Blood and lymphatic system disorders | 64 | 1,65 | Chugai Pharmaceutical |  | 3881 | Japan | 2011 | 139 |
| Tocilizumab | RA | NS | Bronchitis | 1 | 0,62 | 76 centers | Prospective | 162 | 15 Australasia, Europe, North and South america countries | 2013 | 122 |
| Tocilizumab | RA | Actemra | Cellulitis | 158 | 2,06 | Database of adverse events Chugai Pharmaceutical Co | Prospective | 7653 | Japan | 2018 | 109 |
| Tocilizumab | RA | NS | Cellulitis | 58 | 0,73 | Chugai Pharmaceutical | Prospective | 7901 | Japan | 2014 | 131 |
| Tocilizumab | RA | NS | Death | 2 | 1,23 | 76 centers | Prospective | 162 | 15 Australasia, Europe, North and South america countries | 2013 | 122 |
| Tocilizumab | RA | Actemra | Diverticulitis | 43 | 0,56 | Database of adverse events Chugai Pharmaceutical Co | Prospective | 7653 | Japan | 2018 | 109 |
| Tocilizumab | RA | NS | Dyslipidemia /hypertriglyceridemia | 2 | 4 | 6 Specialized centers sponsored by Roche Products | Prospective | 50 | Argentina | 2017 | 135 |
| Tocilizumab | RA | Subcutaneous Actemra | Endocrine disorders | 1 | 0,46 | Multicentric | Prospective | 219 | Japan | 2018 | 110 |
| Tocilizumab | RA | NS | Eye disorders | 20 | 0,52 | Chugai Pharmaceutical |  | 3881 | Japan | 2011 | 139 |
| Tocilizumab | RA | Subcutaneous Actemra | Gastrointestinal disorders | 2 | 0,91 | Multicentric | Prospective | 219 | Japan | 2018 | 110 |
| Tocilizumab | RA | NS | Gastrointestinal infection | 7 | 0,83 | FIRST Bio study | Prospective | 843 | Japan | 2017 | 125 |
| Tocilizumab | RA | Subcutaneous Actemra | General disorders and administration site conditions | 10 | 4,57 | Multicentric | Prospective | 219 | Japan | 2018 | 110 |
| Tocilizumab | RA | Subcutaneous Actemra | Grade 1 Neutropenia | 82 | 23,23 | Multicentric | Prospective | 353 | 22 countries | 2018 | 121 |
| Tocilizumab | RA | Subcutaneous Actemra | Grade 2 Neutropenia | 54 | 15,3 | Multicentric | Prospective | 353 | 22 countries | 2018 | 121 |
| Tocilizumab | RA | Subcutaneous Actemra | Grade 3 Neutropenia | 19 | 5,38 | Multicentric | Prospective | 353 | 22 countries | 2018 | 121 |
| Tocilizumab | RA | NS | Hematoma infection | 1 | 0,62 | 76 centers | Prospective | 162 | 15 Australasia, Europe, North and South america countries | 2013 | 122 |
| Tocilizumab | RA | Subcutaneous Actemra | Hepatobiliary disorders | 9 | 4,11 | Multicentric | Prospective | 219 | Japan | 2018 | 110 |
| Tocilizumab | RA | Actemra | Herpes zoster | 56 | 0,73 | Database of adverse events Chugai Pharmaceutical Co | Prospective | 7653 | Japan | 2018 | 109 |
| Tocilizumab | RA | NS | Infection treatment suspension | 8 | 4,76 | REAL | Prospective | 168 | Japan | 2012 | 140 |
| Tocilizumab | RA | NS | Infections | 113 | 69,75 | 76 centers | Prospective | 162 | 15 Australasia, Europe, North and South america countries | 2013 | 122 |
| Tocilizumab | RA | NS | Infections | 5 | 10 | 6 Specialized centers sponsored by Roche Products | Prospective | 50 | Argentina | 2017 | 135 |
| Tocilizumab | RA | Subcutaneous Actemra | Infections and infestations | 6 | 1,7 | Multicentric | Prospective | 353 | 22 countries | 2018 | 121 |
| Tocilizumab | RA | Actemra | Infectious pleural effusion | 24 | 0,31 | Database of adverse events Chugai Pharmaceutical Co | Prospective | 7653 | Japan | 2018 | 109 |
| Tocilizumab | RA | NS | Infectious tenosynovitis | 1 | 0,62 | 76 centers | Prospective | 162 | 15 Australasia, Europe, North and South america countries | 2013 | 122 |
| Tocilizumab | RA | Subcutaneous Actemra | Leukopenia | 2 | 0,91 | Multicentric | Prospective | 219 | Japan | 2018 | 110 |
| Tocilizumab | RA | NS | Liver function test abnormal | 3 | 6 | 6 Specialized centers sponsored by Roche Products | Prospective | 50 | Argentina | 2017 | 135 |
| Tocilizumab | RA | NS | Metabolic / dyslipidemia | 4 | 8 | 6 Specialized centers sponsored by Roche Products | Prospective | 50 | Argentina | 2017 | 135 |
| Tocilizumab | RA | Subcutaneous Actemra | Metabolism and nutrition disorders | 4 | 1,83 | Multicentric | Prospective | 219 | Japan | 2018 | 110 |
| Tocilizumab | RA | Subcutaneous Actemra | Musculoskeletal and connective tissue disorders | 9 | 4,11 | Multicentric | Prospective | 219 | Japan | 2018 | 110 |
| Tocilizumab | RA | NS | Myocardial infarction or acute coronary syndrome | 2 | 1,23 | 76 centers | Prospective | 162 | 15 Australasia, Europe, North and South america countries | 2013 | 122 |
| Tocilizumab | RA | Subcutaneous Actemra | Nasopharyngitis | 2 | 0,91 | Multicentric | Prospective | 219 | Japan | 2018 | 110 |
| Tocilizumab | RA | Subcutaneous Actemra | Neoplasms | 1 | 0,46 | Multicentric | Prospective | 219 | Japan | 2018 | 110 |
| Tocilizumab | RA | Subcutaneous Actemra | Nervous system disorders | 3 | 1,37 | Multicentric | Prospective | 219 | Japan | 2018 | 110 |
| Tocilizumab | RA | NS | NonTB mycobacterial infection | 2 | 0,24 | FIRST Bio study | Prospective | 841 | Japan | 2017 | 125 |
| Tocilizumab | RA | Actemra | NonTB mycobacterial infection | 55 | 0,72 | Database of adverse events Chugai Pharmaceutical Co | Prospective | 7653 | Japan | 2018 | 109 |
| Tocilizumab | RA | Actemra | Other gastrointestinal infections | 92 | 1,2 | Database of adverse events Chugai Pharmaceutical Co | Prospective | 7653 | Japan | 2018 | 109 |
| Tocilizumab | RA | Subcutaneous Actemra | Other infections and infestations | 6 | 2,74 | Multicentric | Prospective | 219 | Japan | 2018 | 110 |
| Tocilizumab | RA | Actemra | Other skin infections | 98 | 1,28 | Database of adverse events Chugai Pharmaceutical Co | Prospective | 7653 | Japan | 2018 | 109 |
| Tocilizumab | RA | NS | PCR elevation | 1 | 2 | 6 Specialized centers sponsored by Roche Products | Prospective | 50 | Argentina | 2017 | 135 |
| Tocilizumab | RA | Actemra | Peritonitis | 48 | 0,63 | Database of adverse events Chugai Pharmaceutical Co | Prospective | 7653 | Japan | 2018 | 109 |
| Tocilizumab | RA | Actemra | Pneumococcal pneumonia | 25 | 0,33 | Database of adverse events Chugai Pharmaceutical Co | Prospective | 7653 | Japan | 2018 | 109 |
| Tocilizumab | RA | Actemra | Pneumocystis jiroveci (carinii) Pneumonia | 59 | 0,77 | Database of adverse events Chugai Pharmaceutical Co | Prospective | 7653 | Japan | 2018 | 109 |
| Tocilizumab | RA | Subcutaneous Actemra | Pneumonia | 2 | 0,91 | Multicentric | Prospective | 219 | Japan | 2018 | 110 |
| Tocilizumab | RA | Actemra | Pneumonia not specified | 163 | 2,13 | Database of adverse events Chugai Pharmaceutical Co | Prospective | 7653 | Japan | 2018 | 109 |
| Tocilizumab | RA | NS | Postoperative abscess | 1 | 0,62 | 76 centers | Prospective | 162 | 15 Australasia, Europe, North and South america countries | 2013 | 122 |
| Tocilizumab | RA | Subcutaneous Actemra | Psychiatric disorders | 1 | 0,46 | Multicentric | Prospective | 219 | Japan | 2018 | 110 |
| Tocilizumab | RA | Subcutaneous Actemra | Respiratory, thoracic and mediastinal disorders | 10 | 4,57 | Multicentric | Prospective | 219 | Japan | 2018 | 110 |
| Tocilizumab | RA | Actemra | Sepsis with or without shock | 137 | 1,79 | Database of adverse events Chugai Pharmaceutical Co | Prospective | 7653 | Japan | 2018 | 109 |
| Tocilizumab | RA | Actemra | Septic shock | 36 | 0,47 | Database of adverse events Chugai Pharmaceutical Co | Prospective | 7653 | Japan | 2018 | 109 |
| Tocilizumab | RA | Subcutaneous Actemra | Serious adverse events | 29 | 8,22 | Multicentric | Prospective | 353 | 22 countries | 2018 | 121 |
| Tocilizumab | RA | Subcutaneous Actemra | Serious gastrointestinal perforations | 1 | 0,28 | Multicentric | Prospective | 353 | 22 countries | 2018 | 121 |
| Tocilizumab | RA | Subcutaneous Actemra | Serious hypersensitivity reactions during the first 24 hours | 2 | 0,57 | Multicentric | Prospective | 353 | 22 countries | 2018 | 121 |
| Tocilizumab | RA | NS | Serius infections | 6 | 3,7 | 76 centers | Prospective | 162 | 15 Australasia, Europe, North and South america countries | 2013 | 122 |
| Tocilizumab | RA | Subcutaneous Actemra | Skin and subcutaneous tissue disorders | 8 | 3,65 | Multicentric | Prospective | 219 | Japan | 2018 | 110 |
| Tocilizumab | RA | NS | Stroke | 1 | 0,62 | 76 centers | Prospective | 162 | 15 Australasia, Europe, North and South america countries | 2013 | 122 |
| Tocilizumab | RA | Actemra | Subcutaneous abscess | 17 | 0,22 | Database of adverse events Chugai Pharmaceutical Co | Prospective | 7653 | Japan | 2018 | 109 |
| Tocilizumab | RA | NS | Suspension of treatment due to loss of efficacy | 23 | 13,69 | REAL | Prospective | 168 | Japan | 2012 | 140 |
| Tocilizumab | RA | NS | Suspension of treatment for adverse events | 23 | 13,69 | REAL | Prospective | 168 | Japan | 2012 | 140 |
| Tocilizumab | RA | NS | Suspension of treatment for cardiovascular disease | 2 | 1,19 | REAL | Prospective | 168 | Japan | 2012 | 140 |
| Tocilizumab | RA | NS | Suspension of treatment for malignancy | 1 | 0,6 | REAL | Prospective | 168 | Japan | 2012 | 140 |
| Tocilizumab | RA | NS | Suspension of treatment for non-infectious lung disease | 3 | 1,79 | REAL | Prospective | 168 | Japan | 2012 | 140 |
| Tocilizumab | RA | NS | Therapeutic failure | 8 | 1,81 | Padre Billini Teaching Hospital | Ambispective | 443 | Dominican Republic | 2018 | 133 |
| Tocilizumab | RA | NS | Transaminasemia | 39 | 8,8 | Padre Billini Teaching Hospital | Ambispective | 443 | Dominican Republic | 2018 | 133 |
| Tocilizumab | RA | NS | UTI | 1 | 0,12 | FIRST Bio study | Prospective | 842 | Japan | 2017 | 125 |
| Tocilizumab | RA | NS | Vestibular neuronitis | 1 | 0,62 | 76 centers | Prospective | 162 | 15 Australasia, Europe, North and South america countries | 2013 | 122 |
