# Supplementary material for: Pharmacovigilance of Biopharmaceuticals in Rheumatic Diseases, Adverse Events, Evolution, and Perspective: An Overview
Source: Biomedicines. 2020 Aug 23;8(9):303. doi: 10.3390/biomedicines8090303 (PMC7555940; doi:10.3390/biomedicines8090303)
Supplement: Supplementary file 1 [file biomedicines-08-00303-s001.zip › Tablas Suplementarias/Table S6_1000 patients-year.docx]

**Table S6. Adverse biotherapeutic events in rheumatic diseases presented in cases by 1000 patients/year**

| **Active principle** | **Disease** | **Biotherapeutic** | **Adverse events** | **Cases *1000 patients - year** | **Source of information** | **Directionality** | **n** | **Patients - year** | **Country** | **Date** | **Reference** |
| --- | --- | --- | --- | --- | --- | --- | --- | --- | --- | --- | --- |
| Abatacept | RA | Orencia | Ambulatory patients influenza / pneumonia | 0,147 | US-based Truven MarketScan and MarketScan Medicare populations | Retrospective | 1807 | 1119 | Estados Unidos | 2019 | 166 |
| Abatacept | RA | Orencia | Bone and joint infections | 0,014 | Truven Health MarketScan Commercial y supplemental Medicare US databases | Prospective | 5752 | 4333 | Estados Unidos | 2019 | 163 |
| Abatacept | RA | Orencia | Breast cancer | 0,074 | Truven Health MarketScan Commercial y supplemental Medicare US databases | Prospective | 3613 | 7208 | Estados Unidos | 2019 | 163 |
| Abatacept | RA | Orencia | Breast cancer | 0,034 | British Columbia - Administrative data of medical visits, hospitalizations and medications | Prospective | 12337 | NS | Canada | 2008 | 145 |
| Abatacept | RA | Orencia | Breast cancer | 0,028 | National Data Bank for Rheumatic Diseases - Patient Questionnaire | Prospective | 10499 | NS | Estados Unidos | 2008 | 145 |
| Abatacept | RA | Orencia | Breast cancer | 0,014 | Norfolk Arthritis Register, Patient questionnaire and evaluation | Prospective | 523 | NS | United Kingdom | 2008 | 145 |
| Abatacept | RA | Orencia | Breast cancer | 0,011 | Sweden Early RA Register - Registros médicos electronicos y evaluación de pacientes | Prospective | 3703 | NS | Sweden | 2008 | 145 |
| Abatacept | RA | Orencia | Breast cancer | 0,016 | General Practice Research Database - Electronic medical records | NS | 14467 | NS | United Kingdom | 2008 | 145 |
| Abatacept | RA | Orencia | Bronchitis | 0,42 | US-based Truven MarketScan and MarketScan Medicare populations | Retrospective | 1807 | 1181 | Estados Unidos | 2019 | 166 |
| Abatacept | RA | Orencia | Colorectal cancer | 0,014 | British Columbia - Administrative data of medical visits, hospitalizations and medications | Prospective | 12337 | NS | Canada | 2008 | 145 |
| Abatacept | RA | Orencia | Colorectal cancer | 0,006 | National Data Bank for Rheumatic Diseases - Patient Questionnaire | Prospective | 10499 | NS | Estados Unidos | 2008 | 145 |
| Abatacept | RA | Orencia | Colorectal cancer | 0,005 | Norfolk Arthritis Register, Patient questionnaire and evaluation | Prospective | 523 | NS | United Kingdom | 2008 | 145 |
| Abatacept | RA | Orencia | Colorectal cancer | 0,006 | Sweden Early RA Register - Registros médicos electronicos y evaluación de pacientes | Prospective | 3703 | NS | Sweden | 2008 | 145 |
| Abatacept | RA | Orencia | Colorectal cancer | 0,005 | General Practice Research Database - Electronic medical records | NS | 14467 | NS | United Kingdom | 2008 | 145 |
| Abatacept | RA | Orencia | Combined respiratory descent† | 0,87 | US-based Truven MarketScan and MarketScan Medicare populations | Retrospective | 1807 | 1164 | Estados Unidos | 2019 | 166 |
| Abatacept | RA | Orencia | Gastrointestinal infections | 0,065 | Truven Health MarketScan Commercial y supplemental Medicare US databases | Prospective | 5752 | 4333 | Estados Unidos | 2019 | 163 |
| Abatacept | RA | Orencia | Genitourinary tract infections | 0,042 | Truven Health MarketScan Commercial y supplemental Medicare US databases | Prospective | 5752 | 4333 | Estados Unidos | 2019 | 163 |
| Abatacept | RA | Orencia | Lung cancer | 0,024 | Truven Health MarketScan Commercial y supplemental Medicare US databases | Prospective | 4328 | 8596 | Estados Unidos | 2019 | 163 |
| Abatacept | RA | Orencia | Lung cancer | 0,026 | British Columbia - Administrative data of medical visits, hospitalizations and medications | Prospective | 12337 | NS | Canada | 2008 | 145 |
| Abatacept | RA | Orencia | Lung cancer | 0,012 | National Data Bank for Rheumatic Diseases - Patient Questionnaire | Prospective | 10499 | NS | Estados Unidos | 2008 | 145 |
| Abatacept | RA | Orencia | Lung cancer | 0,009 | Norfolk Arthritis Register, Patient questionnaire and evaluation | Prospective | 523 | NS | United Kingdom | 2008 | 145 |
| Abatacept | RA | Orencia | Lung cancer | 0,013 | Sweden Early RA Register - Registros médicos electronicos y evaluación de pacientes | Prospective | 3703 | NS | Sweden | 2008 | 145 |
| Abatacept | RA | Orencia | Lung cancer | 0,014 | General Practice Research Database - Electronic medical records | NS | 14467 | NS | United Kingdom | 2008 | 145 |
| Abatacept | RA | Orencia | Lymphoma | 0,024 | Truven Health MarketScan Commercial y supplemental Medicare US databases | Prospective | 4328 | 8596 | Estados Unidos | 2019 | 163 |
| Abatacept | RA | Orencia | Lymphoma | 0,011 | British Columbia - Administrative data of medical visits, hospitalizations and medications | Prospective | 12337 | NS | Canada | 2008 | 145 |
| Abatacept | RA | Orencia | Lymphoma | 0,008 | National Data Bank for Rheumatic Diseases - Patient Questionnaire | Prospective | 10499 | NS | Estados Unidos | 2008 | 145 |
| Abatacept | RA | Orencia | Lymphoma | 0,007 | Norfolk Arthritis Register, Patient questionnaire and evaluation | Prospective | 523 | NS | United Kingdom | 2008 | 145 |
| Abatacept | RA | Orencia | Lymphoma | 0,006 | Sweden Early RA Register - Registros médicos electronicos y evaluación de pacientes | Prospective | 3703 | NS | Sweden | 2008 | 145 |
| Abatacept | RA | Orencia | Lymphoma | 0,006 | General Practice Research Database - Electronic medical records | NS | 14467 | NS | United Kingdom | 2008 | 145 |
| Abatacept | RA | Orencia | Myeloma | 0,009 | Truven Health MarketScan Commercial y supplemental Medicare US databases | Prospective | 4328 | 8596 | Estados Unidos | 2019 | 163 |
| Abatacept | RA | Orencia | Non melanoma skin cancer | 0,212 | Truven Health MarketScan Commercial y supplemental Medicare US databases | Prospective | 4328 | 8596 | Estados Unidos | 2019 | 163 |
| Abatacept | RA | Orencia | Severe COPD exacerbation | 0,12 | US-based Truven MarketScan and MarketScan Medicare populations | Retrospective | 1807 | 1195 | Estados Unidos | 2019 | 166 |
| Abatacept | RA | Orencia | Severe pneumonia / influenza | 0,36 | US-based Truven MarketScan and MarketScan Medicare populations | Retrospective | 1807 | 1188 | Estados Unidos | 2019 | 166 |
| Abatacept | RA | Orencia | Skin infections | 0,048 | Truven Health MarketScan Commercial y supplemental Medicare US databases | Prospective | 5752 | 4333 | Estados Unidos | 2019 | 163 |
| Abatacept | RA | Orencia | Total malignancies (excluding non-melanoma skin cancer) | 0,177 | British Columbia - Administrative data of medical visits, hospitalizations and medications | Prospective | 12337 | NS | Canada | 2008 | 145 |
| Abatacept | RA | Orencia | Total malignancies (excluding non-melanoma skin cancer) | 0,073 | Norfolk Arthritis Register, Patient questionnaire and evaluation | Prospective | 523 | NS | United Kingdom | 2008 | 145 |
| Abatacept | RA | Orencia | Total malignancies (excluding non-melanoma skin cancer) | 0,071 | Sweden Early RA Register - Registros médicos electronicos y evaluación de pacientes | Prospective | 3703 | NS | Sweden | 2008 | 145 |
| Abatacept | RA | Orencia | Total malignancies (excluding non-melanoma skin cancer) | 0,067 | General Practice Research Database - Electronic medical records | NS | 14467 | NS | United Kingdom | 2008 | 145 |
| Abatacept | RA | Orencia | Upper respiratory infections and pneumonia | 0,15 | Truven Health MarketScan Commercial y supplemental Medicare US databases | Prospective | 5752 | 4333 | Estados Unidos | 2019 | 163 |
| Adalimumab | RA | Humira | Blood dyscrasias | 0,008 | FDA | Retrospective | NS | 78522 | Estados Unidos | 2006 | 165 |
| Adalimumab | RA | Humira | Congestive heart failure | 0,006 | FDA | Retrospective | NS | 78522 | Estados Unidos | 2006 | 165 |
| Adalimumab | RA | Humira | Demyelinating disease | 0,001 | FDA | Retrospective | NS | 78522 | Estados Unidos | 2006 | 165 |
| Adalimumab | RA | NS | Lupus-like events | 0,63 | BSRBR-RA | Prospective | 4362 | 17343 | United Kingdom | 2017 | 160 |
| Adalimumab | RA | Humira | Lymphoma | 0,004 | FDA | Retrospective | NS | 78522 | Estados Unidos | 2006 | 165 |
| Adalimumab | RA | Humira | Opportunistic infections | 0,006 | FDA | Retrospective | NS | 78522 | Estados Unidos | 2006 | 165 |
| Adalimumab | RA | NS | Serious adverse reactions | 50 | BiobadaBrasil | Prospective | 1372 | NS | Brazil | 2013 | 164 |
| Adalimumab | RA | NS | Serious infections | 52 | GISEA | Ambispectivo | 802 | NS | Italy | 2012 | 111 |
| Adalimumab | RA | NS | Serious infections | 17 | BiobadaBrasil | Prospective | 1372 | NS | Brazil | 2013 | 164 |
| Adalimumab | RA | NS | Serious infections - Cardiovascular | 0 | GISEA | Ambispectivo | 802 | NS | Italy | 2012 | 111 |
| Adalimumab | RA | NS | Serious infections - Intra-abdomina | 0 | GISEA | Ambispectivo | 802 | NS | Italy | 2012 | 111 |
| Adalimumab | RA | NS | Serious infections - Lower respiratory tract | 0 | GISEA | Ambispectivo | 802 | NS | Italy | 2012 | 111 |
| Adalimumab | RA | NS | Serious infections - Ocular | 0 | GISEA | Ambispectivo | 802 | NS | Italy | 2012 | 111 |
| Adalimumab | RA | NS | Serious infections - Osteoarticular | 0 | GISEA | Ambispectivo | 802 | NS | Italy | 2012 | 111 |
| Adalimumab | RA | NS | Serious infections - Sepsis | 0 | GISEA | Ambispectivo | 802 | NS | Italy | 2012 | 111 |
| Adalimumab | RA | NS | Serious infections - Skin and soft tissue | 0 | GISEA | Ambispectivo | 802 | NS | Italy | 2012 | 111 |
| Adalimumab | RA | NS | Serious infections - Tuberculosis | 0 | GISEA | Ambispectivo | 802 | NS | Italy | 2012 | 111 |
| Adalimumab | RA | NS | Serious infections - Upper respiratory tract | 0 | GISEA | Ambispectivo | 802 | NS | Italy | 2012 | 111 |
| Adalimumab | RA | NS | Serious infections - Urinary tract | 0 | GISEA | Ambispectivo | 802 | NS | Italy | 2012 | 111 |
| Adalimumab | RA | Humira | Systemic lupus erythematosus | 0,003 | FDA | Retrospective | NS | 78522 | Estados Unidos | 2006 | 165 |
| Adalimumab | RA | NS | TB | 1,57 | BSRBR-RA | Prospective | 4857 | 7634 | United Kingdom | 2010 | 155 |
| Adalimumab | RA | NS | TB | 1,76 | BIOBADASER | Prospective | NS | 565 | Spain | 2007 | 168 |
| Adalimumab | RA | Humira | TB | 0,002 | FDA | Retrospective | NS | 78522 | Estados Unidos | 2006 | 165 |
| Adalimumab | RA | NS | Vasculitis-like event | 1,05 | BSRBR-RA | Prospective | 4312 | 17172 | United Kingdom | 2017 | 160 |
| Adalimumab | PsA | NS | Serious adverse reactions | 50 | BiobadaBrasil | Prospective | NS | 495£ | Brazil | 2013 | 164 |
| Adalimumab | PsA | NS | Serious infections | 17 | BiobadaBrasil | Prospective | NS | 495£ | Brazil | 2013 | 164 |
| Adalimumab | AS | NS | Serious adverse reactions | 50 | BiobadaBrasil | Prospective | NS | 495£ | Brazil | 2013 | 164 |
| Adalimumab | AS | NS | Serious infections | 17 | BiobadaBrasil | Prospective | NS | 495£ | Brazil | 2013 | 164 |
| Adalimumab | RD | NS | Demyelination | 0,32 | BIOBADASER | Prospective | 9256 | 21425 | Spain | 2011 | 154 |
| Adalimumab | JIA | NS | Serious infections | 9,7 | Registry BIKER | NS | NS | 206 | Germany | 2014 | 187 |
| Etanercept | RA | Enbrel | Angina pectoris | 0,7 | NS | Prospective | 1073 | 2694 | Sweden | 2005 | 156 |
| Etanercept | RA | Enbrel | Asystole | 0,4 | NS | Prospective | 1073 | 2694 | Sweden | 2005 | 156 |
| Etanercept | RA | Enbrel | Benign gastrointestinal neoplasm | 0,4 | NS | Prospective | 1073 | 2694 | Sweden | 2005 | 156 |
| Etanercept | RA | Enbrel | Benign respiratory tract neoplasm | 0,7 | NS | Prospective | 1073 | 2694 | Sweden | 2005 | 156 |
| Etanercept | RA | Enbrel | Cerebral infarction | 0,4 | NS | Prospective | 1073 | 2694 | Sweden | 2005 | 156 |
| Etanercept | RA | Enbrel | Cervical cancer | 0,4 | NS | Prospective | 1073 | 2694 | Sweden | 2005 | 156 |
| Etanercept | RA | Enbrel | Coronary artery disorder | 0,4 | NS | Prospective | 1073 | 2694 | Sweden | 2005 | 156 |
| Etanercept | RA | NS | Cutaneous squamous cell carcinoma | 0,1 | Post-marketing studies, spontaneous and requested reports | Retrospective | >125000 | 3E+05 | Estados Unidos | 2005 | 161 |
| Etanercept | RA | Enbrel | Dysarthria | 0,4 | NS | Prospective | 1073 | 2694 | Sweden | 2005 | 156 |
| Etanercept | RA | Enbrel | Encephalitis | 0,4 | NS | Prospective | 1073 | 2694 | Sweden | 2005 | 156 |
| Etanercept | RA | Enbrel | Facial paresis | 0,4 | NS | Prospective | 1073 | 2694 | Sweden | 2005 | 156 |
| Etanercept | RA | NS | Fatal | 0,13 | 7 Sweden centers | Prospective | 166 | NS | Sweden | 2002 | 156 |
| Etanercept | RA | Enbrel | Gastroenteritis | 0,7 | NS | Prospective | 1073 | 2694 | Sweden | 2005 | 156 |
| Etanercept | RA | Enbrel | Headache | 0,4 | NS | Prospective | 1073 | 2694 | Sweden | 2005 | 156 |
| Etanercept | RA | Enbrel | Heart failure | 0,4 | NS | Prospective | 1073 | 2694 | Sweden | 2005 | 156 |
| Etanercept | RA | Enbrel | Infectious arthritis | 0,7 | NS | Prospective | 1073 | 2694 | Sweden | 2005 | 156 |
| Etanercept | RA | Enbrel | Lethal | 0 | NS | Prospective | 1073 | 2694 | Sweden | 2005 | 156 |
| Etanercept | RA | Enbrel | Leukopenia | 4,5 | NS | Prospective | 1073 | 2694 | Sweden | 2005 | 156 |
| Etanercept | RA | NS | Life threatening | 0 | 7 Sweden centers | Prospective | 166 | NS | Sweden | 2002 | 157 |
| Etanercept | RA | Enbrel | Life threatening | 0 | NS | Prospective | 1073 | 2694 | Sweden | 2005 | 156 |
| Etanercept | RA | NS | Lupus-like events | 0,93 | BSRBR-RA | Prospective | 4516 | 21595 | United Kingdom | 2017 | 160 |
| Etanercept | RA | Enbrel | Lymphoma | 1,1 | NS | Prospective | 1073 | 2694 | Sweden | 2005 | 156 |
| Etanercept | RA | NS | Mild | 2,7 | 7 Sweden centers | Prospective | 166 | NS | Sweden | 2002 | 157 |
| Etanercept | RA | Enbrel | Mildly serious | 0 | NS | Prospective | 1073 | 2694 | Sweden | 2005 | 156 |
| Etanercept | RA | NS | Moderate | 1,6 | 7 Sweden centers | Prospective | 166 | NS | Sweden | 2002 | 157 |
| Etanercept | RA | Enbrel | Moderately serious | 0 | NS | Prospective | 1073 | 2694 | Sweden | 2005 | 156 |
| Etanercept | RA | Enbrel | Myelodysplastic syndrome | 0,4 | NS | Prospective | 1073 | 2694 | Sweden | 2005 | 156 |
| Etanercept | RA | Enbrel | Myocardial infarction | 1,9 | NS | Prospective | 1073 | 2694 | Sweden | 2005 | 156 |
| Etanercept | RA | NS | Not graded | 0,2 | 7 Sweden centers | Prospective | 166 | NS | Sweden | 2002 | 157 |
| Etanercept | RA | Enbrel | Osteitis | 1,1 | NS | Prospective | 1073 | 2694 | Sweden | 2005 | 156 |
| Etanercept | RA | Enbrel | Other haematological disorders | 0,4 | NS | Prospective | 1073 | 2694 | Sweden | 2005 | 156 |
| Etanercept | RA | Enbrel | Other vascular disorder | 0,4 | NS | Prospective | 1073 | 2694 | Sweden | 2005 | 156 |
| Etanercept | RA | Enbrel | Ovarian cancer | 0,4 | NS | Prospective | 1073 | 2694 | Sweden | 2005 | 156 |
| Etanercept | RA | Enbrel | Pancytopenia | 0,4 | NS | Prospective | 1073 | 2694 | Sweden | 2005 | 156 |
| Etanercept | RA | Enbrel | Paraesthesiae | 0,7 | NS | Prospective | 1073 | 2694 | Sweden | 2005 | 156 |
| Etanercept | RA | Enbrel | Pneumonia | 3 | NS | Prospective | 1073 | 2694 | Sweden | 2005 | 156 |
| Etanercept | RA | Enbrel | Primary liver cancer | 0,4 | NS | Prospective | 1073 | 2694 | Sweden | 2005 | 156 |
| Etanercept | RA | Enbrel | Pulmonary oedema | 0,7 | NS | Prospective | 1073 | 2694 | Sweden | 2005 | 156 |
| Etanercept | RA | Enbrel | Rectal cancer | 0,4 | NS | Prospective | 1073 | 2694 | Sweden | 2005 | 156 |
| Etanercept | RA | Enbrel | Recurrent fever | 0,4 | NS | Prospective | 1073 | 2694 | Sweden | 2005 | 156 |
| Etanercept | RA | Enbrel | Sepsis | 3 | NS | Prospective | 1073 | 2694 | Sweden | 2005 | 156 |
| Etanercept | RA | NS | Serious | 0,7 | 7 Sweden centers | Prospective | 166 | NS | Sweden | 2002 | 157 |
| Etanercept | RA | Enbrel | Serious | 0 | NS | Prospective | 1073 | 2694 | Sweden | 2005 | 156 |
| Etanercept | RA | NS | Serious adverse reactions | 58 | BiobadaBrasil | Prospective | 1372 | NS | Brazil | 2013 | 164 |
| Etanercept | RA | NS | Serious infections | 0 | GISEA | Ambispectivo | 1130 | NS | Italy | 2012 | 111 |
| Etanercept | RA | NS | Serious infections | 30 | BiobadaBrasil | Prospective | 1372 | NS | Brazil | 2013 | 164 |
| Etanercept | RA | NS | Serious infections - Cardiovascular | 0 | GISEA | Ambispectivo | 1130 | NS | Italy | 2012 | 111 |
| Etanercept | RA | NS | Serious infections - Intra-abdomina | 0 | GISEA | Ambispectivo | 1130 | NS | Italy | 2012 | 111 |
| Etanercept | RA | NS | Serious infections - Lower respiratory tract | 0 | GISEA | Ambispectivo | 1130 | NS | Italy | 2012 | 111 |
| Etanercept | RA | NS | Serious infections - Ocular | 0 | GISEA | Ambispectivo | 1130 | NS | Italy | 2012 | 111 |
| Etanercept | RA | NS | Serious infections - Osteoarticular | 0 | GISEA | Ambispectivo | 1130 | NS | Italy | 2012 | 111 |
| Etanercept | RA | NS | Serious infections - Sepsis | 0 | GISEA | Ambispectivo | 1130 | NS | Italy | 2012 | 111 |
| Etanercept | RA | NS | Serious infections - Skin and soft tissue | 0 | GISEA | Ambispectivo | 1130 | NS | Italy | 2012 | 111 |
| Etanercept | RA | NS | Serious infections - Tuberculosis | 0 | GISEA | Ambispectivo | 1130 | NS | Italy | 2012 | 111 |
| Etanercept | RA | NS | Serious infections - Upper respiratory tract | 0 | GISEA | Ambispectivo | 1130 | NS | Italy | 2012 | 111 |
| Etanercept | RA | NS | Serious infections - Urinary tract | 0 | GISEA | Ambispectivo | 1130 | NS | Italy | 2012 | 111 |
| Etanercept | RA | Enbrel | Skin inflammation | 0,4 | NS | Prospective | 1073 | 2694 | Sweden | 2005 | 156 |
| Etanercept | RA | Enbrel | Soft tissue abscess | 0,7 | NS | Prospective | 1073 | 2694 | Sweden | 2005 | 156 |
| Etanercept | RA | NS | Stroke | 11,5 | LAUNCH | Prospective | 234 | NS | Greece | 2016 | 169 |
| Etanercept | RA | Enbrel | Subarachnoid haemorrhage | 0,7 | NS | Prospective | 1073 | 2694 | Sweden | 2005 | 156 |
| Etanercept | RA | Enbrel | Tachycardia | 0,4 | NS | Prospective | 1073 | 2694 | Sweden | 2005 | 156 |
| Etanercept | RA | NS | TB | 0,4 | BSRBR-RA | Prospective | 5521 | 12744 | United Kingdom | 2010 | 155 |
| Etanercept | RA | NS | TB | 1,14 | BIOBADASER | Prospective | NS | 1740 | Spain | 2007 | 158 |
| Etanercept | JIA | NS | Herpes zoster | 4,2 | Registry BIKER | NS | NS | 3036 | Germany | 2014 | 159 |
| Etanercept | JIA | NS | Important medical infections (intravenous antibiotic terpia or hospitalization) | 0,03 | NS | Prospective | 58 | 318 | Estados Unidos | 2008 | 132 |
| Etanercept | JIA | NS | Inflammatory bowel diseasel | 36,2 | National registries of the Netherlands, Germany, Finland, Denmark and Italy | Retrospective | 1651 | NS | International | 2011 | 168 |
| Etanercept | RA | NS | TB | 8 | ARTIS | Prospective | 2500 | 1722 | Sweden | 2005 | 153 |
| Etanercept | RA | Enbrel | Thrombocytopenia | 0,7 | NS | Prospective | 1073 | 2694 | Sweden | 2005 | 156 |
| Etanercept | RA | Enbrel | Unspecified liver neoplasm | 0,4 | NS | Prospective | 1073 | 2694 | Sweden | 2005 | 156 |
| Etanercept | RA | NS | Vasculitis-like event | 1,74 | BSRBR-RA | Prospective | 4450 | 21320 | United Kingdom | 2017 | 160 |
| Etanercept | RA | Enbrel | Venous thrombosis | 0,4 | NS | Prospective | 1073 | 2694 | Sweden | 2005 | 156 |
| Etanercept | PsA | NS | Serious adverse reactions | 58 | BiobadaBrasil | Prospective | NS | 495£ | Brazil | 2013 | 164 |
| Etanercept | PsA | NS | Serious infections | 30 | BiobadaBrasil | Prospective | NS | 495£ | Brazil | 2013 | 164 |
| Etanercept | AS | NS | Serious adverse reactions | 58 | BiobadaBrasil | Prospective | NS | 495£ | Brazil | 2013 | 164 |
| Etanercept | AS | NS | Serious infections | 30 | BiobadaBrasil | Prospective | NS | 495£ | Brazil | 2013 | 164 |
| Etanercept | RD | NS | Demyelination | 0,43 | BIOBADASER | Prospective | 9256 | 21425 | Spain | 2011 | 154 |
| Etanercept | RD | NS | Optic neuritis | 0,28 | BIOBADASER | Prospective | 9256 | 21425 | Spain | 2011 | 154 |
| Etanercept | JIA | NS | Serious adverse event | 0,12 | NS | Prospective | 58 | 318 | Estados Unidos | 2008 | 132 |
| Etanercept | JIA | NS | Serious infections | 8,1 | Registry BIKER | NS | NS | 2598 | Germany | 2014 | 159 |
| Infliximab | RA | NS | Benign, malignant and unspecified neoplasms | 3,8 | Post-marketing studies | Prospective | 5000 | NS | Japan | 2008 | 147 |
| Infliximab | RA | NS | Blood and lymphatic disorders | 3,8 | Post-marketing studies | Prospective | 5000 | NS | Japan | 2008 | 147 |
| Infliximab | RA | NS | Cardiac disorders | 11,9 | Post-marketing studies | Prospective | 5000 | NS | Japan | 2008 | 147 |
| Infliximab | RA | NS | Ear and labyrinth alterations | 0,8 | Post-marketing studies | Prospective | 5000 | NS | Japan | 2008 | 147 |
| Infliximab | RA | NS | Endocrine disorders | 0,4 | Post-marketing studies | Prospective | 5000 | NS | Japan | 2008 | 147 |
| Infliximab | RA | NS | Eye disorders | 3 | Post-marketing studies | Prospective | 5000 | NS | Japan | 2008 | 147 |
| Infliximab | RA | NS | Fatal | 0 | 7 Sweden centers | Prospective | 135 | NS | Sweden | 2002 | 157 |
| Infliximab | RA | NS | Gastrointestinal disorders | 52,6 | Post-marketing studies | Prospective | 5000 | NS | Japan | 2008 | 147 |
| Infliximab | RA | NS | Hepatobiliary Disorders | 38,6 | Post-marketing studies | Prospective | 5000 | NS | Japan | 2008 | 147 |
| Infliximab | RA | NS | Immune system disorders | 4,2 | Post-marketing studies | Prospective | 5000 | NS | Japan | 2008 | 147 |
| Infliximab | RA | NS | Infections and infestations | 183,5 | Post-marketing studies | Prospective | 5000 | NS | Japan | 2008 | 147 |
| Infliximab | RA | NS | Laboratories | 94,5 | Post-marketing studies | Prospective | 5000 | NS | Japan | 2008 | 147 |
| Infliximab | RA | NS | Life threatening | 0,28 | 7 Sweden centers | Prospective | 135 | NS | Sweden | 2002 | 157 |
| Infliximab | RA | NS | Listeriosis | 0,061 | FDA | Retrospective | NS | NS | Estados Unidos | 2003 | 146 |
| Infliximab | RA | NS | Metabolic and nutritional disorderss | 3,4 | Post-marketing studies | Prospective | 5000 | NS | Japan | 2008 | 147 |
| Infliximab | RA | NS | Mild | 5,4 | 7 Sweden centers | Prospective | 135 | NS | Sweden | 2002 | 157 |
| Infliximab | RA | NS | Moderate | 3,1 | 7 Sweden centers | Prospective | 135 | NS | Sweden | 2002 | 157 |
| Infliximab | RA | NS | Musculoskeletal and connective tissue disorders | 16,1 | Post-marketing studies | Prospective | 5000 | NS | Japan | 2008 | 147 |
| Infliximab | RA | NS | Nervous system disorders | 81 | Post-marketing studies | Prospective | 5000 | NS | Japan | 2008 | 147 |
| Infliximab | RA | NS | Psychiatric disorders | 1,3 | Post-marketing studies | Prospective | 5000 | NS | Japan | 2008 | 147 |
| Infliximab | RA | NS | Renal and urinary disorders | 5,1 | Post-marketing studies | Prospective | 5000 | NS | Japan | 2008 | 147 |
| Infliximab | RA | NS | Reproductive system and breast disorders | 0,8 | Post-marketing studies | Prospective | 5000 | NS | Japan | 2008 | 147 |
| Infliximab | PsA | NS | Serious adverse reactions | 64 | BiobadaBrasil | Prospective | NS | 495£ | Brazil | 2013 | 164 |
| Infliximab | PsA | NS | Serious infections | 31 | BiobadaBrasil | Prospective | NS | 495£ | Brazil | 2013 | 164 |
| Infliximab | AS | NS | Serious adverse reactions | 64 | BiobadaBrasil | Prospective | NS | 495£ | Brazil | 2013 | 164 |
| Infliximab | AS | NS | Serious infections | 31 | BiobadaBrasil | Prospective | NS | 495£ | Brazil | 2013 | 164 |
| Infliximab | RD | NS | Demyelination | 0,44 | BIOBADASER | Prospective | 9256 | 21425 | Spain | 2011 | 154 |
| Infliximab | RD | NS | Multiple sclerosis | 0,09 | BIOBADASER | Prospective | 9256 | 21425 | Spain | 2011 | 154 |
| Infliximab | RD | NS | Optic neuritis | 0,18 | BIOBADASER | Prospective | 9256 | 21425 | Spain | 2011 | 154 |
| Infliximab | RA | NS | Respiratory, thoracic and medicinal disorders | 59,8 | Post-marketing studies | Prospective | 5000 | NS | Japan | 2008 | 147 |
| Infliximab | RA | NS | Serious | 1 | 7 Sweden centers | Prospective | 135 | NS | Sweden | 2002 | 157 |
| Infliximab | RA | NS | Serious adverse reactions | 64 | BiobadaBrasil | Prospective | 1372 | NS | Brazil | 2013 | 164 |
| Infliximab | RA | NS | Serious infections | 0 | GISEA | Ambispectivo | 837 | NS | Italy | 2012 | 111 |
| Infliximab | RA | NS | Serious infections | 31 | BiobadaBrasil | Prospective | 1372 | NS | Brazil | 2013 | 164 |
| Infliximab | RA | NS | Serious infections - Cardiovascular | 0 | GISEA | Ambispectivo | 837 | NS | Italy | 2012 | 111 |
| Infliximab | RA | NS | Serious infections - Intra-abdomina | 0 | GISEA | Ambispectivo | 837 | NS | Italy | 2012 | 111 |
| Infliximab | RA | NS | Serious infections - Lower respiratory tract | 0 | GISEA | Ambispectivo | 837 | NS | Italy | 2012 | 111 |
| Infliximab | RA | NS | Serious infections - Ocular | 0 | GISEA | Ambispectivo | 837 | NS | Italy | 2012 | 111 |
| Infliximab | RA | NS | Serious infections - Osteoarticular | 0 | GISEA | Ambispectivo | 837 | NS | Italy | 2012 | 111 |
| Infliximab | RA | NS | Serious infections - Sepsis | 0 | GISEA | Ambispectivo | 837 | NS | Italy | 2012 | 111 |
| Infliximab | RA | NS | Serious infections - Skin and soft tissue | 0 | GISEA | Ambispectivo | 837 | NS | Italy | 2012 | 111 |
| Infliximab | RA | NS | Serious infections - Tuberculosis | 0 | GISEA | Ambispectivo | 837 | NS | Italy | 2012 | 111 |
| Infliximab | RA | NS | Serious infections - Upper respiratory tract | 0 | GISEA | Ambispectivo | 837 | NS | Italy | 2012 | 111 |
| Infliximab | RA | NS | Serious infections - Urinary tract | 0 | GISEA | Ambispectivo | 837 | NS | Italy | 2012 | 111 |
| Infliximab | RA | NS | Skin and subcutaneous tissue disorders | 134,4 | Post-marketing studies | Prospective | 5000 | NS | Japan | 2008 | 147 |
| Infliximab | RA | NS | Systematic and site administration alterations | 116,6 | Post-marketing studies | Prospective | 5000 | NS | Japan | 2008 | 147 |
| Infliximab | RA | NS | TB | 1,47 | BSRBR-RA | Prospective | 3718 | 8069 | United Kingdom | 2010 | 155 |
| Infliximab | RA | NS | TB | 3,83 | BIOBADASER | Prospective | NS | 1303 | Spain | 2007 | 158 |
| Infliximab | RA | NS | TB | 10,5 | ARTIS | Prospective | 2500 | 1722 | Sweden | 2005 | 153 |
| Infliximab | RA | NS | Vascular alterations | 48,7 | Post-marketing studies | Prospective | 5000 | NS | Japan | 2008 | 147 |
| Rituximab | RA | NS | Death | 6,9 | LAUNCH | Prospective | 234 | NS | Greece | 2016 | 169 |
| Rituximab | RA | NS | Discontinuation due to AR | 29,9 | LAUNCH | Prospective | 234 | NS | Greece | 2016 | 169 |
| Rituximab | RA | NS | Infections | 170 | LAUNCH | Prospective | 234 | NS | Greece | 2016 | 169 |
| Rituximab | RA | NS | Infections | 757 | Global Clinical Trial Program | Prospective | 3595 | NS | International | 2015 | 149 |
| Rituximab | RA | NS | Neoplasms | 4,6 | LAUNCH | Prospective | 234 | NS | Greece | 2016 | 169 |
| Rituximab | RA | NS | Psoriasis | 1,04 | AIR | Retrospective | 1927 | 1921 | France | 2012 | 167 |
| Rituximab | RA | NS | Psoriatic outbreak | 2,6 | AIR | Retrospective | 1927 | 1921 | France | 2012 | 167 |
| Rituximab | RA | NS | Reactions associated infusion | 46,1 | LAUNCH | Prospective | 234 | NS | Greece | 2016 | 169 |
| Rituximab | RA | NS | Respiratory Infection | 96,7 | LAUNCH | Prospective | 234 | NS | Greece | 2016 | 169 |
| Rituximab | RA | NS | Serious infections | 25,3 | LAUNCH | Prospective | 234 | NS | Greece | 2016 | 169 |
| Rituximab | RA | NS | Serious infections | 37,6 | Global Clinical Trial Program | Prospective | 3595 | NS | International | 2015 | 149 |
| Rituximab | RA | NS | Urinary tract infection | 36,8 | LAUNCH | Prospective | 234 | NS | Greece | 2016 | 169 |
| Tocilizumab | RA | NS | Abnormality in lipid profile | 2,6 | Chugai Pharmaceutical Co. Ltd. | Prospective | 7901 | NS | Japan | 2014 | 131 |
| Tocilizumab | RA | Actemra | Alterations in skin and subcutaneous tissue | 137,7 | Chugai Pharmaceutical Co | NS | 3881 | NS | Japan | 2011 | 130 |
| Tocilizumab | RA | Actemra | Alterations of the central nervous system | 56,9 | Chugai Pharmaceutical Co | NS | 3881 | NS | Japan | 2011 | 130 |
| Tocilizumab | RA | NS | Anaphylaxis | 122,1 | Chugai Pharmaceutical Co. Ltd. | Prospective | 7901 | NS | Japan | 2014 | 130 |
| Tocilizumab | RA | Actemra | Benign, malignant and unspecified neoplasm | 10 | Chugai Pharmaceutical Co | NS | 3881 | NS | Japan | 2011 | 130 |
| Tocilizumab | RA | NS | Bone and joint infections | 8,4 | Chugai Pharmaceutical Co. Ltd. | Prospective | 7901 | NS | Japan | 2014 | 131 |
| Tocilizumab | RA | Actemra | Cardiac disorders | 25,1 | Chugai Pharmaceutical Co | NS | 3881 | NS | Japan | 2011 | 130 |
| Tocilizumab | RA | Actemra | Cardiac dysfunction | 2,7 | FIRST Bio study | Prospective | 846 | NS | Japan | 2017 | 125 |
| Tocilizumab | RA | NS | Cardiac dysfunction | 16,2 | Chugai Pharmaceutical Co. Ltd. | Prospective | 7901 | NS | Japan | 2014 | 131 |
| Tocilizumab | RA | Actemra | Cellulitis | 8,3 | FIRST Bio study | Prospective | 844 | NS | Japan | 2017 | 125 |
| Tocilizumab | RA | Actemra | Death | 8,3 | FIRST Bio study | Prospective | 852 | NS | Japan | 2017 | 125 |
| Tocilizumab | RA | NS | Death | 9,1 | Chugai Pharmaceutical Co. Ltd. | Prospective | 7901 | NS | Japan | 2014 | 131 |
| Tocilizumab | RA | NS | Dyslipidemia | 124,2 | Hospital Docente Padre Billini | Ambispective | 443 | NS | Dominican Republic | 2018 | 133 |
| Tocilizumab | RA | Actemra | Ear and labyrinth alterations | 3,3 | Chugai Pharmaceutical Co | NS | 3881 | NS | Japan | 2011 | 130 |
| Tocilizumab | RA | Actemra | Endocrine disorders | 2,2 | Chugai Pharmaceutical Co | NS | 3881 | NS | Japan | 2011 | 130 |
| Tocilizumab | RA | Actemra | Gastrointestinal disorders | 123,2 | Chugai Pharmaceutical Co | NS | 3881 | NS | Japan | 2011 | 130 |
| Tocilizumab | RA | NS | Gastrointestinal infections | 29,8 | Chugai Pharmaceutical Co. Ltd. | Prospective | 7901 | NS | Japan | 2014 | 131 |
| Tocilizumab | RA | Actemra | Gastrointestinal perforation | 2,7 | FIRST Bio study | Prospective | 847 | NS | Japan | 2017 | 125 |
| Tocilizumab | RA | Actemra | General and site administration alterations | 80,8 | Chugai Pharmaceutical Co | NS | 3881 | NS | Japan | 2011 | 130 |
| Tocilizumab | RA | NS | Heart failure | 3,1 | Chugai Pharmaceutical Co. Ltd. | Prospective | 7901 | NS | Japan | 2014 | 131 |
| Tocilizumab | RA | Actemra | Hepatobilar alterations | 80,7 | FIRST Bio study | Prospective | 851 | NS | Japan | 2017 | 125 |
| Tocilizumab | RA | Actemra | Hepatobilar alterations | 150 | Chugai Pharmaceutical Co | NS | 3881 | NS | Japan | 2011 | 130 |
| Tocilizumab | RA | Actemra | Herpes zoster | 0,167 | FIRST Bio study | Prospective | 845 | NS | Japan | 2017 | 125 |
| Tocilizumab | RA | NS | Herpes zoster | 0,224 | Chugai Pharmaceutical Co. Ltd. | Prospective | 7901 | NS | Japan | 2014 | 131 |
| Tocilizumab | RA | Actemra | Immune system disorders | 4,5 | Chugai Pharmaceutical Co | NS | 3881 | NS | Japan | 2011 | 130 |
| Tocilizumab | RA | Actemra | Infections | 178,1 | FIRST Bio study | Prospective | 839 | NS | Japan | 2017 | 125 |
| Tocilizumab | RA | NS | Infections | 279,8 | Chugai Pharmaceutical Co. Ltd. | Prospective | 7901 | NS | Japan | 2014 | 128 |
| Tocilizumab | RA | Actemra | Infections and infestations | 308,3 | Chugai Pharmaceutical Co | NS | 3881 | NS | Japan | 2011 | 130 |
| Tocilizumab | RA | NS | Infusion reactions | 8,4 | Chugai Pharmaceutical Co. Ltd. | Prospective | 7901 | NS | Japan | 2014 | 131 |
| Tocilizumab | RA | Actemra | Injuries, poisonings and procedural complications | 29,6 | Chugai Pharmaceutical Co | NS | 3881 | NS | Japan | 2011 | 130 |
| Tocilizumab | RA | Actemra | Interstitial lung disease | 12,5 | FIRST Bio study | Prospective | 850 | NS | Japan | 2017 | 125 |
| Tocilizumab | RA | NS | Interstitial lung disease | 9,9 | Chugai Pharmaceutical Co. Ltd. | Prospective | 7901 | NS | Japan | 2014 | 131 |
| Tocilizumab | RA | NS | Ischemic heart disease | 4,7 | Chugai Pharmaceutical Co. Ltd. | Prospective | 7901 | NS | Japan | 2014 | 131 |
| Tocilizumab | RA | Actemra | Laboratory alterations | 354,6 | Chugai Pharmaceutical Co | NS | 3881 | NS | Japan | 2011 | 130 |
| Tocilizumab | RA | Actemra | Lipid alterations | 2,7 | FIRST Bio study | Prospective | 849 | NS | Japan | 2017 | 125 |
| Tocilizumab | RA | NS | Liver disorders | 238,8 | Chugai Pharmaceutical Co. Ltd. | Prospective | 7901 | NS | Japan | 2014 | 131 |
| Tocilizumab | RA | NS | Lymphoma | 10,2 | Chugai Pharmaceutical Co. Ltd. | Prospective | 7901 | NS | Japan | 2014 | 131 |
| Tocilizumab | RA | Actemra | Malignancy | 8,3 | FIRST Bio study | Prospective | 848 | NS | Japan | 2017 | 125 |
| Tocilizumab | RA | NS | Malignancy | 3,7 | Chugai Pharmaceutical Co. Ltd. | Prospective | 7901 | NS | Japan | 2014 | 131 |
| Tocilizumab | RA | Actemra | Metabolism and nutrition disorders | 95,9 | Chugai Pharmaceutical Co | NS | 3881 | NS | Japan | 2011 | 130 |
| Tocilizumab | RA | Actemra | Musculoskeletal and connective tissue disorders | 75,3 | Chugai Pharmaceutical Co | NS | 3881 | NS | Japan | 2011 | 130 |
| Tocilizumab | RA | NS | Non-hematological malignancy | 18,3 | Chugai Pharmaceutical Co. Ltd. | Prospective | 7901 | NS | Japan | 2014 | 131 |
| Tocilizumab | RA | NS | Non-tubercoly mycobacteria | 4,4 | Chugai Pharmaceutical Co. Ltd. | Prospective | 7901 | NS | Japan | 2014 | 131 |
| Tocilizumab | RA | NS | Other infections | 19,3 | Chugai Pharmaceutical Co. Ltd. | Prospective | 7901 | NS | Japan | 2014 | 131 |
| Tocilizumab | RA | Actemra | Pneumonia | 16,7 | FIRST Bio study | Prospective | 840 | NS | Japan | 2017 | 125 |
| Tocilizumab | RA | Actemra | Pneumonia | 90,9 | Chugai Pharmaceutical Co | NS | 3881 | NS | Japan | 2011 | 130 |
| Tocilizumab | RA | NS | Pneumonia | 34,4 | Chugai Pharmaceutical Co. Ltd. | Prospective | 7901 | NS | Japan | 2014 | 131 |
| Tocilizumab | RA | NS | Pneumonia due to pneumocystis | 3,7 | Chugai Pharmaceutical Co. Ltd. | Prospective | 7901 | NS | Japan | 2014 | 131 |
| Tocilizumab | RA | Actemra | Psychiatric disorders | 6,1 | Chugai Pharmaceutical Co | NS | 3881 | NS | Japan | 2011 | 130 |
| Tocilizumab | RA | NS | Pulmonary TB | 1 | Chugai Pharmaceutical Co. Ltd. | Prospective | 7901 | NS | Japan | 2014 | 131 |
| Tocilizumab | RA | Actemra | Reproductive system and breast disorders | 2,2 | Chugai Pharmaceutical Co | NS | 3881 | NS | Japan | 2011 | 130 |
| Tocilizumab | RA | NS | Respiratory infections | 140,7 | Chugai Pharmaceutical Co. Ltd. | Prospective | 7901 | NS | Japan | 2014 | 131 |
| Tocilizumab | RA | Actemra | Respiratory, thoracic and mediastinal disorders | 115,4 | Chugai Pharmaceutical Co | NS | 3881 | NS | Japan | 2011 | 130 |
| Tocilizumab | RA | NS | Skin infections | 65,2 | Chugai Pharmaceutical Co. Ltd. | Prospective | 7901 | NS | Japan | 2014 | 131 |
| Tocilizumab | RA | Actemra | Urinary and renal disorders | 11,7 | Chugai Pharmaceutical Co | NS | 3881 | NS | Japan | 2011 | 130 |
| Tocilizumab | RA | NS | Urinary tract infections | 16,4 | Chugai Pharmaceutical Co. Ltd. | Prospective | 7901 | NS | Japan | 2014 | 131 |
| Tocilizumab | RA | Actemra | Vascular disorders | 35,1 | Chugai Pharmaceutical Co | NS | 3881 | NS | Japan | 2011 | 130 |
